# Supplementary material for: Sentinel Lymph Node Biopsy in Breast Cancer Patients Undergoing Neo-Adjuvant Chemotherapy: Clinical Experience with Node-Negative and Node-Positive Disease Prior to Systemic Therapy
Source: Cancers (Basel). 2023 Mar 11;15(6):1719. doi: 10.3390/cancers15061719 (PMC10046076; doi:10.3390/cancers15061719)
Supplement: Supplementary file 1 [file cancers-15-01719-s001.zip › cancers-2201699-supplementary.pdf]

| ID | Lombardia | Data di nascita | Data I intervento | Età I intervento | Menopausa | LATO LESIONE | SEDE LESIONE | Mammografia | Eco mam m/asc | Data biopsia | Biopsia ascella | RM | Dim pre | PET | unico /multiplo | cT | cN | cM | ER % | PgR % | KI67 % | HER2 | FISH | 1° FARMACO | 2° FARMACO | 1° FARMACO | 2° FARMACO |
|---|---|---|---|---|---|---|---|---|---|---|---|---|---|---|---|---|---|---|---|---|---|---|---|---|---|---|---|
| 1327 | 1 | 09/06/1972 | 07/05/2019 | 46 | 0 | 1 | Supero-esterno | 1 | 1 | 09/10/2018 | 0 | 0 | 33 | 1 | 1 | 2 | 1 | 0 | 0 | 0 | 45 | 3+ | ° | Antraciclina | Taxani | AC | Docetaxel + Trastuzumab |
| 28895 | 1 | 17/01/1966 | 13/11/2009 | 43 | 0 | 0 | Supero-esterno | 0 | 1 | 19/06/2009 | 0 | 0 | ° | 0 | 1 | 2 | 1 | 0 | 70 | 0 | 5 | 0/1+ | ° | Antraciclina + Taxani | ° | AT | ° |
| 51800 | 0 | 09/11/1969 | 23/10/2015 | 46 | 0 | 1 | Supero-esterno | 1 | 1 | 31/03/2015 | 0 | 0 | 35 | 1 | 1 | 2 | 1 | 0 | 0 | 0 | 90 | 3+ | ° | Antraciclina | Taxani | FEC | Docetaxel + Trastuzumab |
| 56047 | 1 | 08/03/1940 | 11/07/2017 | 76 | 1 | 0 | Supero-esterno | 1 | 1 | 21/11/2016 | 0 | 0 | ° | 1 | 1 | 4 | 1 | 0 | 0 | 0 | 60 | 3+ | ° | Antraciclina | Taxani | EC | Paclitaxel + Trastuzumab |
| 65682 | 1 | 14/07/1958 | 02/07/2019 | 61 | 1 | 0 | Supero-esterno | 0 | 1 | 21/11/2018 | 1 | 1 | 100 | 1 | 2 | 3 | 1 | 0 | 0 | 0 | 20 | 3+ | ° | Antraciclina | Taxani | AC | Docetaxel + Trastuzumab |
| 93433 | 1 | 21/12/1942 | 20/03/2018 | 75 | 1 | 0 | Supero-interno | 1 | 1 | 31/07/2018 | 0 | 0 | 30 | 1 | 1 | 2 | 0 | 0 | 0 | 0 | 80 | 0/1+ | ° | Antraciclina | Taxani | EC | Paclitaxel |
| 116640 | 1 | 28/06/1949 | 17/12/2015 | 66 | 1 | 0 | Esterno | 1 | 1 | 21/04/2015 | 0 | 0 | 28 | 1 | 2 | 2 | 1 | 0 | 90 | 0 | 22 | 2+ | 1 | Antraciclina | Taxani | AC | Docetaxel + Trastuzumab |
| 148818 | 1 | 19/09/1975 | 25/11/2014 | 39 | 1 | 0 | Supero-esterno | 1 | 1 | 15/05/2014 | 0 | 0 | 25 | 0 | 1 | 2 | 0 | 0 | 98 | 98 | 22 | 2+ | 0 | Antraciclina | Taxani | AC | Docetaxel |
| 160338 | 1 | 28/07/1967 | 17/05/2012 | 44 | 0 | 0 | Infero-central | 1 | 1 | 19/10/2011 | 0 | 0 | 80 | 0 | 1 | 3 | 0 | 0 | 0 | 0 | 60 | 0/1+ | ° | Antraciclina | Taxani | FEC | Paclitaxel |
| 211645 | 1 | 25/11/1972 | 27/02/2020 | 47 | 0 | 1 | Supero-esterno | 0 | 1 | 19/07/2019 | 1 | 0 | ° | 1 | 1 | 2 | 1 | 0 | 0 | 0 | 80 | 0/1+ | ° | Antraciclina | Taxani | AC | Paclitaxel |
| 270379 | 1 | 19/01/1963 | 03/03/2009 | 45 | 0 | 0 | Supero-esterno | 1 | 1 | 15/06/2008 | 0 | 0 | ° | 0 | 1 | 3 | 1 | 0 | 80 | 60 | 8 | 0/1+ | ° | Antraciclina + Taxani | ° | AT | ° |
| 295807 | 1 | 06/04/1972 | 22/03/2018 | 45 | 0 | 0 | Supero-esterno | 1 | 1 | 07/07/2017 | 0 | 0 | 29 | 1 | 1 | 2 | 0 | 0 | 5 | 1 | 40 | 2+ | 0 | Antraciclina | Taxani | AC | Paclitaxel |
| 308092 | 1 | 28/03/1965 | 30/11/2018 | 53 | 0 | 0 | Supero-esterno | 0 | 1 | 05/07/2018 | 1 | 0 | ° | 1 | 1 | 2 | 1 | 0 | 95 | 10 | 70 | 0/1+ | ° | Antraciclina | ° | AC | ° |
| 339309 | 1 | 10/06/1963 | 26/09/2019 | 56 | 1 | 1 | Infero-central | 1 | 1 | 14/12/2018 | 0 | 0 | 15 | 1 | 1 | 1c | 0 | 0 | 0 | 0 | 30 | 3+ | ° | Antraciclina | Taxani | AC | Paclitaxel + Trastuzumab |
| 383284 | 1 | 19/04/1931 | 04/12/2018 | 87 | 1 | 0 | Supero-interno | 0 | 1 | 01/03/2017 | 0 | 0 | 44 | 1 | 2 | 2 | 1 | 0 | 0 | 0 | 40 | 3+ | ° | Trastuzumab | ° | Trastuzumab | ° |
| 401779 | 0 | 05/06/1959 | 26/04/2016 | 56 | 1 | 0 | Supero-interno | 0 | 1 | 07/08/2016 | 0 | 0 | 46 | 1 | 2 | 2 | 1 | 0 | 0 | 0 | 70 | 0/1+ | ° | Antraciclina | Taxani | AC | Paclitaxel |
| 478527 | 1 | 02/01/1948 | 26/06/2014 | 65 | 1 | 0 | Supero-esterno | 1 | 1 | 08/11/2013 | 0 | 0 | 40 | 0 | 2 | 2 | 0 | 0 | 65 | 0 | 50 | 3+ | ° | Antraciclina | Taxani | AC | Paclitaxel + Trastuzumab |
| 512894 | 1 | 04/04/1973 | 17/05/2019 | 45 | 0 | 0 | Infero-esterno | 1 | 1 | 13/09/2018 | 1 | 0 | 21 | 0 | 1 | 2 | 1 | 0 | 40 | 0 | 20 | 3+ | ° | Antraciclina | Taxani | AC | Paclitaxel + Trastuzumab |
| 534975 | 1 | 08/12/1949 | 14/05/2009 | 60 | 1 | 1 | Supero-central | 0 | 1 | 05/12/2009 | 0 | 0 | ° | 0 | 1 | 4b | 0 | 0 | 95 | 95 | 20 | 2+ | 0 | Antraciclina + Taxani | ° | AT | ° |
| 629186 | 0 | 12/03/1982 | 14/12/2018 | 36 | 0 | 0 | Supero-esterno | 1 | 1 | 15/04/2018 | 0 | 0 | 40 | 0 | 1 | 2 | 1 | 0 | 90 | 70 | 30 | 0/1+ | ° | Antraciclina | Taxani | EC | Paclitaxel |
| 640046 | 1 | 10/03/1962 | 20/11/2008 | 46 | 0 | 1 | Supero-esterno | 1 | 1 | 15/06/2008 | 0 | 0 | 21 | 0 | 1 | 2 | 0 | 0 | ° | ° | ° | 0/1+ | ° | Antraciclina | ° | FEC | ° |
| 640720 | 1 | 13/09/1941 | 09/01/2009 | 68 | 1 | 0 | Supero-central | 0 | 1 | ° | 0 | 0 | ° | 0 | 2 | 2 | 0 | 0 | 90 | 70 | 8 | 0/1+ | ° | Antraciclina | Taxani | FEC | Docetaxel |
| 641527 | 1 | 30/11/1957 | 08/01/2009 | 52 | 1 | 0 | Supero-esterno | 0 | 1 | 01/06/2008 | 0 | 0 | 50 | 0 | 1 | 4 | 1 | 0 | 80 | 60 | 15 | 3+ | ° | Antraciclina | ° | AC | ° |
| 719368 | 1 | 18/07/1965 | 29/01/2009 | 43 | 0 | 1 | Supero-esterno | 0 | 1 | 15/07/2008 | 0 | 0 | ° | 0 | 1 | 1c | 0 | 0 | 80 | 90 | 8 | 0/1+ | ° | Antraciclina + Taxani | ° | AT | ° |
| 773928 | 0 | 07/07/1967 | 27/02/2009 | 42 | 1 | 0 | Supero-esterno | 0 | 1 | 15/09/2008 | 0 | 1 | 40 | 0 | 1 | 2 | 0 | 0 | 50 | 20 | 10 | 3+ | ° | Antraciclina | Taxani | AC | Docetaxel |
| 889128 | 0 | 14/04/1953 | 07/09/2009 | 56 | 1 | 0 | Supero-esterno | 1 | 1 | 18/03/2009 | 0 | 0 | ° | 0 | 1 | 2 | 0 | 0 | 90 | 60 | 15 | 2+ | 0 | Antraciclina + Taxani | ° | AT | ° |
| 1085227 | 1 | 23/06/1975 | 01/03/2019 | 68 | 0 | 0 | Supero-esterno | 1 | 1 | 05/07/2018 | 1 | 1 | 23 | 1 | 2 | 2 | 1 | 0 | 0 | 0 | 35 | 0/1+ | ° | Antraciclina | Taxani | AC | Paclitaxel |
| 1159327 | 1 | 10/01/1962 | 17/11/2009 | 47 | 0 | 1 | Esterno | 0 | 1 | 19/06/2009 | 0 | 0 | 40 | 0 | 1 | 2 | 0 | 0 | 90 | 90 | 30 | 0/1+ | ° | Antraciclina + Taxani | ° | AT | ° |
| 1359700 | 0 | 24/04/1947 | 02/02/2010 | 62 | 1 | 0 | Regione | 0 | 1 | 15/09/2009 | 0 | 1 | 35 | 0 | 1 | 2 | 0 | 0 | 5 | 0 | 30 | 0/1+ | ° | Antraciclina | Taxani | AC | Docetaxel |
| 1367343 | 1 | 27/02/1970 | 01/12/2009 | 39 | 0 | 1 | ° | 0 | 1 | 15/08/2009 | 0 | 0 | ° | 0 | 1 | 1c | 0 | 0 | 80 | 0 | 60 | 0/1+ | ° | Antraciclina | ° | AC | ° |
| 1373188 | 0 | 08/07/1981 | 19/03/2010 | 29 | 0 | 0 | Supero-esterno | 0 | 1 | 17/11/2009 | 0 | 0 | 30 | 0 | 1 | 2 | 1 | 0 | 10 | 4 | 60 | 2+ | 0 | Antraciclina | Taxani | FEC | Docetaxel |
| 1376641 | 1 | 05/04/1978 | 31/01/2019 | 40 | 0 | 1 | Supero-esterno | 0 | 1 | 16/05/2019 | 1 | 1 | 21 | 1 | 2 | 2 | 1 | 0 | 95 | 90 | 40 | 2+ | 1 | Antraciclina | Taxani | AC | Docetaxel + Trastuzumab |
| 1410966 | 1 | 23/05/1978 | 05/09/2018 | 40 | 0 | 0 | Supero-interno | 0 | 1 | 15/12/2017 | 0 | 0 | 15 | 1 | 1 | 1c | 0 | 0 | 90 | 40 | 43 | 3+ | ° | Antraciclina | Taxani | AC | Docetaxel + Trastuzumab |
| 1414526 | 0 | 17/05/1977 | 15/02/2011 | 33 | 0 | 0 | Supero-interno | 1 | 1 | 14/07/2010 | 0 | 0 | 28 | 0 | 1 | 2 | 0 | 0 | 0 | 0 | 60 | 3+ | ° | Antraciclina | Taxani | AC | Paclitaxel + Trastuzumab |
| 1479449 | 1 | 09/02/1973 | 10/09/2019 | 46 | 0 | 0 | Regione | 1 | 1 | 04/01/2018 | 1 | 0 | 12 | 1 | 1 | 1c | 1 | 0 | 90 | 85 | 55 | 0/1+ | ° | Antraciclina | Taxani | AC | Docetaxel |
| 1507210 | 1 | 28/10/1972 | 21/12/2011 | 39 | 0 | 0 | Regione | 0 | 1 | 07/07/2011 | 0 | 1 | ° | 1 | 1 | ° | 0 | 0 | 75 | 90 | 16 | 0/1+ | ° | Antraciclina | ° | AC | ° |
| 1562551 | 1 | 23/07/1960 | 05/02/2019 | 58 | 1 | 1 | Supero-interno | 0 | 1 | 28/06/2019 | 0 | 0 | 43 | 1 | 2 | 2 | 0 | 0 | 80 | 0 | 50 | 3+ | ° | Antraciclina | Taxani | AC | Docetaxel + Trastuzumab |
| 1568683 | 1 | 06/01/1961 | 17/02/2016 | 54 | 1 | 0 | Supero-central | 0 | 1 | 01/06/2015 | 0 | 0 | 30 | 1 | 1 | 2 | 0 | 0 | 0 | 0 | 34 | 0/1+ | ° | Antraciclina | Taxani | AC | Paclitaxel |
| 1577399 | 0 | 28/10/1975 | 28/09/2016 | 41 | 0 | 1 | Supero-central | 0 | 1 | 16/02/2016 | 0 | 0 | ° | 1 | 1 | 1c | 1 | 0 | 0 | 0 | 60 | 0/1+ | ° | Antraciclina | Taxani | AC | Paclitaxel |

| | | | | | | | | | | | | | | | | | | | | | | | | | | | |
|---|---|---|---|---|---|---|---|---|---|---|---|---|---|---|---|---|---|---|---|---|---|---|---|---|---|---|---|
| 1587345 | 0 | 04/11/1961 | 08/10/2013 | 52 | 1 | 1 | Supero-central | 1 | 1 | 21/02/2013 | 0 | 0 | 40 | 0 | 1 | 2 | 1 | 0 | 0 | 0 | 45 | 3+ | ° | Antraciclina | Taxani | AC | Docetaxel + Trastuzumab |
| 1589074 | 1 | 10/12/1965 | 12/03/2013 | 47 | 1 | 0 | Supero-central | 0 | 1 | 15/11/2012 | 0 | 0 | ° | 1 | 1 | 2 | 0 | 0 | 80 | 20 | 29 | 2+ | 0 | Antraciclina + Taxani | ° | AT | ° |
| 1596834 | 1 | 24/12/1976 | 19/12/2013 | 37 | 0 | 1 | Supero-interno | 1 | 1 | 10/04/2013 | 0 | 0 | 25 | 0 | 2 | 2 | 0 | 0 | 35 | 0 | 70 | 0/1+ | ° | Antraciclina | Taxani | AC | Docetaxel |
| 1604518 | 1 | 16/09/1956 | 24/05/2013 | 57 | 1 | 1 | Supero-esterno | 1 | 1 | 15/02/2013 | 0 | 0 | ° | 0 | 1 | 4 | 0 | 0 | 0 | 0 | 30 | 0/1+ | ° | Antraciclina | ° | EC | ° |
| 1608013 | 1 | 04/02/1968 | 09/01/2014 | 45 | 0 | 1 | Infero-esterno | 1 | 1 | 05/06/2013 | 0 | 0 | 30 | 0 | 1 | 2 | 0 | 0 | 20 | 0 | 80 | 0/1+ | ° | Antraciclina | Taxani | AC | Docetaxel |
| 1609586 | 1 | 19/03/1967 | 08/03/2018 | 50 | 0 | 0 | Infero-esterno | 1 | 1 | 21/07/2017 | 0 | 0 | 32 | 1 | 1 | 2 | 0 | 0 | 0 | 0 | 70 | 0/1+ | ° | Antraciclina | Taxani | AC | Paclitaxel |
| 1627196 | 0 | 21/03/1958 | 05/06/2014 | 55 | 1 | 0 | Regione | 1 | 1 | 16/10/2013 | 0 | 1 | 63 | 1 | 1 | 3 | 1 | 0 | 0 | 0 | 35 | 3+ | ° | Antraciclina | Taxani | AC | Docetaxel + Trastuzumab |
| 1635054 | 0 | 14/04/1969 | 29/04/2014 | 49 | 0 | 0 | Supero-esterno | 1 | 1 | 11/11/2013 | 0 | 1 | 55 | 0 | 2 | 3 | 0 | 0 | 100 | 80 | 15 | 0/1+ | ° | Antraciclina | ° | AC | ° |
| 1641158 | 1 | 15/07/1987 | 20/07/2017 | 30 | 0 | 0 | Supero-esterno | 0 | 1 | 30/09/2016 | 1 | 1 | 20 | 1 | 1 | 1c | 1 | 0 | 95 | 70 | 40 | 0/1+ | ° | Antraciclina | Taxani | AC | Docetaxel |
| 1653137 | 0 | 07/04/1950 | 07/10/2014 | 64 | 1 | 1 | Supero-esterno | 1 | 1 | 13/12/2013 | 0 | 1 | 25 | 1 | 2 | 2 | 1 | 0 | 40 | 30 | 20 | 0/1+ | ° | Antraciclina | Taxani | ET | Docetaxel |
| 1653385 | 0 | 22/01/1966 | 16/05/2014 | 48 | 0 | 1 | Regione | 0 | 1 | ° | 0 | 0 | ° | 0 | 2 | ° | 0 | 0 | ° | ° | ° | 0/1+ | ° | Antraciclina | ° | FEC | ° |
| 1658323 | 0 | 12/12/1957 | 26/08/2014 | 57 | 1 | 1 | Supero-esterno | 1 | 1 | 13/03/2014 | 0 | 1 | 55 | 0 | 1 | 3 | 0 | 0 | 90 | 1 | 5 | 0/1+ | ° | Antraciclina | ° | AC | ° |
| 1665544 | 0 | 18/05/1960 | 28/10/2014 | 54 | 1 | 1 | Diffuso | 1 | 1 | 24/03/2014 | 0 | 1 | ° | 0 | 2 | 2 | 1 | 0 | ° | ° | 40 | 3+ | ° | Taxani | Trastuzumab | Docetaxel | Trastuzumab |
| 1666359 | 0 | 01/10/1961 | 16/05/2014 | 53 | 1 | 0 | Infero-esterno | 1 | 1 | 15/09/2013 | 0 | 1 | 80 | 0 | 1 | 3 | 1 | 0 | 92 | 60 | 72 | 3+ | ° | Antraciclina | Taxani | FEC | Paclitaxel + Trastuzumab |
| 1667122 | 0 | 25/09/1974 | 29/04/2014 | 39 | 0 | 1 | Supero-esterno | 0 | 1 | ° | 0 | 0 | ° | 0 | 1 | 4 | 1 | 0 | 98 | 70 | ° | 0/1+ | ° | Antraciclina | Taxani | AC | Paclitaxel |
| 1670827 | 0 | 20/12/1967 | 25/11/2014 | 47 | 1 | 0 | Infero-esterno | 1 | 1 | 15/05/2014 | 1 | 1 | 24 | 0 | 2 | 2 | 1 | 0 | 95 | 1 | 25 | 0/1+ | ° | Antraciclina | Taxani | EC | Paclitaxel |
| 1677118 | 0 | 28/01/1977 | 20/06/2014 | 37 | 0 | 0 | Ignoto | 0 | 1 | ° | 0 | 0 | 40 | 0 | 1 | 2 | 0 | 0 | 0 | 0 | 55 | 0/1+ | ° | Antraciclina | Taxani | AC | Paclitaxel |
| 1677372 | 0 | 05/04/1965 | 27/06/2014 | 49 | 1 | 0 | Supero-interno | 1 | 1 | 15/09/2013 | 0 | 1 | 51 | 0 | 1 | 3 | 0 | 0 | 0 | 0 | 83 | 0/1+ | ° | Antraciclina | Taxani | EC | Docetaxel |
| 1681596 | 0 | 18/05/1965 | 11/02/2015 | 49 | 0 | 0 | Infero-esterno | 1 | 1 | 10/07/2014 | 0 | 0 | 32 | 1 | 2 | 2 | 1 | 0 | 70 | 0 | 30 | 3+ | ° | Antraciclina | Taxani | AC | Docetaxel + Trastuzumab |
| 1684431 | 1 | 29/09/1967 | 03/09/2019 | 52 | 1 | 0 | Supero-esterno | 0 | 1 | 20/11/2018 | 1 | 0 | 22 | 1 | 1 | 2 | 1 | 0 | 50 | 35 | 60 | 0/1+ | ° | Antraciclina | Taxani | AC | Docetaxel |
| 1687529 | 1 | 26/05/1948 | 09/12/2014 | 66 | 1 | 0 | Esterno | 0 | 1 | 15/08/2014 | 0 | 1 | 21 | 1 | 2 | 2 | 1 | 0 | 95 | 5 | 20 | 2+ | 0 | Antraciclina | ° | AC | ° |
| 1691970 | 0 | 23/01/1968 | 09/04/2015 | 46 | 0 | 1 | Supero-esterno | 1 | 1 | 02/09/2015 | 0 | 0 | 25 | 0 | 1 | 2 | 0 | 0 | 99 | 99 | 61 | 3+ | ° | Antraciclina | Taxani | AC | Paclitaxel + Trastuzumab |
| 1693528 | 0 | 15/09/1979 | 17/04/2015 | 35 | 0 | 0 | Supero-central | 1 | 1 | 23/09/2014 | 0 | 0 | 31 | 1 | 2 | 2 | 1 | 0 | 80 | 80 | 40 | 3+ | ° | Antraciclina | Taxani | AC | Docetaxel + Trastuzumab |
| 1694951 | 1 | 20/12/1959 | 11/06/2015 | 55 | 1 | 0 | Supero-esterno | 1 | 1 | 30/09/2014 | 0 | 1 | 17 | 1 | 1 | 4d | 1 | 0 | 0 | 0 | 40 | 3+ | ° | Antraciclina | Taxani | AC | Docetaxel + Trastuzumab |
| 1697298 | 0 | 04/12/1970 | 17/04/2015 | 44 | 0 | 1 | Infero-central | 1 | 1 | 25/09/2014 | 0 | 1 | 40 | 1 | 2 | 2 | 0 | 0 | 0 | 0 | 90 | 0/1+ | ° | Antraciclina | Taxani | AC | Docetaxel |
| 1703271 | 1 | 13/04/1959 | 08/05/2015 | 55 | 1 | 1 | Infero-esterno | 0 | 1 | 07/11/2014 | 0 | 0 | 53 | 1 | 2 | 3 | 0 | 0 | 90 | 30 | 50 | 0/1+ | ° | Antraciclina | ° | FEC | ° |
| 1707289 | 1 | 27/07/1944 | 11/06/2015 | 70 | 1 | 1 | Supero-esterno | 0 | 1 | 29/10/2014 | 0 | 0 | 30 | 0 | 1 | 2 | 0 | 0 | 15 | 0 | 85 | 0/1+ | ° | Antraciclina | Taxani | AC | Docetaxel |
| 1708271 | 0 | 10/02/1956 | 08/09/2015 | 59 | 1 | 1 | Regione | 1 | 1 | 05/12/2014 | 0 | 0 | ° | 1 | 2 | 2 | 1 | 0 | 90 | 35 | 15 | 0/1+ | ° | Antraciclina | Taxani | AC | Paclitaxel |
| 1711991 | 1 | 18/11/1950 | 15/09/2015 | 65 | 1 | 1 | Supero-esterno | 1 | 1 | 24/12/2014 | 0 | 1 | ° | 1 | 1 | 2 | 1 | 0 | 0 | 0 | 70 | 0/1+ | ° | Antraciclina | Taxani | EC | Paclitaxel |
| 1712285 | 0 | 16/05/1971 | 22/10/2019 | 48 | 1 | 1 | Supero-esterno | 1 | 1 | 15/03/2019 | 1 | 1 | 100 | 1 | 2 | 3 | 1 | 0 | 0 | 0 | 46 | 3+ | ° | Antraciclina | Taxani | EC | Docetaxel + Trastuzumab |
| 1712882 | 1 | 13/07/1980 | 22/07/2015 | 35 | 0 | 0 | Supero-central | 1 | 1 | 31/12/2014 | 0 | 0 | 30 | 1 | 1 | 2 | 0 | 0 | 0 | 0 | 80 | 0/1+ | ° | Antraciclina | Taxani | AC | Paclitaxel |
| 1713724 | 0 | 26/03/1946 | 18/08/2015 | 69 | 1 | 0 | Supero-esterno | 1 | 1 | 09/01/2015 | 0 | 0 | 30 | 1 | 1 | 2 | 1 | 0 | 0 | 0 | 70 | 0/1+ | ° | Antraciclina | Taxani | AC | Paclitaxel |
| 1716311 | 0 | 12/10/1964 | 04/08/2015 | 51 | 1 | 1 | Interno | 0 | 1 | 14/11/2014 | 1 | 1 | 27 | 1 | 2 | 2 | 1 | 0 | 0 | 0 | 60 | 2+ | 0 | Antraciclina | Taxani | FEC | Paclitaxel + Trastuzumab |
| 1722588 | 1 | 06/06/1978 | 04/02/2020 | 41 | 0 | 0 | Supero-esterno | 0 | 1 | 24/06/2019 | 0 | 1 | 41 | 1 | 2 | 2 | 0 | 0 | 0 | 0 | 80 | 0/1+ | ° | Antraciclina | Taxani | AC | Paclitaxel |
| 1724381 | 0 | 10/09/1949 | 12/03/2015 | 66 | 1 | 1 | Supero-interno | 1 | 1 | 15/07/2014 | 0 | 1 | 35 | 0 | 1 | 2 | 1 | 0 | 95 | 95 | 13 | 0/1+ | ° | Antraciclina | ° | FEC | ° |
| 1725624 | 1 | 26/12/1974 | 03/11/2015 | 41 | 0 | 1 | Regione | 1 | 1 | 06/03/2015 | 0 | 0 | 27 | 1 | 1 | 4 | 1 | 0 | 80 | 5 | 15 | 3+ | ° | Antraciclina | Taxani | AC | Paclitaxel |
| 1739360 | 0 | 02/03/1976 | 11/12/2015 | 39 | 0 | 0 | Supero-esterno | 1 | 1 | 17/04/2015 | 0 | 1 | 30 | 0 | 2 | 2 | 0 | 0 | 0 | 0 | 25 | 0/1+ | ° | Antraciclina | Taxani | EC | Paclitaxel |
| 1741925 | 1 | 29/01/1972 | 26/01/2016 | 43 | 0 | 0 | Supero-central | 1 | 1 | 26/05/2015 | 0 | 1 | 80 | 1 | 1 | 3 | 1 | 0 | 0 | 0 | 40 | 3+ | ° | Antraciclina | Taxani | AC | Docetaxel + Trastuzumab |
| 1745672 | 1 | 25/07/1981 | 07/03/2016 | 34 | 0 | 1 | Supero-esterno | 0 | 1 | 19/06/2015 | 0 | 0 | ° | 1 | 1 | 2 | 0 | 0 | 0 | 0 | 30 | 0/1+ | ° | Antraciclina | Taxani | AC | Paclitaxel |
| 1747942 | 1 | 17/01/1959 | 11/02/2016 | 56 | 1 | 1 | Supero-esterno | 1 | 1 | 24/06/2015 | 0 | 0 | 38 | 0 | 1 | 2 | 0 | 0 | 0 | 0 | 40 | 0/1+ | ° | Antraciclina | Taxani | AC | Paclitaxel |
| 1751767 | 0 | 28/04/1962 | 18/02/2016 | 53 | 1 | 0 | Supero-interno | 1 | 1 | 14/07/2015 | 0 | 0 | 35 | 0 | 1 | 2 | 1 | 0 | 0 | 0 | 10 | 0/1+ | ° | Antraciclina | Taxani | AC | Docetaxel |
| 1752257 | 0 | 13/08/1968 | 05/02/2016 | 47 | 0 | 1 | Supero-central | 1 | 1 | 17/07/2015 | 0 | 0 | 30 | 0 | 1 | 3 | 0 | 0 | 85 | 85 | 30 | 3+ | ° | Antraciclina | Taxani | AC | Docetaxel + Trastuzumab |

| | | | | | | | | | | | | | | | | | | | | | | | | | | | |
|---|---|---|---|---|---|---|---|---|---|---|---|---|---|---|---|---|---|---|---|---|---|---|---|---|---|---|---|
| 1754587 | 0 | 30/01/1950 | 15/03/2016 | 65 | 1 | 0 | Regione | 1 | 1 | 28/07/2015 | 0 | 1 | 25 | 1 | 1 | 2 | 0 | 0 | 90 | 90 | 5 | 0/1+ | ° | Antraciclina | Taxani | EC | Paclitaxel |
| 1754598 | 0 | 06/09/1970 | 05/05/2016 | 45 | 1 | 0 | Supero-esterno | 1 | 1 | 28/07/2015 | 0 | 1 | 30 | 1 | 2 | 2 | 0 | 0 | 90 | 35 | 75 | 2+ | 1 | Antraciclina | Taxani | AC | Paclitaxel + Trastuzumab |
| 1767290 | 0 | 22/01/1943 | 21/10/2015 | 71 | 1 | 0 | Regione | 0 | 1 | 15/11/2014 | 0 | 1 | 51 | 0 | 1 | 3 | 0 | 0 | 0 | 0 | 30 | 3+ | ° | Antraciclina | Taxani | EC | Paclitaxel + Trastuzumab |
| 1769124 | 0 | 30/08/1973 | 27/10/2015 | 42 | 0 | 0 | Infero-interno | 1 | 1 | 15/02/2015 | 0 | 1 | 54 | 0 | 2 | 3 | 0 | 0 | 98 | 80 | 28 | 0/1+ | ° | Antraciclina | Taxani | EC | Docetaxel |
| 1782343 | 1 | 24/10/1971 | 06/09/2016 | 45 | 0 | 1 | Supero-centrale | 1 | 1 | 22/12/2015 | 0 | 0 | 21 | 0 | 1 | 2 | 0 | 0 | 0 | 0 | 40 | 0/1+ | ° | Antraciclina | Taxani | AC | Paclitaxel |
| 1783909 | 1 | 04/03/1966 | 08/09/2016 | 50 | 1 | 0 | Supero-esterno | 0 | 1 | 07/01/2016 | 0 | 0 | ° | 1 | 2 | 3 | 1 | 0 | 0 | 0 | 85 | 0/1+ | ° | Antraciclina | Taxani | AC | Paclitaxel |
| 1785743 | 0 | 26/08/1970 | 13/07/2016 | 46 | 0 | 0 | Infero-esterno | 1 | 1 | 15/12/2015 | 0 | 1 | 51 | 0 | 1 | 3 | 1 | 0 | 32 | 14 | 58 | 2+ | 1 | Trastuzumab | Pertuzumab | Trastuzumab | Pertuzumab |
| 1786643 | 0 | 08/01/1957 | 22/01/2016 | 58 | 1 | 1 | Supero-esterno | 1 | 1 | 18/07/2015 | 0 | 1 | 25 | 0 | 1 | 2 | 0 | 0 | ° | ° | ° | 3+ | ° | Antraciclina | Taxani | EC | Paclitaxel + Trastuzumab |
| 1803876 | 1 | 02/11/1966 | 29/11/2016 | 50 | 0 | 0 | Interno | 0 | 1 | 06/04/2016 | 0 | 0 | ° | 1 | 1 | 2 | 1 | 0 | 0 | 0 | 20 | 3+ | ° | Antraciclina | Taxani | AC | Docetaxel + Trastuzumab |
| 1813065 | 0 | 12/03/1963 | 10/01/2017 | 53 | 0 | 0 | Supero-esterno | 1 | 1 | 18/05/2016 | 0 | 0 | ° | 1 | 1 | 2 | 1 | 0 | 90 | 30 | 30 | 0/1+ | ° | Antraciclina | Taxani | AC | Paclitaxel |
| 1815999 | 0 | 20/01/1959 | 10/06/2016 | 56 | 1 | 0 | Supero-esterno | 0 | 1 | 04/11/2015 | 0 | 0 | 17 | 0 | 1 | 1c | 0 | 0 | 98 | 95 | 40 | 3+ | ° | Antraciclina | Taxani | EC | Paclitaxel + Trastuzumab |
| 1817773 | 1 | 07/06/1979 | 16/01/2017 | 37 | 0 | 1 | Infero-esterno | 0 | 1 | 08/06/2016 | 0 | 0 | 39 | 1 | 2 | 2 | 0 | 0 | 95 | 90 | 50 | 3+ | ° | Antraciclina | Taxani | AC | Docetaxel + Trastuzumab |
| 1818700 | 1 | 13/03/1962 | 27/01/2017 | 54 | 1 | 1 | Supero-esterno | 0 | 1 | 13/06/2016 | 1 | 0 | 21 | 1 | 2 | 2 | 1 | 0 | 95 | 80 | 70 | 2+ | 1 | Antraciclina | Taxani | AC | Docetaxel + Trastuzumab |
| 1820885 | 0 | 05/03/1946 | 02/02/2017 | 71 | 1 | 1 | Supero-esterno | 1 | 1 | 22/06/2016 | 0 | 0 | ° | 1 | 1 | 2 | 1 | 0 | 90 | 35 | 30 | 2+ | 1 | Taxani | Trastuzumab | Docetaxel | Trastuzumab |
| 1823344 | 0 | 08/01/1955 | 16/01/2017 | 62 | 1 | 0 | Esterno | 1 | 1 | 04/07/2016 | 0 | 0 | 40 | 1 | 1 | 2 | 0 | 0 | 90 | 90 | 20 | 0/1+ | ° | Antraciclina | Taxani | FEC | Docetaxel |
| 1824218 | 0 | 07/11/1976 | 06/12/2016 | 40 | 0 | 0 | Infero-interno | 1 | 1 | 15/06/2016 | 0 | 1 | 22 | 1 | 2 | 2 | 0 | 0 | 95 | 15 | 42 | 3+ | ° | Antraciclina | Taxani | AC | Paclitaxel + Trastuzumab |
| 1825098 | 0 | 07/10/1965 | 15/07/2016 | 50 | 0 | 1 | Supero-centrale | 1 | 1 | 30/11/2015 | 0 | 1 | 70 | 1 | 1 | 3 | 0 | 0 | 65 | 40 | 40 | 3+ | ° | Antraciclina | Taxani | AC | Paclitaxel + Trastuzumab |
| 1826556 | 0 | 08/01/1966 | 28/07/2016 | 50 | 1 | 1 | Supero-esterno | 1 | 1 | 03/12/2015 | 0 | 1 | 35 | 1 | 1 | 2 | 1 | 0 | 0 | 0 | 90 | 3+ | ° | Antraciclina | Taxani | EC | Paclitaxel + Trastuzumab |
| 1827014 | 1 | 13/09/1971 | 01/03/2017 | 45 | 0 | 1 | Supero-esterno | 1 | 1 | 21/07/2016 | 0 | 0 | ° | 1 | 1 | 1c | 0 | 0 | 5 | 10 | 30 | 3+ | ° | Antraciclina | Taxani | AC | Docetaxel + Trastuzumab |
| 1829539 | 0 | 08/02/1969 | 20/09/2016 | 47 | 0 | 0 | Supero-esterno | 1 | 1 | 28/01/2016 | 1 | 1 | 27 | 0 | 1 | 2 | 1 | 0 | 95 | 10 | 60 | 0/1+ | ° | Antraciclina | Taxani | EC | Paclitaxel |
| 1832526 | 1 | 21/12/1968 | 24/03/2017 | 48 | 0 | 0 | Supero-interno | 1 | 1 | 02/09/2016 | 0 | 1 | 44 | 1 | 2 | 2 | 0 | 0 | 0 | 0 | 20 | 3+ | ° | Antraciclina | Taxani | AC | Docetaxel + Trastuzumab |
| 1833641 | 0 | 05/09/1970 | 21/03/2017 | 46 | 0 | 0 | Supero-esterno | 1 | 1 | 05/09/2016 | 0 | 1 | 50 | 1 | 2 | 2 | 1 | 0 | 80 | 40 | 15 | 3+ | ° | Antraciclina | Taxani | AC | Docetaxel + Trastuzumab |
| 1836019 | 1 | 13/04/1976 | 19/04/2017 | 40 | 0 | 1 | Supero-esterno | 0 | 1 | 14/09/2016 | 0 | 0 | ° | 1 | 1 | 2 | 1 | 0 | 90 | 90 | 10 | 0/1+ | ° | Antraciclina | Taxani | AC | Docetaxel |
| 1844794 | 0 | 16/03/1973 | 26/01/2017 | 43 | 0 | 0 | Supero-esterno | 1 | 1 | 07/07/2016 | 1 | 1 | 55 | 1 | 2 | 3 | 1 | 0 | 40 | 0 | 40 | 3+ | ° | Antraciclina | Taxani | AC | Paclitaxel + Trastuzumab |
| 1849840 | 1 | 14/03/1983 | 08/08/2017 | 33 | 0 | 0 | Supero-esterno | 0 | 1 | 15/11/2016 | 0 | 1 | ° | 1 | 1 | 2 | 1 | 0 | 0 | 0 | 35 | 0/1+ | ° | Antraciclina | Taxani | AC | Paclitaxel |
| 1851331 | 1 | 21/09/1971 | 24/11/2016 | 45 | 0 | 0 | Supero-centrale | 1 | 1 | 12/05/2016 | 1 | 0 | 51 | 1 | 1 | 3 | 1 | 0 | 75 | 50 | 30 | 3+ | ° | Antraciclina | Taxani | AC | Docetaxel + Trastuzumab |
| 1853846 | 1 | 21/04/1965 | 21/07/2017 | 52 | 1 | 1 | Supero-esterno | 1 | 1 | 26/11/2016 | 0 | 0 | 33 | 1 | 1 | 2 | 1 | 0 | 0 | 0 | 20 | 0/1+ | ° | Antraciclina | Taxani | AC | Paclitaxel |
| 1854348 | 1 | 21/03/1938 | 03/04/2018 | 79 | 1 | 0 | Supero-esterno | 1 | 1 | 29/09/2018 | 0 | 0 | 21 | 1 | 1 | 2 | 1 | 0 | 95 | 90 | 17 | 2+ | 1 | Taxani | Trastuzumab | Paclitaxel | Trastuzumab |
| 1855589 | 0 | 15/12/1979 | 16/12/2016 | 37 | 0 | 1 | Esterno | 1 | 1 | 18/05/2016 | 0 | 1 | 30 | 0 | 1 | 2 | 1 | 0 | 95 | 95 | 40 | 2+ | 0 | Antraciclina | Taxani | AC | Paclitaxel |
| 1861256 | 0 | 11/01/1980 | 07/08/2017 | 37 | 0 | 1 | Esterno | 1 | 1 | 30/12/2016 | 1 | 1 | 40 | 1 | 1 | 2 | 1 | 0 | 90 | 70 | 50 | 0/1+ | ° | Antraciclina | Taxani | AC | Docetaxel |
| 1862999 | 0 | 08/08/1968 | 03/10/2017 | 49 | 0 | 1 | Supero-esterno | 1 | 1 | 22/12/2016 | 0 | 0 | 27 | 1 | 1 | 2 | 1 | 0 | 0 | 0 | 60 | 0/1+ | ° | Antraciclina | Taxani | AC | Paclitaxel |
| 1864763 | 1 | 05/11/1977 | 02/08/2017 | 40 | 0 | 0 | Supero-esterno | 1 | 1 | 22/12/2016 | 0 | 1 | 51 | 1 | 1 | 3 | 1 | 0 | 0 | 0 | 78 | 0/1+ | ° | Antraciclina | Taxani | AC | Paclitaxel |
| 1878325 | 0 | 15/12/1963 | 26/09/2017 | 54 | 1 | 1 | Supero-interno | 1 | 1 | 19/01/2017 | 0 | 1 | 15 | 0 | 1 | 1c | 1 | 0 | 98 | 50 | 45 | 0/1+ | ° | Antraciclina | Taxani | EC | Paclitaxel |
| 1878563 | 1 | 30/06/1975 | 09/03/2017 | 42 | 0 | 0 | Supero-esterno | 1 | 1 | 27/09/2016 | 0 | 1 | 29 | 1 | 2 | 2 | 1 | 0 | 0 | 0 | 65 | 0/1+ | ° | Antraciclina | Taxani | AC | Docetaxel + Trastuzumab |
| 1893679 | 0 | 10/10/1964 | 15/06/2017 | 53 | 1 | 0 | Regione | 1 | 1 | 15/09/2016 | 1 | 0 | 51 | 0 | 1 | 3 | 0 | 0 | 0 | 0 | 80 | 0/1+ | ° | Antraciclina | Taxani | EC | Paclitaxel |
| 1896014 | 0 | 22/11/1967 | 07/07/2017 | 50 | 1 | 1 | Supero-esterno | 1 | 1 | 15/10/2016 | 0 | 0 | 39 | 0 | 1 | 2 | 0 | 0 | 90 | 90 | 60 | 2+ | 1 | Antraciclina | Taxani | EC | Paclitaxel + Trastuzumab |
| 1896287 | 1 | 17/08/1971 | 25/07/2017 | 46 | 0 | 1 | Infero-esterno | 1 | 1 | 15/01/2017 | 0 | 0 | 27 | 0 | 1 | 2 | 0 | 0 | 95 | 10 | 55 | 0/1+ | ° | Antraciclina | Taxani | AC | Paclitaxel |
| 1897133 | 1 | 02/01/1967 | 20/07/2017 | 49 | 0 | 1 | Infero-esterno | 0 | 1 | 15/11/2016 | 1 | 0 | 38 | 0 | 1 | 2 | 1 | 0 | 95 | 60 | 48 | 3+ | ° | Antraciclina | Taxani | EC | Paclitaxel + Trastuzumab |
| 1902706 | 1 | 07/08/1973 | 19/09/2017 | 44 | 0 | 1 | Supero-esterno | 1 | 1 | 15/01/2016 | 0 | 0 | 30 | 1 | 1 | 2 | 0 | 0 | ° | ° | 35 | 0/1+ | ° | Antraciclina | Taxani | EC | Paclitaxel |
| 1909542 | 0 | 20/03/1989 | 24/11/2017 | 28 | 0 | 0 | Supero-esterno | 1 | 1 | ° | 0 | 1 | 40 | 0 | 1 | 2 | 0 | 0 | 90 | 90 | ° | 3+ | ° | Antraciclina | Taxani | EC | Paclitaxel + Trastuzumab |
| 1915358 | 0 | 18/10/1974 | 29/12/2017 | 43 | 0 | 0 | Supero-interno | 1 | 1 | 02/08/2017 | 0 | 1 | 28 | 0 | 1 | 2 | 0 | 0 | 90 | 60 | 30 | 0/1+ | ° | Antraciclina | Taxani | EC | Docetaxel |
| 1926977 | 0 | 30/03/1982 | 04/05/2018 | 35 | 0 | 1 | Supero-interno | 1 | 1 | ° | 0 | 1 | 51 | 0 | 1 | 3 | 0 | 0 | 0 | 0 | 65 | 0/1+ | ° | Antraciclina | Taxani | AC | Paclitaxel |

| | | | | | | | | | | | | | | | | | | | | | | | | | | | |
|---|---|---|---|---|---|---|---|---|---|---|---|---|---|---|---|---|---|---|---|---|---|---|---|---|---|---|---|
| 1934568 | 1 | 13/11/1959 | 12/03/2019 | 60 | 1 | 0 | Supero-esterno | 1 | 1 | 26/06/2018 | 0 | 0 | 35 | 1 | 1 | 2 | 1 | 0 | 80 | 0 | 35 | 2+ | 1 | Antraciclina | Taxani | AC | Docetaxel + Trastuzumab |
| 1938785 | 0 | 09/10/1952 | 30/08/2018 | 66 | 1 | 0 | Supero-esterno | 0 | 1 | 27/01/2018 | 1 | 0 | ° | 0 | 1 | 1c | 1 | 0 | 0 | 0 | 60 | 0/1+ | ° | Antraciclina | Taxani | EC | Paclitaxel |
| 1943649 | 1 | 30/04/1966 | 16/11/2018 | 52 | 1 | 1 | Regione | 1 | 1 | 15/05/2018 | 0 | 0 | 51 | 1 | 1 | 3 | 0 | 0 | 95 | 95 | 80 | 2+ | 1 | Antraciclina | Taxani | AC | Paclitaxel + Trastuzumab |
| 1953878 | 0 | 05/03/1962 | 28/02/2019 | 57 | 1 | 0 | Supero-esterno | 1 | 1 | 09/07/2018 | 1 | 1 | 85 | 1 | 2 | 3 | 1 | 0 | 0 | 0 | 50 | 0/1+ | ° | Antraciclina | Taxani | EC | Paclitaxel |
| 1958492 | 1 | 24/11/1967 | 05/11/2019 | 52 | 0 | 1 | Supero-esterno | 1 | 1 | 01/03/2019 | 1 | 1 | 38 | 1 | 2 | 2 | 1 | 0 | 80 | 10 | 70 | 2+ | 0 | Antraciclina | Taxani | AC | Docetaxel |
| 1960026 | 1 | 14/05/1977 | 19/03/2019 | 42 | 0 | 1 | Infero-esterno | 1 | 1 | 21/08/2018 | 0 | 1 | 30 | 0 | 1 | 2 | 0 | 0 | 95 | 95 | 25 | 0/1+ | ° | Antraciclina | Taxani | AC | Paclitaxel |
| 1963894 | 0 | 02/12/1974 | 19/04/2019 | 44 | 0 | 0 | Esterno | 1 | 1 | 19/09/2018 | 0 | 1 | ° | 0 | 1 | 1c | 0 | 0 | 90 | 40 | 35 | 3+ | ° | Antraciclina | Taxani | EC | Paclitaxel + Trastuzumab |
| 1970643 | 0 | 26/06/1958 | 21/06/2019 | 60 | 1 | 0 | Supero-esterno | 1 | 1 | 05/12/2018 | 1 | 0 | 15 | 0 | 1 | 1c | 1 | 0 | 45 | 0 | 70 | 3+ | ° | Antraciclina | Taxani | EC | Paclitaxel |
| 1971387 | 0 | 17/11/1973 | 28/06/2019 | 46 | 0 | 0 | Supero-esterno | 1 | 1 | 27/12/2018 | 0 | 1 | 70 | 0 | 1 | 3 | 0 | 0 | 95 | 90 | 30 | 0/1+ | ° | Antraciclina | Taxani | EC | Paclitaxel |
| 1973696 | 0 | 05/03/1970 | 19/07/2019 | 49 | 0 | 0 | Esterno | 1 | 1 | 05/12/2018 | 1 | 0 | 51 | 0 | 2 | 3 | 1 | 0 | 95 | 5 | 25 | 3+ | ° | Antraciclina | Taxani | EC | Paclitaxel + Trastuzumab |
| 1977465 | 0 | 10/08/1994 | 13/09/2019 | 25 | 1 | 0 | Supero-interno | 1 | 1 | 15/02/2019 | 0 | 1 | 40 | 0 | 1 | 2 | 0 | 0 | 0 | 0 | 90 | 0/1+ | ° | Antraciclina | Taxani | EC | Paclitaxel |
| 1993669 | 0 | 11/07/1978 | 07/02/2020 | 42 | 0 | 1 | Supero-esterno | 1 | 1 | 15/08/2019 | 0 | 1 | 30 | 0 | 1 | 2 | 0 | 0 | 20 | 0 | 75 | 0/1+ | ° | Antraciclina | Taxani | EC | Paclitaxel |
| 1996551 | 1 | 25/07/1967 | 13/03/2020 | 52 | 1 | 0 | Infero-interno | 1 | 1 | 15/07/2019 | 0 | 0 | 14 | 1 | 1 | 1c | 0 | 0 | 0 | 0 | 75 | 0/1+ | ° | Antraciclina | Taxani | EC | Docetaxel |
| 1996787 | 0 | 17/09/1976 | 10/03/2020 | 43 | 0 | 0 | Interno | 1 | 1 | 15/07/2019 | 1 | 1 | 37 | 1 | 2 | 2 | 1 | 0 | 0 | 0 | 30 | 2+ | 1 | Antraciclina | Taxani | EC | Paclitaxel + Trastuzumab |
| 5009242 | 0 | 30/10/1975 | 28/09/2017 | 42 | 0 | 1 | Infero-centrale | 1 | 1 | 14/04/2017 | 0 | 1 | 12 | 0 | 1 | 1c | 0 | 0 | 0 | 0 | 35 | 0/1+ | ° | Antraciclina | Taxani | AC | Paclitaxel |
| 5014426 | 1 | 25/08/1963 | 28/11/2017 | 54 | 1 | 1 | Supero-esterno | 1 | 1 | 18/04/2017 | 1 | 0 | 30 | 1 | 1 | 2 | 1 | 0 | 98 | 15 | 45 | 2+ | 1 | Antraciclina | Taxani | AC | Docetaxel + Trastuzumab |
| 5019231 | 0 | 29/10/1967 | 18/01/2018 | 50 | 1 | 1 | Supero-esterno | 1 | 1 | 26/05/2017 | 1 | 0 | 7 | 1 | 2 | 1b | 1 | 0 | 0 | 0 | 35 | 3+ | ° | Antraciclina | Taxani | AC | Docetaxel + Trastuzumab |
| 5019338 | 1 | 14/12/1980 | 30/06/2017 | 37 | 0 | 0 | Interno | 0 | 1 | 15/01/2017 | 0 | 1 | 38 | 0 | 1 | 2 | 1 | 0 | 2 | 0 | 75 | 2+ | 0 | Antraciclina | Taxani | EC | Paclitaxel |
| 5020081 | 1 | 08/01/1977 | 16/01/2018 | 40 | 0 | 0 | Supero-esterno | 1 | 1 | 22/06/2018 | 1 | 1 | 23 | 1 | 1 | 2 | 1 | 0 | 0 | 0 | 15 | 3+ | ° | Antraciclina | Taxani | AC | Docetaxel + Trastuzumab |
| 5022815 | 0 | 14/12/1974 | 15/12/2017 | 43 | 0 | 0 | Supero-centrale | 1 | 1 | 15/06/2017 | 0 | 1 | 19 | 0 | 1 | 1c | 1 | 0 | 90 | 90 | ° | 2+ | 0 | Antraciclina | Taxani | AC | Paclitaxel |
| 5029805 | 0 | 07/06/1968 | 07/03/2018 | 49 | 0 | 0 | Esterno | 1 | 1 | 24/07/2017 | 0 | 1 | 30 | 1 | 2 | 2 | 0 | 0 | 0 | 0 | 30 | 2+ | 0 | Antraciclina | Taxani | AC | Paclitaxel |
| 5035277 | 0 | 27/01/1966 | 12/04/2018 | 51 | 1 | 1 | Supero-esterno | 1 | 1 | 14/09/2018 | 0 | 0 | 25 | 1 | 1 | 2 | 1 | 0 | 0 | 0 | 50 | 3+ | ° | Antraciclina | Taxani | AC | Docetaxel + Trastuzumab |
| 5036576 | 1 | 08/04/1939 | 21/06/2018 | 78 | 1 | 1 | Supero-esterno | 0 | 1 | 28/09/2017 | 0 | 0 | 30 | 1 | 1 | 2 | 1 | 0 | 0 | 0 | 10 | 0/1+ | ° | Antraciclina | Taxani | AC | Paclitaxel + Trastuzumab |
| 5038332 | 0 | 10/10/1963 | 04/06/2018 | 54 | 1 | 1 | Supero-esterno | 0 | 1 | 09/10/2017 | 0 | 0 | 40 | 1 | 1 | 2 | 1 | 0 | 95 | 30 | 60 | 2+ | 1 | Antraciclina | Taxani | AC | Paclitaxel + Trastuzumab |
| 5038851 | 1 | 15/10/1983 | 25/05/2018 | 34 | 0 | 1 | Supero-esterno | 1 | 1 | 05/10/2017 | 0 | 0 | 53 | 1 | 2 | 3 | 1 | 0 | 85 | 95 | 25 | 3+ | ° | Antraciclina | Taxani | AC | Docetaxel + Trastuzumab |
| 5038919 | 0 | 06/06/1967 | 28/07/2018 | 51 | 1 | 1 | Supero-esterno | 1 | 1 | 19/09/2017 | 0 | 1 | 22 | 1 | 1 | 2 | 1 | 0 | 0 | 0 | 80 | 0/1+ | ° | Antraciclina | Taxani | AC | Docetaxel |
| 5041939 | 1 | 25/09/1959 | 24/05/2018 | 58 | 1 | 0 | Supero-esterno | 1 | 1 | 05/10/2017 | 0 | 0 | 33 | 1 | 1 | 2 | 0 | 0 | 98 | 89 | 40 | 3+ | ° | Antraciclina | Taxani | AC | Docetaxel + Trastuzumab |
| 5042802 | 0 | 28/03/1963 | 26/06/2018 | 54 | 1 | 0 | Supero-esterno | 0 | 1 | 06/11/2018 | 0 | 0 | 23 | 1 | 2 | 2 | 0 | 0 | 0 | 0 | 50 | 0/1+ | ° | Antraciclina | Taxani | AC | Paclitaxel |
| 5045817 | 1 | 05/09/1966 | 10/07/2018 | 52 | 1 | 0 | Supero-interno | 1 | 1 | 17/11/2017 | 0 | 0 | 35 | 1 | 2 | 2 | 1 | 0 | 95 | 90 | 20 | 2+ | 0 | Antraciclina | Taxani | AC | Docetaxel |
| 5047215 | 1 | 17/07/1982 | 17/07/2018 | 36 | 0 | 0 | Supero-esterno | 0 | 1 | 28/11/2017 | 1 | 1 | 28 | 1 | 2 | 2 | 1 | 0 | 85 | 30 | 40 | 3+ | ° | Antraciclina | Taxani | AC | Docetaxel + Trastuzumab |
| 5047231 | 1 | 02/02/1961 | 17/12/2019 | 58 | 1 | 0 | Infero-esterno | 1 | 1 | 30/04/2019 | 1 | 1 | 30 | 1 | 1 | 2 | 1 | 0 | 0 | 40 | 65 | 3+ | ° | Antraciclina | Taxani | AC | Docetaxel + Trastuzumab |
| 5048852 | 1 | 07/02/1956 | 21/05/2018 | 62 | 1 | 0 | Supero-esterno | 1 | 1 | 27/11/2017 | 0 | 0 | 21 | 1 | 1 | 2 | 0 | 0 | 0 | 0 | 15 | 3+ | ° | Antraciclina | Taxani | AC | Docetaxel + Trastuzumab |
| 5053135 | 0 | 28/09/1962 | 12/06/2018 | 56 | 1 | 1 | Supero-esterno | 1 | 1 | 21/12/2017 | 0 | 0 | 21 | 1 | 2 | 2 | 0 | 0 | 0 | 0 | 25 | 2+ | 0 | Antraciclina | Taxani | EC | Paclitaxel |
| 5053548 | 0 | 07/01/1977 | 02/08/2018 | 41 | 0 | 1 | Supero-esterno | 1 | 1 | 15/12/2017 | 1 | 0 | 30 | 0 | 1 | 2 | 1 | 0 | 99 | 80 | 15 | 0/1+ | ° | Antraciclina | Taxani | AC | Docetaxel |
| 5053852 | 1 | 19/07/1974 | 24/07/2018 | 44 | 0 | 1 | Esterno | 1 | 1 | 15/12/2017 | 1 | 0 | ° | 1 | 1 | 2 | 1 | 0 | 90 | 30 | 30 | 0/1+ | ° | Antraciclina | Taxani | AC | Paclitaxel |
| 5054865 | 0 | 17/02/1972 | 08/01/2018 | 45 | 1 | 1 | Infero-interno | 1 | 1 | 15/05/2017 | 0 | 0 | 21 | 0 | 1 | 2 | 0 | 0 | 0 | 0 | 80 | 0/1+ | ° | Antraciclina | Taxani | EC | Paclitaxel |
| 5054890 | 1 | 13/03/1971 | 10/09/2018 | 47 | 0 | 1 | Infero-esterno | 1 | 1 | 08/01/2018 | 0 | 0 | 37 | 1 | 1 | 2 | 0 | 0 | 0 | 0 | 65 | 0/1+ | ° | Antraciclina | Taxani | AC | Paclitaxel |
| 5057607 | 0 | 09/01/1944 | 01/06/2018 | 74 | 1 | 0 | Regione | 1 | 1 | 16/01/2018 | 0 | 0 | 18 | 1 | 2 | 1c | 1 | 0 | 95 | 80 | 30 | 0/1+ | ° | Antraciclina | ° | EC | ° |
| 5059216 | 1 | 06/11/1961 | 04/09/2018 | 57 | 1 | 1 | Infero-esterno | 1 | 1 | 17/01/2018 | 1 | 1 | 30 | 1 | 1 | 2 | 1 | 0 | 98 | 80 | 28 | 2+ | 1 | Antraciclina | Taxani | AC | Docetaxel + Trastuzumab |
| 5059765 | 1 | 24/07/1966 | 12/09/2018 | 52 | 1 | 0 | Infero-esterno | 1 | 1 | 26/01/2018 | 0 | 0 | 39 | 0 | 1 | 2 | 0 | 0 | 15 | 0 | 70 | 0/1+ | ° | Antraciclina | Taxani | AC | Paclitaxel |
| 5061718 | 1 | 04/10/1947 | 25/07/2018 | 71 | 1 | 0 | Diffuso | 1 | 1 | 28/12/2017 | 0 | 0 | 70 | 0 | 1 | 4d | 0 | 0 | 95 | 90 | 60 | 0/1+ | ° | Antraciclina | Taxani | AC | Paclitaxel |
| 5063663 | 1 | 16/11/1987 | 24/09/2018 | 31 | 0 | 0 | Esterno | 0 | 1 | 09/02/2018 | 0 | 0 | 21 | 1 | 2 | 2 | 0 | 0 | 95 | 95 | 18 | 3+ | ° | Antraciclina | Taxani | AC | Docetaxel + Trastuzumab |

| | | | | | | | | | | | | | | | | | | | | | | | | | | | | | |
|---|---|---|---|---|---|---|---|---|---|---|---|---|---|---|---|---|---|---|---|---|---|---|---|---|---|---|---|---|---|
| 5064975 | 0 | 23/07/1965 | 24/07/2018 | 53 | 1 | 1 | Infero-interno | 1 | 1 | 08/02/2018 | 1 | 1 | 40 | 1 | 1 | 2 | 1 | 0 | 0 | 0 | 38 | 3+ | ° | Antraciclina | Taxani | AC | Paclitaxel + Trastuzumab |
| 5065875 | 1 | 05/04/1963 | 04/10/2018 | 55 | 0 | 1 | Supero-esterno | 1 | 1 | 22/02/2018 | 0 | 0 | 90 | 1 | 1 | 3 | 0 | 0 | 80 | 0 | 30 | 3+ | ° | Antraciclina | Taxani | AC | Docetaxel + Trastuzumab |
| 5067103 | 1 | 18/03/1972 | 10/07/2018 | 46 | 1 | 1 | Supero-esterno | 1 | 1 | 23/02/2018 | 0 | 1 | 18 | 1 | 1 | 1c | 1 | 0 | 0 | 0 | 12 | 3+ | ° | Antraciclina | Taxani | AC | Paclitaxel + Trastuzumab |
| 5067864 | 0 | 30/11/1975 | 18/09/2018 | 43 | 1 | 1 | Infero-interno | 1 | 1 | 07/02/2018 | 0 | 1 | 21 | 0 | 1 | 2 | 0 | 0 | 0 | 0 | 85 | 3+ | ° | Antraciclina | Taxani | EC | Docetaxel + Trastuzumab |
| 5068107 | 0 | 27/03/1975 | 06/11/2018 | 43 | 0 | 1 | Interno | 0 | 1 | 13/02/2018 | 0 | 0 | ° | 1 | 1 | 2 | 0 | 0 | 2 | 0 | 45 | 0/1+ | ° | Antraciclina | Taxani | AC | Paclitaxel |
| 5071555 | 0 | 11/03/1968 | 30/10/2018 | 50 | 1 | 0 | Esterno | 1 | 1 | 21/02/2018 | 0 | 1 | 23 | 0 | 1 | 2 | 0 | 0 | 90 | 90 | 70 | 0/1+ | ° | Antraciclina | Taxani | EC | Paclitaxel |
| 5074360 | 0 | 24/06/1973 | 09/05/2018 | 44 | 0 | 1 | Supero-esterno | 1 | 1 | 04/10/2017 | 1 | 1 | 21 | 0 | 1 | 2 | 1 | 0 | 0 | 0 | 40 | 0/1+ | ° | Antraciclina | Taxani | AC | Paclitaxel |
| 5078147 | 0 | 23/09/1977 | 29/11/2018 | 41 | 0 | 1 | Supero-esterno | 1 | 1 | 24/04/2018 | 0 | 1 | 40 | 1 | 2 | 2 | 1 | 0 | 0 | 0 | 30 | 0/1+ | ° | Antraciclina | Taxani | AC | Paclitaxel + Trastuzumab |
| 5080494 | 1 | 20/05/1980 | 11/12/2018 | 38 | 0 | 0 | Supero-esterno | 1 | 1 | 04/04/2018 | 1 | 1 | 38 | 1 | 1 | 2 | 1 | 0 | 10 | 0 | 60 | 0/1+ | ° | Antraciclina | Taxani | AC | Paclitaxel |
| 5094059 | 0 | 27/07/1983 | 28/03/2019 | 35 | 0 | 0 | Supero-esterno | 1 | 1 | 20/07/2019 | 0 | 1 | 21 | 1 | 2 | 2 | 0 | 0 | 0 | 0 | 25 | 0/1+ | ° | Antraciclina | Taxani | AC | Paclitaxel |
| 5097202 | 1 | 06/02/1975 | 13/02/2019 | 71 | 0 | 0 | Diffuso | 1 | 1 | 27/06/2018 | 0 | 1 | 98 | 0 | 1 | 3 | 1 | 0 | 0 | 0 | 60 | 3+ | ° | Antraciclina | Taxani | AC | Paclitaxel + Trastuzumab |
| 5098017 | 1 | 22/03/1962 | 12/02/2019 | 56 | 1 | 1 | Infero-esterno | 1 | 1 | 04/07/2018 | 0 | 0 | 40 | 1 | 2 | 2 | 1 | 0 | 90 | 10 | 20 | 3+ | ° | Antraciclina | Taxani | AC | Docetaxel + Trastuzumab |
| 5101777 | 1 | 31/08/1949 | 19/04/2019 | 69 | 1 | 0 | Supero-esterno | 1 | 1 | 18/08/2018 | 0 | 0 | 30 | 0 | 1 | 2 | 0 | 0 | 95 | 90 | 35 | 2+ | 1 | Antraciclina | Taxani | AC | Docetaxel + Trastuzumab |
| 5105866 | 0 | 06/12/1979 | 28/05/2019 | 39 | 0 | 0 | Supero-esterno | 0 | 1 | 20/09/2018 | 0 | 1 | 21 | 1 | 1 | 2 | 0 | 0 | 0 | 35 | 45 | 0/1+ | ° | Antraciclina | Taxani | EC | Paclitaxel |
| 5107095 | 1 | 12/10/1979 | 07/05/2019 | 39 | 0 | 0 | Infero-esterno | 1 | 1 | 03/08/2018 | 1 | 0 | 35 | 1 | 1 | 2 | 1 | 0 | 95 | 80 | 40 | 2+ | 1 | Antraciclina | Taxani | AC | Docetaxel + Trastuzumab |
| 5108869 | 0 | 03/01/1962 | 18/04/2019 | 57 | 1 | 1 | Supero-centrale | 1 | 1 | 18/09/2018 | 0 | 0 | 38 | 1 | 1 | 2 | 0 | 0 | 0 | 0 | 75 | 0/1+ | ° | Antraciclina | Taxani | AC | Paclitaxel |
| 5109655 | 1 | 15/08/1939 | 05/03/2019 | 79 | 1 | 1 | Supero-esterno | 1 | 1 | 12/10/2018 | 0 | 0 | 30 | 1 | 2 | 2 | 1 | 0 | 70 | 55 | 20 | 3+ | ° | Antraciclina | Taxani | AC | Paclitaxel + Trastuzumab |
| 5110896 | 1 | 08/02/1987 | 10/04/2019 | 31 | 0 | 0 | Supero-esterno | 0 | 1 | 24/09/2018 | 0 | 0 | 21 | 1 | 2 | 2 | 1 | 0 | 90 | 70 | 40 | 0/1+ | ° | Antraciclina | Taxani | AC | Docetaxel |
| 5114369 | 0 | 28/09/1974 | 14/12/2018 | 44 | 0 | 0 | Esterno | 0 | 1 | 15/05/2018 | 0 | 1 | 37 | 0 | 2 | 2 | 1 | 0 | 80 | 80 | 50 | 0/1+ | ° | Antraciclina | Taxani | EC | Paclitaxel |
| 5114686 | 0 | 23/09/1960 | 06/03/2019 | 59 | 1 | 1 | Supero-esterno | 1 | 1 | 12/10/2018 | 0 | 0 | 30 | 1 | 1 | 4 | 0 | 0 | 90 | 80 | 10 | 0/1+ | ° | Antraciclina | Taxani | AC | Docetaxel |
| 5118161 | 0 | 28/10/1977 | 13/11/2018 | 41 | 1 | 1 | Supero-esterno | 1 | 1 | 15/04/2018 | 1 | 1 | 60 | 0 | 2 | 3 | 1 | 0 | 0 | 0 | 90 | 0/1+ | ° | Antraciclina | Taxani | AC | Docetaxel |
| 5132247 | 1 | 25/11/1977 | 27/08/2019 | 42 | 0 | 0 | Supero-centrale | 1 | 1 | 31/12/2018 | 1 | 1 | 22 | 1 | 1 | 2 | 1 | 0 | 0 | 0 | 70 | 0/1+ | ° | Antraciclina | Taxani | AC | Paclitaxel |
| 5132283 | 0 | 31/05/1989 | 31/01/2019 | 29 | 0 | 0 | Supero-esterno | 1 | 1 | 15/11/2018 | 1 | 0 | ° | 1 | 1 | 2 | 1 | 0 | 0 | 0 | 80 | 0/1+ | ° | Antraciclina | ° | FEC | ° |
| 5132783 | 0 | 02/11/1961 | 18/09/2019 | 58 | 1 | 0 | Supero-esterno | 0 | 1 | 15/01/2019 | 0 | 0 | ° | 1 | 1 | 2 | 0 | 0 | 95 | 0 | 60 | 0/1+ | ° | Antraciclina | Taxani | AC | Docetaxel |
| 5132857 | 0 | 06/09/1962 | 01/02/2019 | 57 | 1 | 0 | Supero-esterno | 1 | 1 | 14/06/2018 | 1 | 1 | 70 | 0 | 1 | 3 | 1 | 0 | 95 | 80 | 30 | 0/1+ | ° | Antraciclina | Taxani | AC | Paclitaxel |
| 5133005 | 0 | 27/12/1973 | 22/02/2019 | 45 | 0 | 0 | Interno | 1 | 1 | 15/06/2018 | 0 | 1 | 45 | 0 | 1 | 2 | 0 | 0 | 0 | 0 | 90 | 0/1+ | ° | Antraciclina | Taxani | AC | Paclitaxel |
| 5133468 | 1 | 07/05/1979 | 29/08/2019 | 40 | 0 | 1 | Infero-centrale | 0 | 1 | 15/01/2019 | 0 | 0 | ° | 1 | 2 | 2 | 1 | 0 | 70 | 40 | 25 | 3+ | ° | Antraciclina | Taxani | AC | Docetaxel + Trastuzumab |
| 5135377 | 1 | 25/09/1976 | 02/08/2019 | 43 | 0 | 0 | Supero-esterno | 1 | 1 | ° | 0 | 0 | ° | 0 | 2 | 1c | 0 | 0 | ° | ° | ° | 3+ | ° | Antraciclina | Taxani | AC | Docetaxel + Trastuzumab |
| 5137424 | 1 | 30/08/1952 | 10/09/2019 | 67 | 1 | 1 | Supero-interno | 1 | 1 | 14/01/2019 | 1 | 1 | 30 | 1 | 1 | 2 | 1 | 0 | 95 | 0 | 30 | 0/1+ | ° | Antraciclina | Taxani | AC | Paclitaxel |
| 5139963 | 1 | 12/04/1967 | 03/12/2019 | 52 | 1 | 0 | Supero-centrale | 1 | 1 | 26/04/2019 | 1 | 1 | 100 | 1 | 1 | 3 | 1 | 0 | 0 | 0 | 60 | 2+ | 1 | Antraciclina | Taxani | AC | Docetaxel + Trastuzumab |
| 5143333 | 0 | 06/07/1972 | 30/10/2019 | 47 | 1 | 1 | Supero-esterno | 1 | 1 | 24/04/2019 | 0 | 1 | 21 | 1 | 2 | 2 | 1 | 0 | 70 | 20 | 15 | 3+ | ° | Antraciclina | Taxani | AC | Docetaxel + Trastuzumab |
| 5145254 | 0 | 20/07/1957 | 04/04/2019 | 61 | 1 | 1 | Supero-esterno | 1 | 1 | 28/08/2019 | 0 | 0 | 28 | 1 | 1 | 2 | 0 | 0 | 90 | 0 | 35 | 0/1+ | ° | Antraciclina | ° | AC | ° |
| 5155799 | 1 | 23/11/1961 | 15/11/2019 | 58 | 1 | 1 | Esterno | 1 | 1 | 12/04/2019 | 1 | 0 | 40 | 1 | 1 | 2 | 1 | 0 | 95 | 10 | 50 | 0/1+ | ° | Antraciclina | Taxani | AC | Docetaxel |
| 5159350 | 1 | 27/07/1978 | 22/01/2020 | 42 | 0 | 1 | Supero-esterno | 1 | 1 | 30/04/2019 | 0 | 1 | 30 | 1 | 2 | 2 | 1 | 0 | 0 | 0 | 45 | 0/1+ | ° | Antraciclina | Taxani | AC | Paclitaxel |
| 5159790 | 0 | 21/12/1974 | 02/10/2019 | 45 | 0 | 1 | Supero-esterno | 1 | 1 | 07/05/2019 | 1 | 1 | 26 | 1 | 1 | 2 | 1 | 0 | 90 | 20 | 80 | 0/1+ | ° | Antraciclina | ° | AC | ° |
| 5169428 | 1 | 04/04/1966 | 14/01/2020 | 54 | 0 | 1 | Supero-esterno | 0 | 1 | 28/05/2019 | 1 | 0 | 18 | 1 | 1 | 1c | 1 | 0 | 0 | 0 | 40 | 3+ | ° | Antraciclina | Taxani | AC | Docetaxel + Trastuzumab |
| 5175168 | 0 | 02/05/1970 | 08/01/2020 | 49 | 0 | 0 | Supero-interno | 1 | 1 | 03/07/2019 | 0 | 0 | 30 | 0 | 1 | 2 | 0 | 0 | 90 | 90 | 13 | 3+ | ° | Antraciclina | Taxani | EC | Docetaxel + Trastuzumab |
| 5177406 | 1 | 02/02/1951 | 05/03/2020 | 68 | 1 | 0 | Supero-esterno | 1 | 1 | 20/06/2019 | 1 | 1 | 25 | 1 | 1 | 2 | 1 | 0 | 50 | 0 | 70 | 3+ | ° | Antraciclina | Taxani | AC | Paclitaxel + Trastuzumab |
| 5180267 | 1 | 12/01/1986 | 29/11/2019 | 33 | 0 | 1 | Supero-interno | 0 | 1 | 31/07/2019 | 0 | 0 | ° | 1 | 1 | 2 | 0 | 0 | 20 | 20 | 25 | 0/1+ | ° | Antraciclina | ° | AC | ° |
| 5188079 | 1 | 27/06/1973 | 21/04/2020 | 46 | 0 | 1 | Prolungamento | 1 | 1 | 25/09/2019 | 1 | 1 | 15 | 1 | 1 | 1c | 1 | 0 | 0 | 0 | 65 | 3+ | ° | Antraciclina | Taxani | AC | Paclitaxel + Trastuzumab |
| 5218996 | 0 | 19/08/1960 | 16/01/2020 | 59 | 1 | 0 | Supero-centrale | 1 | 1 | 06/07/2019 | 1 | 1 | 48 | 0 | 2 | 2 | 1 | 0 | 99 | 99 | 30 | 3+ | ° | Antraciclina | Taxani | AC | Paclitaxel + Trastuzumab |
| 5224593 | 0 | 24/10/1972 | 14/02/2020 | 48 | 0 | 0 | Supero-esterno | 1 | 1 | 15/06/2019 | 1 | 1 | 40 | 0 | 1 | 2 | 1 | 0 | 0 | 0 | 80 | 0/1+ | ° | Antraciclina | Taxani | EC | Paclitaxel |

| | | | | | | | | | | | | | | | | | | | | | | | | | | | |
|---|---|---|---|---|---|---|---|---|---|---|---|---|---|---|---|---|---|---|---|---|---|---|---|---|---|---|---|
| 1939222 | 1 | 11/05/1959 | 07/05/2020 | 60 | 1 | 1 | Supero-esterno | 1 | 1 | 26/09/2019 | 0 | 1 | 25 | 1 | 1 | 2 | 1 | 0 | 10 | 0 | 80 | 3+ | ° | Antraciclina | Taxani | AC | Docetaxel + Trastuzumab |
| 5191926 | 1 | 29/04/1975 | 08/05/2020 | 44 | 0 | 0 | Supero-interno | 1 | 1 | 06/09/2019 | 0 | 0 | 22 | 1 | 1 | 2 | 1 | 0 | 1 | 1 | 20 | 0/1+ | ° | Antraciclina | Taxani | AC | Paclitaxel |
| 5237993 | 1 | 28/12/1977 | 12/05/2020 | 43 | 0 | 1 | Supero-esterno | 1 | 1 | ° | 0 | 1 | 40 | 0 | 2 | 2 | 0 | 0 | 75 | 35 | 35 | 3+ | ° | Antraciclina | Taxani | EC | Paclitaxel + Trastuzumab |
| 5187676 | 0 | 12/11/1949 | 14/05/2020 | 70 | 1 | 1 | Supero-esterno | 1 | 1 | 02/10/2019 | 1 | 1 | ° | 1 | 1 | 1 | 1 | 0 | 95 | 0 | 17 | 3+ | ° | Antraciclina | Taxani | AC | Docetaxel + Trastuzumab |
| 5202479 | 0 | 17/11/1965 | 20/05/2020 | 54 | 1 | 1 | Esterno | 1 | 1 | 03/10/2019 | 0 | 0 | 23 | 1 | 1 | 2 | 0 | 0 | 45 | 0 | 65 | 0/1+ | ° | Antraciclina | Taxani | AC | Docetaxel |
| 76454 | 1 | 12/02/1981 | 21/05/2020 | 38 | 0 | 1 | Infero-centrale | 1 | 1 | 09/10/2019 | 0 | 1 | 50 | 1 | 1 | 2 | 1 | 0 | 0 | 0 | 25 | 3+ | ° | Antraciclina | Taxani | AC | Docetaxel + Trastuzumab |
| 9050493 | 1 | 14/04/1957 | 22/05/2020 | 63 | 1 | 0 | Supero-esterno | 1 | 1 | 09/10/2019 | 0 | 1 | 45 | 1 | 1 | 2 | 1 | 0 | 0 | 0 | ° | 3+ | ° | Antraciclina | Taxani | AC | Paclitaxel + Trastuzumab |
| 2000372 | 0 | 27/08/1957 | 29/05/2020 | 62 | 1 | 1 | Infero-centrale | 1 | 1 | 02/10/2019 | 0 | 0 | 27 | 0 | 1 | 2 | 1 | 0 | 0 | 0 | 30 | 3+ | ° | Taxani | Trastuzumab | Paclitaxel | Trastuzumab |
| 2000374 | 0 | 29/03/1968 | 29/05/2020 | 52 | 1 | 0 | Infero-interno | 1 | 1 | 18/09/2019 | 0 | 1 | 23 | 0 | 1 | 2 | 0 | 0 | 0 | 0 | 80 | 3+ | ° | Antraciclina | Taxani | EC | Paclitaxel + Trastuzumab |
| 5201712 | 1 | 10/04/1976 | 04/06/2020 | 43 | 0 | 1 | Esterno | 1 | 1 | 28/10/2019 | 1 | 0 | 20 | 1 | 1 | 1c | 0 | 0 | 0 | 0 | 50 | 3+ | ° | Antraciclina | Taxani | AC | Docetaxel |
| 304887 | 1 | 31/10/1963 | 04/06/2020 | 56 | 1 | 0 | Esterno | 1 | 1 | 18/11/2019 | 1 | 0 | 18 | 1 | 1 | 1c | 1 | 0 | 0 | 0 | 80 | 0/1+ | ° | Antraciclina | Taxani | AC | Paclitaxel |
| 5195283 | 1 | 26/07/1974 | 10/06/2020 | 46 | 0 | 1 | Supero-esterno | 1 | 1 | 26/09/2019 | 0 | 0 | ° | 1 | 1 | 1c | 0 | 0 | 0 | 0 | 40 | 0/1+ | ° | Antraciclina | Taxani | AC | Paclitaxel |
| 5243417 | 0 | 29/07/1966 | 11/06/2020 | 54 | 1 | 0 | Supero-esterno | 1 | 1 | 15/10/2019 | 1 | 1 | 33 | 1 | 1 | 2 | 1 | 0 | 60 | 5 | 25 | 2+ | 1 | Antraciclina | Taxani | AC | Docetaxel |
| 5199269 | 1 | 23/09/1973 | 12/06/2020 | 46 | 1 | 1 | Supero-esterno | 1 | 1 | 11/10/2019 | 0 | 1 | 18 | 1 | 1 | 1c | 0 | 0 | 0 | 0 | 50 | 3+ | ° | Antraciclina | Taxani | AC | Docetaxel + Trastuzumab |
| 5210124 | 0 | 03/07/1977 | 16/06/2020 | 42 | 0 | 0 | Supero-esterno | 1 | 1 | 28/11/2019 | 0 | 1 | 25 | 1 | 1 | 2 | 0 | 0 | 90 | 90 | 40 | 3+ | ° | Antraciclina | Taxani | AC | Docetaxel + Trastuzumab |
| 5199431 | 1 | 06/09/1964 | 17/06/2020 | 55 | 0 | 0 | Supero-interno | 0 | 1 | 18/01/2019 | 0 | 1 | 25 | 1 | 1 | 2 | 1 | 0 | 0 | 0 | 40 | 0/1+ | ° | Antraciclina | Taxani | AC | Paclitaxel |
| 5049904 | 1 | 09/12/1957 | 18/06/2020 | 62 | 1 | 1 | Supero-esterno | 1 | 1 | 21/10/2019 | 1 | 1 | 70 | 1 | 2 | 3 | 1 | 0 | 0 | 0 | 30 | 0/1+ | ° | Antraciclina | Taxani | AC | Paclitaxel |
| 5212981 | 0 | 20/12/1972 | 26/06/2020 | 48 | 0 | 1 | Infero-esterno | 1 | 1 | 15/12/2019 | 1 | 1 | 20 | 0 | 2 | 1c | 1 | 0 | 80 | 50 | 10 | 2+ | 1 | Antraciclina | Taxani | EC | Paclitaxel + Trastuzumab |
| 1806161 | 1 | 21/02/1967 | 02/07/2020 | 52 | 0 | 0 | Supero-esterno | 1 | 1 | 27/11/2019 | 1 | 1 | 40 | 1 | 1 | 2 | 1 | 0 | 0 | 0 | 80 | 0/1+ | ° | Antraciclina | Taxani | AC | Paclitaxel |
| 5205232 | 1 | 23/11/1961 | 02/07/2020 | 58 | 1 | 1 | Supero-esterno | 1 | 1 | 30/10/2019 | 0 | 1 | 23 | 1 | 2 | 2 | 0 | 0 | 95 | 40 | 20 | 3+ | ° | Antraciclina | Taxani | EC | Paclitaxel + Trastuzumab |
| 5240620 | 0 | 27/11/1971 | 03/07/2020 | 48 | 0 | 1 | Regione | 1 | 1 | 22/11/2019 | 0 | 1 | 18 | 0 | 1 | 1c | 0 | 0 | 95 | 90 | 40 | 3+ | ° | Antraciclina | Taxani | EC | Paclitaxel + Trastuzumab |
| 5209840 | 0 | 02/08/1961 | 14/07/2020 | 58 | 0 | 0 | Infero-esterno | 1 | 1 | 25/10/2019 | 1 | 0 | ° | 1 | 1 | 1c | 1 | 0 | 90 | 0 | 30 | 3+ | ° | Antraciclina | Taxani | AC | Docetaxel + Trastuzumab |
| 5213412 | 1 | 30/08/1988 | 24/07/2020 | 32 | 0 | 1 | Esterno | 1 | 1 | 07/12/2019 | 1 | 1 | ° | 1 | 1 | 3 | 0 | 0 | 0 | 0 | 80 | 0/1+ | ° | Antraciclina | Taxani | AC | Paclitaxel |
| 5218246 | 0 | 22/05/1975 | 29/07/2020 | 45 | 0 | 1 | Infero-esterno | 1 | 1 | 14/01/2020 | 1 | 0 | 15 | 1 | 1 | 1c | 0 | 0 | 0 | 0 | 60 | 0/1+ | ° | Antraciclina | Taxani | AC | Paclitaxel |
| 5217785 | 1 | 20/12/1988 | 04/08/2020 | 32 | 0 | 0 | Supero-interno | 1 | 1 | 26/11/2019 | 1 | 0 | ° | 1 | 1 | 1c | 0 | 0 | 15 | 0 | 90 | 0/1+ | ° | Antraciclina | Taxani | AC | Paclitaxel |
| 5207576 | 1 | 03/12/1988 | 04/08/2020 | 31 | 0 | 0 | Infero-interno | 1 | 1 | 31/10/2019 | 1 | 1 | 16 | 1 | 1 | 1c | 1 | 1 | 0 | 0 | 35 | 0/1+ | ° | Antraciclina | Taxani | AC | Paclitaxel |
| 1640401 | 1 | 22/05/1981 | 07/08/2020 | 39 | 0 | 1 | Supero-centrale | 1 | 1 | 20/12/2019 | 1 | 1 | 100 | 1 | 1 | 3 | 1 | 0 | 70 | 70 | 25 | 3+ | ° | Antraciclina | Taxani | AC | Docetaxel + Trastuzumab |
| 5249624 | 1 | 24/03/1983 | 13/08/2020 | 37 | 0 | 1 | Supero-esterno | 1 | 1 | 09/12/2019 | 0 | 0 | 23 | 0 | 1 | 2 | 0 | 1 | 1 | 1 | 70 | 0/1+ | ° | Antraciclina | Taxani | EC | Paclitaxel |
| 2005100 | 0 | 24/05/1955 | 13/08/2020 | 65 | 1 | 0 | Diffuso | 1 | 1 | 15/02/2020 | 1 | 1 | ° | 1 | 2 | 3 | 1 | 0 | 100 | 40 | 60 | 3+ | ° | Antraciclina | Taxani | AC | Paclitaxel + Trastuzumab e |
| 5217440 | 1 | 15/05/1975 | 19/08/2020 | 45 | 0 | 0 | Esterno | 1 | 1 | 24/12/2019 | 0 | 1 | 40 | 1 | 2 | 2 | 1 | 0 | 30 | 0 | 85 | 0/1+ | ° | Antraciclina | Taxani | AC | Paclitaxel |
| 373519 | 1 | 22/08/1955 | 20/08/2020 | 65 | 1 | 0 | Supero-esterno | 1 | 1 | 15/01/2020 | 1 | 0 | 22 | 0 | 2 | 2 | 1 | 0 | 2 | 2 | 10 | 3+ | ° | Antraciclina | Taxani | AC | Paclitaxel + Trastuzumab |
| 5222414 | 0 | 18/06/1968 | 03/09/2020 | 52 | 1 | 0 | Interno | 1 | 1 | 20/01/2020 | 1 | 1 | 18 | 1 | 1 | 1c | 1 | 0 | 0 | 0 | 40 | 3+ | ° | Antraciclina | Taxani | AC | Paclitaxel + Trastuzumab |
| 2006615 | 0 | 13/05/1947 | 04/09/2020 | 73 | 1 | 0 | Supero-esterno | 1 | 1 | ° | 1 | 1 | 22 | 1 | 1 | 2 | 1 | 0 | 90 | 80 | 20 | 2+ | 1 | Antraciclina | Taxani | AC | Paclitaxel + Trastuzumab |
| 5233332 | 1 | 27/01/1967 | 11/09/2020 | 53 | 1 | 0 | Supero-esterno | 1 | 1 | 10/12/2019 | 1 | 1 | 34 | 1 | 1 | 2 | 0 | 1 | 95 | 0 | 30 | 3+ | ° | Antraciclina | Taxani | AC | Paclitaxel + Trastuzumab |
| 2007434 | 0 | 09/09/1960 | 18/09/2020 | 60 | 1 | 0 | Supero-esterno | 1 | 1 | ° | 1 | 1 | 36 | 1 | 1 | 2 | 1 | 0 | 1 | 70 | 70 | 0/1+ | ° | Antraciclina | Taxani | EC | Paclitaxel |
| 5219509 | 0 | 05/03/1977 | 22/09/2020 | 43 | 0 | 0 | Supero-esterno | 1 | 1 | 04/02/2020 | 1 | 1 | 40 | 1 | 2 | 2 | 1 | 1 | 90 | 35 | 25 | 2+ | 0 | Antraciclina | Taxani | AC | Paclitaxel |
| 5236142 | 0 | 23/02/1970 | 13/10/2020 | 50 | 1 | 1 | Supero-esterno | 1 | 1 | 06/03/2020 | 0 | 1 | 16 | 1 | 1 | 1c | 0 | 0 | 0 | 0 | 90 | 3+ | ° | Antraciclina | Taxani | EC | Paclitaxel + Trastuzumab |
| 5233487 | 1 | 05/12/1987 | 13/10/2020 | 33 | 0 | 0 | Supero-esterno | 1 | 1 | 21/02/2020 | 1 | 1 | ° | 1 | 1 | 1c | 1 | 0 | 90 | 80 | 35 | 0/1+ | ° | Antraciclina | Taxani | AC | Docetaxel + Trastuzumab |
| 2010591 | 0 | 14/03/1991 | 23/10/2020 | 29 | 0 | 0 | Supero-esterno | 0 | 1 | 15/04/2020 | 0 | 1 | 26 | 1 | 2 | 2 | 1 | 0 | 95 | 95 | 11 | 2+ | 0 | Antraciclina | Taxani | EC | Paclitaxel |
| 5232026 | 0 | 01/05/1963 | 27/10/2020 | 57 | 1 | 0 | Supero-esterno | 1 | 1 | 09/03/2020 | 1 | 1 | ° | 1 | 1 | 1c | 1 | 0 | 0 | 0 | 30 | 0/1+ | ° | Antraciclina | Taxani | AC | Paclitaxel |
| 1898141 | 0 | 01/07/1982 | 10/11/2020 | 38 | 0 | 0 | Supero-esterno | 0 | 1 | 12/03/2020 | 0 | 0 | ° | 1 | 1 | 1c | 1 | 0 | 0 | 0 | 70 | 0/1+ | ° | Antraciclina | Taxani | AC | Paclitaxel |
| 5254878 | 1 | 10/08/1975 | 03/12/2020 | 45 | 0 | 0 | Infero-esterno | 1 | 1 | 26/05/2020 | 0 | 1 | 24 | 1 | 1 | 2 | 0 | 0 | 0 | 0 | 30 | 0/1+ | ° | Antraciclina | Taxani | AC | Paclitaxel |

| | | | | | | | | | | | | | | | | | | | | | | | | | | | |
|---|---|---|---|---|---|---|---|---|---|---|---|---|---|---|---|---|---|---|---|---|---|---|---|---|---|---|---|
| 5261572 | 1 | 28/07/1966 | 10/12/2020 | 54 | 1 | 0 | Supero-esterno | 1 | 1 | 25/08/2020 | 0 | 1 | 30 | 0 | 1 | 2 | 0 | 0 | 70 | 0 | 40 | 3+ | ° | Antraciclina | Taxani | AC | Paclitaxel + Trastuzumab |
| 5307773 | 1 | 26/03/1957 | 11/12/2020 | 63 | 1 | 1 | Supero-esterno | 1 | 1 | ° | 0 | 1 | 45 | 0 | 1 | 2 | 0 | 0 | 0 | 0 | 50 | 3+ | ° | Antraciclina | Taxani | EC | Paclitaxel + Trastuzumab |
| 5239383 | 1 | 19/01/1964 | 17/12/2020 | 56 | 1 | 0 | Esterno | 1 | 1 | 25/05/2020 | 0 | 0 | 25 | 1 | 2 | 2 | 0 | 0 | 0 | 0 | 37 | 3+ | ° | Antraciclina | Taxani | AC | Docetaxel + Trastuzumab |
| 5239087 | 1 | 18/12/1960 | 22/12/2020 | 60 | 1 | 0 | Supero-esterno | 1 | 1 | 19/05/2020 | 1 | 1 | 50 | 1 | 2 | 2 | 1 | 0 | 90 | 5 | 35 | 0/1+ | ° | Antraciclina | Taxani | AC | Docetaxel |
| 5308845 | 1 | 16/04/1975 | 29/12/2020 | 45 | 1 | 0 | Supero-esterno | 1 | 1 | 15/02/2020 | 1 | 0 | 69 | 1 | 2 | 4 | 1 | 0 | 99 | 90 | 55 | 0/1+ | ° | Antraciclina | Taxani | AC | Paclitaxel |
| 9523966 | 1 | 04/02/1953 | 05/01/2021 | 67 | 1 | 1 | Diffuso | 1 | 1 | 19/08/2020 | 1 | 0 | ° | 1 | 2 | 4 | 1 | 0 | 95 | 95 | 35 | 2+ | 0 | Antraciclina | Taxani | AC | Docetaxel |
| 2017005 | 1 | 09/06/1957 | 02/02/2021 | 64 | 1 | 1 | Supero-esterno | 1 | 1 | ° | 0 | 1 | 50 | 0 | 1 | 2 | 1 | 0 | 0 | 0 | 75 | 0/1+ | ° | Antraciclina | Taxani | AC | Paclitaxel |
| 5250432 | 1 | 04/03/1962 | 04/02/2021 | 58 | 1 | 0 | Infero-esterno | 1 | 1 | 05/06/2020 | 0 | 1 | 20 | 1 | 1 | 1c | 0 | 0 | 0 | 0 | 80 | 0/1+ | ° | Antraciclina | Taxani | AC | Paclitaxel |
| 5254757 | 0 | 03/11/1979 | 09/02/2021 | 41 | 0 | 1 | Supero-esterno | 1 | 1 | 13/07/2020 | 0 | 1 | 35 | 1 | 1 | 2 | 1 | 0 | 80 | 80 | 35 | 3+ | ° | Antraciclina | Taxani | AC | Docetaxel + Trastuzumab |
| 5248314 | 1 | 29/01/1948 | 16/02/2021 | 72 | 1 | 1 | Supero-esterno | 1 | 1 | 06/07/2020 | 1 | 1 | ° | 1 | 1 | 2 | 1 | 0 | 0 | 0 | 35 | 3+ | ° | Antraciclina | Taxani | EC | Docetaxel + Trastuzumab |
| 2004032 | 1 | 12/10/1963 | 18/02/2021 | 57 | 1 | 0 | Supero-interno | 0 | 1 | 24/07/2021 | 0 | 1 | 28 | 1 | 2 | 2 | 0 | 0 | 0 | 0 | 60 | 0/1+ | ° | Antraciclina | Taxani | AC | Paclitaxel |
| 5325228 | 0 | 13/09/1975 | 18/02/2021 | 45 | 0 | 1 | Esterno | 1 | 1 | 06/07/2020 | 1 | 0 | 30 | 1 | 2 | 2 | 1 | 0 | 95 | 60 | 12 | 0/1+ | ° | Antraciclina | Taxani | EC | Paclitaxel |
| 5252909 | 1 | 18/09/1974 | 02/03/2021 | 46 | 0 | 0 | Ascella | 1 | 1 | 13/07/2020 | 1 | 1 | 27 | 1 | 1 | 2 | 1 | 0 | 2 | 2 | 70 | 0/1+ | ° | Antraciclina | Taxani | AC | Paclitaxel |
| 5258035 | 1 | 03/08/1969 | 02/03/2021 | 51 | 1 | 0 | Supero-esterno | 1 | 1 | 31/08/2020 | 0 | 1 | 35 | 1 | 2 | 2 | 1 | 0 | 0 | 0 | 40 | 3+ | ° | Antraciclina | Taxani | AC | Docetaxel + Trastuzumab |
| 5316574 | 1 | 09/09/1978 | 04/03/2021 | 43 | 0 | 1 | Infero-interno | 1 | 1 | ° | 0 | 1 | 30 | 0 | 1 | 2 | 0 | 0 | 0 | 0 | 45 | 3+ | ° | Antraciclina | Taxani | AC | Paclitaxel + Trastuzumab |
| 5272258 | 0 | 22/01/1971 | 09/03/2021 | 49 | 0 | 1 | Supero-esterno | 1 | 1 | 30/07/2020 | 0 | 1 | 30 | 0 | 1 | 2 | 0 | 0 | 60 | 50 | 25 | 3+ | ° | Antraciclina | Taxani | AC | Paclitaxel + Trastuzumab |
| 5262847 | 0 | 30/04/1964 | 16/03/2021 | 56 | 1 | 0 | Supero-esterno | 1 | 1 | 21/08/2022 | 1 | 1 | 46 | 1 | 1 | 2 | 0 | 0 | 0 | 0 | 35 | 3+ | ° | Antraciclina | Taxani | AC | Docetaxel + Trastuzumab + |
| 5267705 | 0 | 18/02/1962 | 23/03/2021 | 58 | 1 | 1 | Supero-interno | 0 | 1 | 11/09/2020 | 0 | 1 | 25 | 1 | 1 | 2 | 0 | 0 | 0 | 35 | 20 | 3+ | ° | Antraciclina | Taxani | AC | Paclitaxel + Trastuzumab |
| 5224987 | 1 | 24/03/1991 | 08/04/2021 | 29 | 0 | 0 | Supero-esterno | 0 | 1 | 24/01/2020 | 1 | 1 | 15 | 1 | 1 | 1c | 1 | 1 | 90 | 1 | 10 | 2+ | 0 | Inibitore cicline | ° | Ribociclib | ° |
| 2021775 | 1 | 10/05/1992 | 15/04/2021 | 28 | 0 | 1 | Infero-esterno | 1 | 1 | ° | 0 | 1 | ° | 1 | 2 | 2 | 0 | 0 | 95 | 30 | 60 | 3+ | ° | Antraciclina | Taxani | EC | Paclitaxel + Trastuzumab |
| 5279302 | 1 | 30/11/1974 | 20/04/2021 | 46 | 0 | 0 | Supero-central | 1 | 1 | 03/09/2020 | 1 | 1 | 22 | 1 | 1 | 2 | 1 | 0 | 98 | 98 | 80 | 0/1+ | ° | Antraciclina | Taxani | AC | Docetaxel |
| 5346671 | 0 | 19/04/1978 | 30/04/2021 | 42 | 0 | 0 | Supero-central | 0 | 1 | 30/09/2020 | 0 | 1 | 18 | 0 | 1 | 1c | 0 | 0 | 95 | 30 | 25 | 3+ | ° | Antraciclina | Taxani | EC | Paclitaxel + Trastuzumab |
| 5283660 | 0 | 22/05/1961 | 13/05/2021 | 59 | 1 | 1 | Supero-esterno | 1 | 1 | 14/10/2020 | 1 | 1 | 22 | 1 | 2 | 2 | 1 | 0 | 80 | 45 | 20 | 3+ | ° | Antraciclina | Taxani | AC | Docetaxel + Trastuzumab |
| 10537050 | 1 | 19/03/1983 | 14/05/2021 | 37 | 0 | 0 | Supero-esterno | 1 | 1 | 15/09/2020 | 0 | 1 | ° | 1 | 1 | 2 | 0 | 0 | 90 | 80 | 30 | 2+ | 1 | Antraciclina | Taxani | AC | Paclitaxel + Trastuzumab |
| 5273657 | 1 | 24/11/1952 | 27/05/2021 | 68 | 1 | 1 | Supero-esterno | 1 | 1 | 24/08/2020 | 0 | 1 | 25 | 1 | 2 | 2 | 0 | 0 | 100 | 95 | 10 | 3+ | ° | Antraciclina | Taxani | AC | Paclitaxel + Trastuzumab |
| 66228 | 1 | 06/09/1966 | 04/06/2021 | 54 | 1 | 1 | Supero-esterno | 1 | 1 | 01/10/2020 | 0 | 1 | 26 | 1 | 2 | 2 | 1 | 0 | 50 | 5 | 15 | 3+ | ° | Antraciclina | Taxani | AC | Docetaxel + Trastuzumab |
| 280300 | 1 | 01/02/1976 | 04/06/2021 | 44 | 0 | 0 | Supero-esterno | 1 | 1 | 14/10/2020 | 0 | 1 | 36 | 1 | 1 | 2 | 0 | 0 | 15 | 1 | 25 | 3+ | ° | Antraciclina | Taxani | AC | Docetaxel + Trastuzumab |
| 5359865 | 0 | 01/05/1971 | 11/06/2021 | 49 | 1 | 0 | Interno | 1 | 1 | 09/11/2020 | 1 | 1 | 25 | 0 | 2 | 2 | 1 | 0 | 85 | 75 | 8 | 2+ | 0 | Antraciclina | Taxani | EC | Paclitaxel |
| 231733 | 1 | 26/03/1966 | 24/06/2021 | 54 | 1 | 0 | Supero-central | 1 | 1 | 20/10/2020 | 1 | 1 | 26 | 1 | 2 | 2 | 1 | 0 | 90 | 80 | 60 | 3+ | ° | Antraciclina | Taxani | AC | Docetaxel + Trastuzumab |
| 5009789 | 1 | 03/06/1942 | 08/07/2021 | 79 | 1 | 1 | Supero-esterno | 1 | 1 | 05/01/2021 | 0 | 0 | 45 | 1 | 2 | 2 | 0 | 0 | 95 | 95 | 25 | 2+ | 1 | Taxani | Trastuzumab | Paclitaxel | Trastuzumab |
| 5377116 | 0 | 30/12/1966 | 30/07/2021 | 55 | 1 | 1 | Supero-esterno | 1 | 1 | 27/01/2021 | 1 | 1 | 32 | 0 | 1 | 2 | 1 | 0 | 90 | 25 | 40 | 0/1+ | ° | Antraciclina | Taxani | EC | Paclitaxel |
| 235854 | 1 | 29/01/1966 | 20/08/2021 | 54 | 1 | 1 | Supero-esterno | 1 | 1 | 11/11/2020 | 0 | 1 | 28 | 1 | 1 | 2 | 0 | 0 | 0 | 0 | 80 | 2+ | 0 | Antraciclina | Taxani | AC | Paclitaxel |
| 630360 | 1 | 18/08/1981 | 23/08/2021 | 39 | 0 | 0 | Esterno | 1 | 1 | 08/01/2021 | 1 | 1 | 30 | 1 | 1 | 2 | 1 | 0 | 0 | 0 | 30 | 3+ | ° | Antraciclina | Taxani | AC | Docetaxel + Trastuzumab |
| 5225649 | 1 | 03/07/1976 | 27/08/2021 | 44 | 1 | 1 | Supero-esterno | 1 | 1 | 16/10/2020 | 0 | 1 | 40 | 1 | 1 | 2 | 1 | 0 | 0 | 0 | 60 | 0/1+ | ° | Antraciclina | Taxani | AC | Paclitaxel |
| 5317531 | 1 | 27/10/1960 | 31/08/2021 | 61 | 1 | 1 | Supero-esterno | 1 | 1 | 05/12/2020 | 1 | 1 | 15 | 1 | 1 | 1c | 0 | 0 | 0 | 0 | 15 | 3+ | ° | Antraciclina | Taxani | AC | Docetaxel + Trastuzumab |
| 5319400 | 1 | 14/07/1964 | 31/08/2021 | 57 | 1 | 0 | Supero-esterno | 1 | 1 | 12/12/2020 | 0 | 1 | 15 | 1 | 1 | 1c | 0 | 0 | 90 | 90 | 18 | 3+ | ° | Antraciclina | Taxani | AC | Docetaxel + Trastuzumab |
| 5313408 | 0 | 04/03/1072 | 07/09/2021 | 49 | 0 | 1 | Supero-esterno | 1 | 1 | 22/12/2020 | 0 | 1 | ° | 1 | 2 | 1c | 0 | 0 | 15 | 1 | 75 | 0/1+ | ° | Antraciclina | Taxani | AC | Paclitaxel |
| 5320153 | 1 | 19/12/1979 | 09/09/2021 | 42 | 0 | 1 | Supero-central | 1 | 1 | 11/01/2021 | 0 | 1 | 20 | 1 | 1 | 1c | 0 | 0 | 10 | 0 | 12 | 3+ | ° | Antraciclina | Taxani | AC | Docetaxel + Trastuzumab |
| 408455 | 1 | 01/05/1954 | 10/09/2021 | 67 | 1 | 1 | Supero-esterno | 1 | 1 | 18/01/2021 | 0 | 1 | 22 | 1 | 2 | 2 | 0 | 0 | 0 | 0 | 30 | 3+ | ° | Antraciclina | Taxani | EC | Paclitaxel + Trastuzumab |
| 9800177 | 1 | 30/04/1979 | 10/09/2021 | 42 | 0 | 1 | Infero-central | 1 | 1 | 05/01/2021 | 0 | 1 | 35 | 1 | 1 | 2 | 0 | 0 | 0 | 0 | 25 | 0/1+ | ° | Antraciclina | Taxani | AC | Paclitaxel |
| 5317038 | 0 | 27/10/1965 | 17/09/2021 | 56 | 1 | 0 | Supero-esterno | 1 | 1 | ° | 1 | 1 | ° | 1 | 2 | 1c | 1 | 0 | 0 | 0 | 65 | 0/1+ | ° | Antraciclina | Taxani | EC | Paclitaxel |
| 5430987 | 0 | 28/02/1981 | 28/09/2021 | 40 | 1 | 0 | Supero-interno | 1 | 1 | ° | 0 | 1 | 30 | 0 | 1 | 1c | 0 | 0 | 0 | 0 | 25 | 3+ | ° | Antraciclina | Taxani | EC | Paclitaxel + Trastuzumab |

| | | | | | | | | | | | | | | | | | | | | | | | | | | | |
|---|---|---|---|---|---|---|---|---|---|---|---|---|---|---|---|---|---|---|---|---|---|---|---|---|---|---|---|
| 5125439 | 1 | 12/02/1971 | 13/07/2021 | 49 | 1 | 0 | Supero-esterno | 1 | 1 | 19/11/2020 | 1 | 1 | 50 | 1 | 2 | 2 | 1 | 0 | 90 | 60 | 25 | 3+ | ° | Antraciclina | Taxani | AC | Docetaxel + Trastuzumab |
| 5276049 | 1 | 18/03/1988 | 15/07/2021 | 33 | 0 | 0 | Supero-esterno | 1 | 1 | 26/09/2021 | 1 | 0 | 30 | 1 | 1 | 2 | 0 | 0 | 35 | 5 | 60 | 0/1+ | ° | Antraciclina | Taxani | AC | Docetaxel |
| 5308566 | 1 | 08/07/1970 | 20/07/2021 | 51 | 0 | 0 | Supero-central | 1 | 1 | ° | 1 | 1 | 34 | 1 | 2 | 2 | 1 | 0 | 0 | 0 | 60 | 3+ | ° | Antraciclina | Taxani | AC | Docetaxel |
| 5397710 | 0 | 29/06/1965 | 23/07/2021 | 56 | 1 | 1 | Supero-esterno | 1 | 1 | ° | 0 | 1 | ° | 1 | 1 | 2 | 0 | 0 | 95 | 95 | 15 | 3+ | ° | Antraciclina | Taxani | EC | Paclitaxel + Trastuzumab |
| 121560 | 1 | 05/01/1949 | 21/10/2021 | 72 | 1 | 1 | Infero-central | 1 | 1 | 23/02/2021 | 1 | 1 | 30 | 1 | 2 | 2 | 1 | 0 | 0 | 0 | 20 | 3+ | ° | Antraciclina | Taxani | EC | Docetaxel + Trastuzumab |
| 5301821 | 1 | 04/04/1947 | 21/10/2021 | 74 | 1 | 0 | Supero-esterno | 0 | 1 | 19/11/2021 | 1 | 0 | 40 | 0 | 1 | 2 | 1 | 0 | 30 | 0 | 40 | 3+ | ° | Antraciclina | Taxani | AC | Paclitaxel + Trastuzumab |
| 5451550 | 0 | 15/03/1978 | 11/11/2021 | 43 | 0 | 0 | Supero-esterno | 1 | 1 | 22/03/2021 | 1 | 1 | 20 | 1 | 1 | 1c | 1 | 0 | 0 | 0 | ° | 0/1+ | ° | Antraciclina | Taxani | AC | Paclitaxel |
| 5356256 | 1 | 03/02/1980 | 16/11/2021 | 41 | 0 | 1 | Supero-esterno | 1 | 1 | 26/04/2021 | 1 | 0 | 50 | 1 | 1 | 2 | 1 | 0 | 90 | 90 | 60 | 3+ | ° | Antraciclina | Taxani | AC | Paclitaxel + Trastuzumab |
| 5368337 | 1 | 19/03/1966 | 09/12/2021 | 55 | 1 | 1 | Infero-esterno | 0 | 1 | 18/05/2021 | 1 | 1 | 18 | 1 | 1 | 1c | 1 | 0 | 0 | 0 | 25 | 3+ | ° | Antraciclina | Taxani | AC | Paclitaxel + Trastuzumab |
| | | | | | | | | | | | | | | | | | | | | | | | | | | | |

| CICLI 1° | CICLI 2° | Tipo I intervento | BLS | Num LS | pN (sn) | DA | Tot linfonodi | Linfonodi non-sentinella metastatici | Istotipo | ILV | Dim post | ER % | PgR % | Ki67 % | HER2 | FISH | pT | pN | RT | CT adj | Bio | HT | Recidiva | Data recidiva | DFS | Tipo recidiva | tipo recidiva loco-regionale | trattamento recidiva loco-regionale |
|---|---|---|---|---|---|---|---|---|---|---|---|---|---|---|---|---|---|---|---|---|---|---|---|---|---|---|---|---|
| 4 | 4+4 | 1 | 1 | 1 | 0 (sn) | 0 | ° | ° | 1 | 0 | 0 | ° | ° | ° | ° | ° | is | 0 | 1 | 0 | 1 | 0 | 0 | ° | 43,7 | ° | ° | ° |
| 6 | ° | 1 | 1 | 1 | 0 (sn) | 0 | ° | ° | 1 | 0 | 9 | 95 | 40 | 10 | 0/1+ | ° | 1b | 0 | 1 | 0 | 0 | 1 | 0 | ° | 159,1 | ° | ° | ° |
| 3 | 3+1 | 2 | 1 | 1 | 0 (sn) | 0 | ° | ° | 1 | 0 | 30 | 0 | 0 | 80 | 3+ | ° | 2 | 0 | 1 | 0 | 1 | 0 | 1 | 06/10/2017 | 23,8 | 2 | ° | ° |
| 4 | 12+12 | 2 | 1 | 2 | 0 (sn) | 0 | ° | ° | 1 | 0 | 11 | 0 | 0 | 50 | 3+ | ° | 1c | 0 | 1 | 0 | 1 | 0 | 0 | ° | 65,9 | ° | ° | ° |
| 4 | 4+4 | 2 | 1 | 3 | 0 (sn) | 0 | ° | ° | 1 | 0 | 0 | ° | ° | ° | ° | ° | is | 0 | 0 | 0 | 1 | 0 | 0 | ° | 41,9 | ° | ° | ° |
| 4 | 12 | 1 | 1 | 1 | 0 (sn) | 0 | ° | ° | 1 | 0 | 27 | 0 | 0 | 80 | 0/1+ | ° | 2 | 0 | 1 | 0 | 0 | 0 | 1 | 18/04/2019 | 13,1 | 2 | ° | ° |
| 4 | 4+4 | 1 | 1 | 2 | Mi(sn) | 0 | ° | ° | 3 | 0 | 9 | 90 | 0 | 12 | 3+ | ° | 1b | 1Mi(sn) | 1 | 0 | 1 | 1 | 0 | ° | 85,0 | ° | ° | ° |
| 4 | 3 | 1 | 1 | 2 | 1(sn) | 1 | 18 | 2 | 1 | 1 | 26 | 90 | 40 | 10 | 0/1+ | ° | 2 | 1a | 1 | 0 | 0 | 1 | 0 | ° | 97,9 | ° | ° | ° |
| 4 | 12 | 2 | 1 | 1 | 0 (sn) | 0 | ° | ° | 1 | 1 | 8 | 0 | 0 | 10 | 0/1+ | ° | 1b | 0 | 1 | 0 | 0 | 0 | 0 | ° | 128,6 | ° | ° | ° |
| 4 | 12 | 1 | 1 | 1 | 1(sn) | 1 | 22 | 7 | 1 | 0 | 7 | 0 | 0 | 80 | 0/1+ | ° | 1b | 2a | 1 | 1 | 0 | 0 | 1 | 03/03/2021 | 12,3 | 2 | ° | ° |
| 6 | ° | 1 | 1 | 1 | 1(sn) | 1 | 26 | 9 | 1 | 0 | 21 | 70 | 40 | 1 | 2+ | 0 | 2 | 3a | 1 | 0 | 0 | 1 | 1 | 15/02/2014 | 60,3 | 3 | mammella | CT |
| 4 | 11 | 2 | 1 | 1 | 0 (sn) | 0 | ° | ° | 1 | 0 | 27 | 0 | 0 | 80 | 0/1+ | ° | 2 | 0 | 0 | 1 | 0 | 0 | 1 | 14/06/2019 | 15,0 | 2 | ° | ° |
| 4 | ° | 1 | 1 | 1 | 0 (sn) | 0 | ° | ° | 1 | 0 | 0 | ° | ° | ° | ° | ° | 0 | 0 | 1 | 0 | 0 | 1 | 0 | ° | 49,0 | ° | ° | ° |
| 4 | 12+12 | 1 | 1 | 1 | 0 (sn) | 0 | ° | ° | 1 | 0 | 0 | ° | ° | ° | ° | ° | 0 | 0 | 1 | 0 | 1 | 0 | 0 | ° | 26,8 | ° | ° | ° |
| 12 | ° | 2 | 1 | 1 | 0 (sn) | 0 | ° | ° | 1 | 1 | 60 | 0 | 0 | 40 | 3+ | ° | 3 | 0 | 0 | 0 | 1 | 0 | 0 | ° | 20,0 | ° | ° | ° |
| 4 | 12 | 2 | 1 | 1 | 0 (sn) | 0 | ° | ° | 1 | 0 | 12 | 0 | 0 | 85 | 0/1+ | ° | 1c | 0 | 0 | 0 | 0 | 0 | 0 | ° | 35,7 | ° | ° | ° |
| 4 | 12+12 | 1 | 1 | 1 | 0 (sn) | 0 | ° | ° | 1 | 0 | 0 | ° | ° | ° | ° | ° | 0 | 0 | 1 | 0 | 1 | 1 | 0 | ° | 102,9 | ° | ° | ° |
| 4 | 12+12 | 1 | 1 | 1 | 0 (sn) | 0 | ° | ° | 1 | 0 | 0 | ° | ° | ° | ° | ° | 0 | 0 | 1 | 0 | 1 | 1 | 0 | ° | 43,4 | ° | ° | ° |
| 6 | ° | 2 | 1 | 1 | 1(sn) | 1 | 15 | 0 | 1 | 0 | 23 | 90 | 90 | 7 | 0/1+ | ° | 2 | 1a | 1 | 0 | 0 | 1 | 1 | 19/07/2022 | 160,5 | 3 | cute e sottocute | CT, HT |
| 4 | 12 | 2 | 1 | 1 | 0 (sn) | 0 | ° | ° | 1 | 0 | 3 | 65 | 0 | 3 | 0/1+ | ° | 1a | 0 | 1 | 0 | 0 | 1 | 1 | 30/09/2022 | 46,2 | 2 | ° | ° |
| 6 | ° | 1 | 1 | 1 | 1(sn) | 1 | 17 | 0 | 1 | 0 | 16 | 0 | 0 | 25 | 0/1+ | ° | 1c | 1a | 1 | 0 | 0 | 0 | 0 | ° | 132,9 | ° | ° | ° |
| 3 | 3 | 1 | 1 | 1 | 0 (sn) | 0 | ° | ° | 2 | 0 | 40 | 90 | 20 | 20 | 0/1+ | ° | 2 | 0 | 1 | 0 | 0 | 1 | 0 | ° | 157,2 | ° | ° | ° |
| 5 | ° | 1 | 1 | 1 | 0 (sn) | 0 | ° | ° | 1 | 0 | 0 | ° | ° | ° | ° | ° | 1mic | 0 | 1 | 0 | 0 | 1 | 0 | ° | 169,4 | ° | ° | ° |
| 6 | ° | 1 | 1 | 1 | 0 (sn) | 0 | ° | ° | 1 | 0 | 16 | 95 | 95 | 10 | 0/1+ | ° | 1c | 0 | 1 | 0 | 0 | 1 | 0 | ° | 156,6 | ° | ° | ° |
| 4 | 4 | 1 | 1 | 1 | 0 (sn) | 0 | ° | ° | 1 | 0 | 0 | ° | ° | ° | ° | ° | 0 | 0 | 1 | 0 | 1 | 1 | 0 | ° | 167,8 | ° | ° | ° |
| 6 | ° | 1 | 1 | 3 | 1(sn) | 1 | 28 | 0 | 1 | 0 | 17 | 95 | 90 | 10 | 0/1+ | ° | 1c | 1a | 1 | 0 | 0 | 1 | 1 | 15/09/2016 | 85,5 | 2 | ° | ° |
| 4 | 12 | 2 | 1 | 1 | Mi(sn) | 0 | ° | ° | 1 | 0 | 0 | ° | ° | ° | ° | ° | 0 | 1Mi(sn) | 1 | 0 | 0 | 0 | 0 | ° | 46,0 | ° | ° | ° |
| 6 | ° | 1 | 1 | 1 | 1(sn) | 1 | 27 | 0 | 0 | 1 | 12 | 90 | 50 | 3 | 0/1+ | ° | 1c | 1a | 1 | 0 | 0 | 1 | 0 | ° | 159,0 | ° | ° | ° |
| 4 | 4 | 1 | 1 | 5 | 0 (sn) | 0 | ° | ° | 1 | 0 | 45 | 5 | 0 | 30 | 0/1+ | ° | 2 | 0 | 0 | 0 | 0 | 1 | 0 | ° | 61,5 | ° | ° | ° |
| 4 | ° | 1 | 1 | 1 | 0 (sn) | 0 | ° | ° | 1 | 0 | 0 | ° | ° | ° | ° | ° | is | 0 | 1 | 0 | 0 | 1 | 0 | ° | 158,5 | ° | ° | ° |
| 3 | 3 | 1 | 1 | 1 | 0 (sn) | 0 | ° | ° | 1 | 0 | 0 | ° | ° | ° | ° | ° | 0 | 0 | 1 | 0 | 0 | 1 | 0 | ° | 137,4 | ° | ° | ° |
| 4 | 4+4 | 2 | 1 | 2 | 1(sn) | 1 | 18 | 1 | 1 | 0 | 7 | 80 | 15 | 10 | 3+ | ° | 1b | 1a | 1 | 0 | 1 | 1 | 0 | ° | 46,9 | ° | ° | ° |
| 4 | 4+4 | 1 | 1 | 1 | 0 (sn) | 0 | ° | ° | 1 | 0 | 4 | 80 | 80 | 30 | 3+ | ° | 1a | 0 | 1 | 0 | 1 | 1 | 0 | ° | 51,9 | ° | ° | ° |
| 4 | 12+12 | 1 | 1 | 1 | 0 (sn) | 0 | ° | ° | 1 | 1 | 0 | ° | ° | ° | ° | ° | 0 | 0 | 1 | 0 | 1 | 0 | 0 | ° | 143,8 | ° | ° | ° |
| 4 | 4 | 1 | 1 | 2 | 0 (sn) | 0 | ° | ° | 1 | 0 | 0 | ° | ° | ° | ° | ° | 0 | 0 | 1 | 0 | 0 | 1 | 0 | ° | 39,5 | ° | ° | ° |
| 4 | ° | 2 | 1 | 1 | 0 (sn) | 0 | ° | ° | 1 | 0 | 8 | 95 | 10 | 20 | 0/1+ | ° | 1b | 0 | 1 | 0 | 0 | 1 | 0 | ° | 121,4 | ° | ° | ° |
| 4 | 4+4 | 2 | 1 | 1 | 0 (sn) | 0 | ° | ° | 1 | 0 | 6 | 85 | 75 | 8 | 2+ | 0 | 1b | 0 | 0 | 0 | 0 | 1 | 0 | ° | 34,6 | ° | ° | ° |
| 4 | 12 | 1 | 1 | 1 | 0 (sn) | 0 | ° | ° | 1 | 1 | 21 | 0 | 0 | 25 | 0/1+ | ° | 2 | 0 | 1 | 0 | 0 | 0 | 1 | 07/08/2018 | 30,1 | 2 | ° | ° |
| 4 | 12 | 2 | 1 | 2 | 0 (sn) | 0 | ° | ° | 1 | 0 | 0 | ° | ° | ° | ° | ° | 0 | 0 | 1 | 0 | 0 | 0 | 0 | ° | 75,4 | ° | ° | ° |

| | | | | | | | | | | | | | | | | | | | | | | | | | | | | |
|---|---|---|---|---|---|---|---|---|---|---|---|---|---|---|---|---|---|---|---|---|---|---|---|---|---|---|---|---|
| 4 | 4+4 | 1 | 1 | 1 | 0 (sn) | 0 | ° | ° | 1 | 0 | 0 | ° | ° | ° | ° | ° | 1mic | 0 | 1 | 0 | 1 | 0 | 0 | ° | 111,6 | ° | ° | ° |
| 4 | ° | 1 | 1 | 1 | 1(sn) | 1 | 28 | 1 | 1 | 1 | 15 | 90 | 1 | 5 | 0/1+ | ° | 1c | 1a | 1 | 1 | 0 | 1 | 0 | ° | 106,5 | ° | ° | ° |
| 4 | 4 | 1 | 1 | 1 | 0 (sn) | 0 | ° | ° | 1 | 0 | 6 | 0 | 0 | 70 | 0/1+ | ° | 1b | 0 | 1 | 0 | 0 | 0 | 0 | ° | 109,2 | ° | ° | ° |
| 3 | ° | 2 | 1 | 1 | 0 (sn) | 0 | ° | ° | 1 | 1 | 11 | 0 | 0 | 35 | 0/1+ | ° | 1c | 0 | 1 | 1 | 0 | 0 | 0 | ° | 10,1 | ° | ° | ° |
| 4 | 4 | 1 | 1 | 1 | 0 (sn) | 0 | ° | ° | 1 | 0 | 0 | ° | ° | ° | ° | ° | 0 | 0 | 1 | 0 | 0 | 1 | 0 | ° | 96,4 | ° | ° | ° |
| 4 | 12 | 1 | 1 | 1 | 0 (sn) | 0 | ° | ° | 1 | 0 | 0 | ° | ° | ° | ° | ° | 0 | 0 | 1 | 0 | 0 | 0 | 0 | ° | 57,9 | ° | ° | ° |
| 4 | 4+4 | 2 | 1 | 1 | 0 (sn) | 0 | ° | ° | 1 | 0 | 0 | ° | ° | ° | ° | ° | is | 0 | 1 | 0 | 1 | 0 | 0 | ° | 103,6 | ° | ° | ° |
| 4 | ° | 1 | 1 | 1 | 0 (sn) | 0 | ° | ° | 1 | 0 | 17 | 95 | 20 | 10 | 0/1+ | ° | 1c | 0 | 1 | 0 | 0 | 1 | 0 | ° | 104,9 | ° | ° | ° |
| 4 | 4 | 2 | 1 | 1 | 0 (sn) | 0 | ° | ° | 1 | 1 | 49 | 90 | 10 | 10 | 0/1+ | ° | 2 | 0 | 1 | 0 | 0 | 1 | 1 | 16/09/2021 | 50,6 | 2 | ° | ° |
| 4 | 4 | 1 | 1 | 4 | 0 (sn) | 0 | ° | ° | 1 | 0 | 6 | 90 | 70 | 5 | 0/1+ | ° | 1b | 0 | 1 | 0 | 0 | 1 | 0 | ° | 39,8 | ° | ° | ° |
| 4 | ° | 2 | 1 | 1 | 1(sn) | 1 | 11 | 1 | 1 | 1 | 11 | 90 | 60 | 15 | 0/1+ | ° | 1c | 1a | 0 | 0 | 0 | 1 | 0 | ° | 86,8 | ° | ° | ° |
| 4 | ° | 2 | 1 | 1 | 0 (sn) | 0 | ° | ° | 0 | 0 | 22 | 40 | 0 | 10 | 0/1+ | ° | 2 | 0 | 1 | 1 | 0 | 1 | 0 | ° | 83,4 | ° | ° | ° |
| 4 | 12 | 1 | 1 | 1 | 0 (sn) | 0 | ° | ° | 1 | 1 | 21 | 0 | 0 | 35 | 3+ | ° | 2 | 0 | 1 | 0 | 1 | 0 | 1 | 19/09/2017 | 35,2 | 3 | | |
| 4 | 12+12 | 2 | 1 | 4 | 0 (sn) | 0 | ° | ° | 1 | 0 | 4 | 85 | 0 | 15 | 3+ | ° | 1a | 0 | 0 | 0 | 1 | 1 | 0 | ° | 86,8 | ° | ° | ° |
| 4 | 8 | 1 | 1 | 2 | Mi(sn) | 0 | ° | ° | 1 | 1 | 27 | 90 | 90 | 12 | 0/1+ | ° | 2 | 1Mi(sn) | 1 | 0 | 0 | 1 | 0 | ° | 1,4 | ° | ° | ° |
| 2 | 12 | 1 | 1 | 2 | 1(sn) | 1 | 18 | 4 | 1 | 0 | 5 | 90 | 0 | 25 | 0/1+ | ° | 1a | 2a | 1 | 0 | 0 | 1 | 0 | ° | 97,9 | ° | ° | ° |
| 4 | 12 | 2 | 1 | 1 | 0 (sn) | 0 | ° | ° | 1 | 0 | 0 | ° | ° | ° | ° | ° | 0 | 0 | 1 | 0 | 0 | 0 | 0 | ° | 103,1 | ° | ° | ° |
| 4 | 4 | 1 | 1 | 2 | 0 (sn) | 0 | ° | ° | 1 | 0 | 14 | 0 | 0 | 55 | 0/1+ | ° | 1c | 0 | 1 | 0 | 0 | 0 | 0 | ° | 85,4 | ° | ° | ° |
| 4 | 4+4 | 1 | 1 | 2 | 0 (sn) | 0 | ° | ° | 1 | 0 | 0 | ° | ° | ° | ° | ° | 0 | 0 | 1 | 0 | 1 | 1 | 0 | ° | 95,3 | ° | ° | ° |
| 4 | 4 | 2 | 1 | 1 | 0 (sn) | 0 | ° | ° | 1 | 0 | 22 | 0 | 0 | 70 | 0/1+ | ° | 2 | 0 | 1 | 1 | 0 | 0 | 1 | 26/11/2019 | 2,8 | 2 | ° | ° |
| 4 | ° | 1 | 1 | 1 | Mi(sn) | 1 | 15 | 0 | 0 | 0 | 6 | 90 | 5 | 15 | 0/1+ | ° | 1b | 1Mi(sn) | 1 | 0 | 0 | 1 | 0 | ° | 97,4 | ° | ° | ° |
| 4 | 12+12 | 1 | 1 | 2 | Mi(sn) | 0 | ° | ° | 1 | 0 | 3 | 99 | 99 | 15 | 0/1+ | ° | 1a | 1Mi(sn) | 1 | 0 | 1 | 1 | 0 | ° | 93,4 | ° | ° | ° |
| 4 | 4+4 | 1 | 1 | 3 | Mi(sn) | 0 | ° | ° | 1 | 0 | 12 | 85 | 0 | 10 | 3+ | ° | 1c | 1Mi(sn) | 1 | 0 | 1 | 1 | 0 | ° | 93,1 | ° | ° | ° |
| 4 | 4+4 | 2 | 1 | 1 | 0 (sn) | 0 | ° | ° | 1 | 0 | 0 | ° | ° | ° | ° | ° | is | 0 | 1 | 0 | 1 | 0 | 0 | ° | 79,1 | ° | ° | ° |
| 4 | 4 | 2 | 1 | 3 | 0 (sn) | 0 | ° | ° | 1 | 0 | 25 | 0 | 0 | 70 | 0/1+ | ° | 2 | 0 | 1 | 0 | 0 | 0 | 0 | ° | 93,1 | ° | ° | ° |
| 6 | ° | 2 | 1 | 2 | 1(sn) | 1 | 14 | 10 | 1 | 1 | 52 | 85 | 45 | 40 | 0/1+ | ° | 3 | 3a | 1 | 1 | 0 | 1 | 0 | ° | 39,9 | ° | ° | ° |
| 4 | 4 | 1 | 1 | 1 | 0 (sn) | 0 | ° | ° | 1 | 0 | 8 | 10 | 0 | 45 | 0/1+ | ° | 1b | 0 | 1 | 0 | 0 | 0 | 1 | 12/10/2016 | 16,3 | 3 | mammella | MAST |
| 4 | 9 | 1 | 1 | 2 | 1(sn) | 1 | 13 | 2 | 1 | 1 | 24 | 85 | 35 | 3 | 0/1+ | ° | 2 | 1a | 1 | 0 | 0 | 1 | 0 | ° | 70,8 | ° | ° | ° |
| 4 | 12 | 1 | 1 | 3 | 0 (sn) | 0 | ° | ° | 0 | 1 | 12 | 0 | 0 | 85 | 0/1+ | ° | 1c | i+(sn) | 1 | 0 | 0 | 0 | 0 | ° | 8,3 | ° | ° | ° |
| 4 | 4+4 | 1 | 1 | 3 | 0 (sn) | 0 | ° | ° | 1 | 1 | 0 | ° | ° | ° | ° | ° | 1mic | 0 | 1 | 0 | 1 | 0 | 0 | ° | 20,6 | ° | ° | ° |
| 4 | 7 | 2 | 1 | 1 | 0 (sn) | 0 | ° | ° | 1 | 0 | 55 | 0 | 0 | 80 | 0/1+ | ° | 3 | 0 | 1 | 1 | 0 | 0 | 1 | 18/01/2017 | 18,2 | 2 | ° | ° |
| 4 | 12 | 1 | 1 | 1 | Mi(sn) | 1 | 22 | 3 | 1 | 0 | 0 | ° | ° | ° | ° | ° | is | 2a | 1 | 0 | 0 | 0 | 0 | ° | 52,7 | ° | ° | ° |
| 3 | 12+12 | 1 | 1 | 2 | 0 (sn) | 0 | ° | ° | 1 | 0 | 0 | ° | ° | ° | ° | ° | 0 | 0 | 1 | 0 | 0 | 0 | 0 | ° | 0,5 | ° | ° | ° |
| 4 | 12 | 2 | 1 | 1 | 0 (sn) | 0 | ° | ° | 1 | 0 | 0 | ° | ° | ° | ° | ° | is | 0 | 0 | 0 | 0 | 0 | 0 | ° | 34,6 | ° | ° | ° |
| 3 | ° | 1 | 1 | 1 | 0 (sn) | 0 | ° | ° | 1 | 1 | 19 | 90 | 90 | 8 | 2+ | 0 | 1c | 0 | 1 | 0 | 0 | 1 | 0 | ° | 94,3 | ° | ° | ° |
| 4 | 12 | 2 | 1 | 1 | 0 (sn) | 0 | ° | ° | 1 | 0 | 0 | ° | ° | ° | ° | ° | 1mic | 0 | 1 | 0 | 1 | 1 | 0 | ° | 86,4 | ° | ° | ° |
| 4 | 12 | 2 | 1 | 1 | 0 (sn) | 0 | ° | ° | 1 | 1 | 22 | 0 | 0 | 50 | 0/1+ | ° | 2 | 0 | 0 | 0 | 0 | 0 | 0 | ° | 30,0 | ° | ° | ° |
| 4 | 4+4 | 2 | 1 | 3 | 0 (sn) | 0 | ° | ° | 1 | 0 | 0 | ° | ° | ° | ° | ° | is | 0 | 1 | 0 | 1 | 0 | 0 | ° | 83,6 | ° | ° | ° |
| 4 | 12 | 1 | 1 | 1 | 0 (sn) | 0 | ° | ° | 1 | 0 | 8 | 0 | 0 | ° | 0/1+ | ° | 1b | 0 | 1 | 0 | 0 | 0 | 1 | 07/11/2017 | 20,3 | 3 | mammella | MAST |
| 4 | 12 | 1 | 1 | 1 | 0 (sn) | 0 | ° | ° | 1 | 0 | 0 | ° | ° | ° | ° | ° | is | 0 | 1 | 0 | 0 | 0 | 0 | ° | 83,1 | ° | ° | ° |
| 4 | 4 | 1 | 1 | 1 | 1(sn) | 1 | 16 | 2 | 1 | 0 | 25 | 0 | 0 | 20 | 0/1+ | ° | 2 | 1a | 1 | 0 | 0 | 0 | 0 | ° | 82,9 | ° | ° | ° |
| 4 | 4+4 | 1 | 1 | 1 | 0 (sn) | 0 | ° | ° | 1 | 0 | 30 | 70 | 1 | 10 | 3+ | ° | 2 | 0 | 1 | 0 | 1 | 1 | 0 | ° | 83,3 | ° | ° | ° |

| | | | | | | | | | | | | | | | | | | | | | | | | | | | | |
|---|---|---|---|---|---|---|---|---|---|---|---|---|---|---|---|---|---|---|---|---|---|---|---|---|---|---|---|---|
| 4 | 12 | 2 | 1 | 1 | Mi(sn) | 0 | ° | ° | 1 | 0 | 30 | 90 | 10 | 5 | 0/1+ | ° | 2 | 1Mi(sn) | 0 | 0 | 0 | 1 | 0 | ° | 82,0 | ° | ° | ° |
| 4 | 12+12 | 1 | 1 | 3 | 0 (sn) | 0 | ° | ° | 1 | 0 | 4 | 80 | 0 | 5 | 0/1+ | ° | 1a | 0 | 1 | 0 | 1 | 1 | 0 | ° | 62,8 | ° | ° | ° |
| 4 | 12+12 | 1 | 1 | 1 | 0 (sn) | 0 | ° | ° | 1 | 0 | 0 | ° | ° | ° | ° | ° | is | 0 | 1 | 0 | 1 | 0 | 0 | ° | 69,4 | ° | ° | ° |
| 4 | 4 | 1 | 1 | 4 | 0 (sn) | 0 | ° | ° | 1 | 0 | 12 | 90 | 0 | 5 | 0/1+ | ° | 1c | 0 | 1 | 0 | 0 | 1 | 0 | ° | 86,7 | ° | ° | ° |
| 4 | 12 | 1 | 1 | 1 | 0 (sn) | 0 | ° | ° | 1 | 0 | 11 | 0 | 0 | 35 | 0/1+ | ° | 1c | 0 | 1 | 0 | 0 | 0 | 0 | ° | 76,2 | ° | ° | ° |
| 4 | 12 | 2 | 1 | 1 | 0 (sn) | 0 | ° | ° | 1 | 1 | 7 | 0 | 0 | 70 | 0/1+ | ° | 1b | 0 | 1 | 0 | 0 | 0 | 0 | ° | 76,1 | ° | ° | ° |
| 6 | 6 | 2 | 1 | 1 | 0 (sn) | 0 | ° | ° | 1 | 0 | 0 | ° | ° | ° | ° | ° | is | 0 | 1 | 0 | 1 | 0 | 0 | ° | 15,0 | ° | ° | ° |
| 4 | 12+12 | 1 | 1 | 1 | 0 (sn) | 0 | ° | ° | 1 | 0 | 17 | 0 | 0 | 10 | 3+ | ° | 1c | 0 | 1 | 0 | 1 | 0 | 0 | ° | 83,8 | ° | ° | ° |
| 4 | 4+4 | 2 | 1 | 2 | 0 (sn) | 0 | ° | ° | 0 | 0 | 47 | 30 | 0 | 3 | 2+ | 0 | 2 | 0 | 1 | 0 | 1 | 1 | 0 | ° | 73,4 | ° | ° | ° |
| 4 | 6 | 2 | 1 | 1 | 1(sn) | 1 | 9 | 3 | 1 | 1 | 14 | 90 | 0 | 12 | 0/1+ | ° | 1c | 2a | 1 | 0 | 0 | 1 | 0 | ° | 54,5 | ° | ° | ° |
| 4 | 12+12 | 1 | 1 | 3 | Mi(sn) | 1 | 3 | 0 | 1 | 0 | 0 | ° | ° | ° | ° | ° | 0 | 1Mi(sn) | 1 | 0 | 1 | 1 | 1 | 01/06/2020 | 48,4 | 2 | ° | ° |
| 4 | 4+4 | 2 | 1 | 1 | 0 (sn) | 0 | ° | ° | 1 | 0 | 0 | ° | ° | ° | ° | ° | is | 0 | 1 | 0 | 1 | 1 | 0 | ° | 71,8 | ° | ° | ° |
| 4 | 3+3 | 1 | 1 | 1 | 1(sn) | 1 | 8 | 4 | 1 | 0 | 8 | 90 | 90 | 5 | 0/1+ | ° | 1b | 2a | 1 | 0 | 0 | 1 | 0 | ° | 71,4 | ° | ° | ° |
| 4 | 4 | 1 | 1 | 2 | 1(sn) | 1 | 20 | 1 | 1 | 0 | 16 | 90 | 60 | 15 | 3+ | ° | 1c | 1a | 1 | 0 | 1 | 1 | 0 | ° | 7,8 | ° | ° | ° |
| 3 | 3 | 2 | 1 | 1 | 0 (sn) | 0 | ° | ° | 1 | 0 | 0 | ° | ° | ° | ° | ° | is | 0 | 1 | 0 | 0 | 1 | 0 | ° | 51,7 | ° | ° | ° |
| 4 | 12+12 | 2 | 1 | 2 | 0 (sn) | 0 | ° | ° | 1 | 0 | 0 | ° | ° | ° | ° | ° | is | 0 | 0 | 0 | 1 | 1 | 0 | ° | 55,6 | ° | ° | ° |
| 4 | 12+12 | 2 | 1 | 2 | 0 (sn) | 0 | ° | ° | 1 | 0 | 0 | ° | ° | ° | ° | ° | 0 | 0 | 1 | 0 | 1 | 1 | 0 | ° | 35,6 | ° | ° | ° |
| 4 | 12+12 | 1 | 1 | 1 | 0 (sn) | 0 | ° | ° | 1 | 0 | 0 | ° | ° | ° | ° | ° | 0 | 0 | 1 | 0 | 1 | 0 | 1 | 20/04/2017 | 8,9 | 1 | mammella | QUAD |
| 4 | 4+4 | 1 | 1 | 1 | 0 (sn) | 0 | ° | ° | 1 | 0 | 22 | 30 | 10 | 40 | 3+ | ° | 2 | 0 | 1 | 0 | 1 | 1 | 0 | ° | 58,1 | ° | ° | ° |
| 4 | 12 | 1 | 1 | 2 | 0 (sn) | 0 | ° | ° | 1 | 0 | 8 | 90 | 0 | 5 | 0/1+ | ° | 1b | 0 | 1 | 0 | 0 | 1 | 1 | 01/06/2021 | 57,2 | 1 | ascella | DA |
| 4 | 4+4 | 1 | 1 | 1 | 0 (sn) | 0 | ° | ° | 1 | 0 | 0 | ° | ° | ° | ° | ° | is | 0 | 1 | 0 | 1 | 0 | 0 | ° | 69,5 | ° | ° | ° |
| 4 | 4+4 | 1 | 1 | 1 | 0 (sn) | 0 | ° | ° | 1 | 1 | 8 | 60 | 0 | 5 | 3+ | ° | 1b | 0 | 1 | 0 | 1 | 1 | 0 | ° | 69,6 | ° | ° | ° |
| 4 | 4 | 1 | 1 | 3 | 1(sn) | 1 | 26 | 26 | 0 | 0 | 25 | 80 | 80 | 10 | 0/1+ | ° | 2 | 3a | 1 | 0 | 0 | 1 | 0 | ° | 68,7 | ° | ° | ° |
| 4 | 12+12 | 1 | 1 | 3 | 0 (sn) | 0 | ° | ° | 1 | 0 | 20 | 90 | 40 | 25 | 3+ | ° | 1c | 0 | 1 | 0 | 1 | 1 | 1 | 26/02/2019 | 25,4 | 1 | mammella | MAST |
| 4 | 12 | 1 | 1 | 2 | 1(sn) | 1 | 20 | 1 | 1 | 0 | 14 | 0 | 0 | 30 | 0/1+ | ° | 1c | 1a | 1 | 0 | 0 | 0 | 0 | ° | 65,0 | ° | ° | ° |
| 4 | 4+4 | 1 | 1 | 1 | Mi(sn) | 1 | 11 | 1 | 1 | 1 | 11 | 85 | 0 | 5 | 3+ | ° | 1c | 1a | 1 | 0 | 1 | 1 | 0 | ° | 73,5 | ° | ° | ° |
| 4 | 12 | 1 | 1 | 1 | 0 (sn) | 0 | ° | ° | 1 | 0 | 23 | 0 | 0 | 60 | 0/1+ | ° | 2 | 0 | 1 | 0 | 0 | 0 | 1 | 30/05/2019 | 22,6 | 2 | ° | ° |
| 12 | 12 | 1 | 1 | 1 | 0 (sn) | 0 | ° | ° | 1 | 1 | 48 | 90 | 70 | 10 | 2+ | 1 | 2 | 0 | 1 | 0 | 1 | 1 | 0 | ° | 57,0 | ° | ° | ° |
| 4 | 12 | 2 | 1 | 2 | 1(sn) | 1 | 21 | 6 | 1 | 1 | 12 | 90 | 1 | 10 | 2+ | 0 | 1c | 2a | 1 | 0 | 0 | 1 | 0 | ° | 72,8 | ° | ° | ° |
| 4 | 4 | 2 | 1 | 4 | Mi(sn) | 1 | 9 | 1 | 1 | 0 | 0 | ° | ° | ° | ° | ° | 1mic | 1Mi(sn) | 0 | 0 | 0 | 1 | 0 | ° | 65,0 | ° | ° | ° |
| 4 | 12 | 2 | 1 | 1 | 0 (sn) | 0 | ° | ° | 1 | 0 | 0 | ° | ° | ° | ° | ° | 0 | 0 | 0 | 0 | 0 | 0 | 0 | ° | 63,1 | ° | ° | ° |
| 4 | 12 | 1 | 1 | 1 | 0 (sn) | 0 | ° | ° | 1 | 0 | 18 | 0 | 0 | 60 | 0/1+ | ° | 1c | 0 | 1 | 0 | 0 | 0 | 0 | ° | 65,2 | ° | ° | ° |
| 4 | 12 | 1 | 1 | 3 | 1(sn) | 1 | 16 | 0 | 1 | 0 | 0 | ° | ° | ° | ° | ° | 1mic | 1a | 1 | 0 | 0 | 1 | 0 | ° | 63,3 | ° | ° | ° |
| 4 | 4+4 | 1 | 1 | 2 | 0 (sn) | 0 | ° | ° | 1 | 0 | 0 | ° | ° | ° | ° | ° | 0 | 0 | 1 | 0 | 0 | 0 | 0 | ° | 57,9 | ° | ° | ° |
| 4 | 12 | 2 | 1 | 2 | 0 (sn) | 0 | ° | ° | 1 | 0 | 17 | 0 | 0 | 80 | 0/1+ | ° | 1c | 0 | 0 | 0 | 0 | 0 | 0 | ° | 66,8 | ° | ° | ° |
| 4 | 12+12 | 2 | 1 | 1 | 0 (sn) | 0 | ° | ° | 1 | 0 | 8 | 90 | 1 | 5 | 0/1+ | ° | 1b | 0 | 0 | 0 | 1 | 1 | 0 | ° | 66,0 | ° | ° | ° |
| 4 | 9 | 1 | 1 | 2 | 0 (sn) | 0 | ° | ° | 2 | 0 | 5 | 75 | 3 | 5 | 0/1+ | ° | 1a | 0 | 1 | 1 | 0 | 1 | 0 | ° | 53,3 | ° | ° | ° |
| 4 | 12+12 | 1 | 1 | 1 | 0 (sn) | 0 | ° | ° | 1 | 0 | 15 | 90 | 30 | 5 | 3+ | ° | 1c | 0 | 1 | 0 | 1 | 1 | 0 | ° | 53,4 | ° | ° | ° |
| 4 | 12 | 2 | 1 | 1 | 0 (sn) | 0 | ° | ° | 1 | 0 | 9 | 50 | 0 | 5 | 0/1+ | ° | 1b | 0 | 0 | 0 | 0 | 1 | 1 | 01/12/2020 | 39,0 | 1 | ascella | DA |
| 4 | 12+12 | 1 | 1 | 2 | 0 (sn) | 0 | ° | ° | 1 | 0 | 9 | 90 | 0 | 20 | 3+ | ° | 1b | 0 | 1 | 0 | 1 | 1 | 0 | ° | 43,9 | ° | ° | ° |
| 3 | 3 | 1 | 1 | 2 | 0 (sn) | 0 | ° | ° | 1 | 0 | 16 | 70 | 50 | 10 | 0/1+ | ° | 1c | 0 | 1 | 0 | 0 | 1 | 0 | ° | 60,2 | ° | ° | ° |
| 4 | 12 | 1 | 1 | 3 | 0 (sn) | 0 | ° | ° | 1 | 0 | 0 | ° | ° | ° | ° | ° | is | 0 | 1 | 0 | 0 | 0 | 1 | 18/02/2019 | 9,7 | 1 | mammella + ascella | MAST + DA |

| | | | | | | | | | | | | | | | | | | | | | | | | | | | | |
|---|---|---|---|---|---|---|---|---|---|---|---|---|---|---|---|---|---|---|---|---|---|---|---|---|---|---|---|---|
| 4 | 4+4 | 1 | 1 | 1 | 1(sn) | 1 | 13 | 0 | 1 | 0 | 9 | 90 | 0 | 20 | 2+ | 1 | 1b | 1a | 1 | 0 | 1 | 1 | 0 | ° | 45,6 | ° | ° | ° |
| 4 | 12 | 1 | 1 | 2 | 1(sn) | 1 | 23 | 0 | 1 | 1 | 8 | 0 | 0 | 5 | 0/1+ | ° | 1b | 1a | 1 | 0 | 0 | 0 | 0 | ° | 34,6 | ° | ° | ° |
| 4 | 12+12 | 1 | 1 | 1 | 0 (sn) | 0 | ° | ° | 1 | 0 | 12 | 90 | 20 | 5 | 2+ | 1 | 1c | 0 | 1 | 0 | 1 | 1 | 0 | ° | 49,5 | ° | ° | ° |
| 4 | 12 | 1 | 1 | 3 | 1(sn) | 1 | 15 | 4 | 1 | 0 | 52 | 0 | 0 | 10 | 0/1+ | ° | 3 | 3a | 1 | 1 | 0 | 0 | 0 | ° | 4,8 | ° | ° | ° |
| 4 | 4 | 1 | 1 | 4 | 0 (sn) | 0 | ° | ° | 1 | 0 | 0 | ° | ° | ° | ° | ° | is | 0 | 1 | 1 | 0 | 1 | 0 | ° | 37,7 | ° | ° | ° |
| 4 | 12 | 1 | 1 | 1 | 0 (sn) | 0 | ° | ° | 1 | 1 | 16 | 95 | 20 | 5 | 0/1+ | ° | 1c | 0 | 1 | 0 | 0 | 1 | 0 | ° | 45,4 | ° | ° | ° |
| 4 | 12+12 | 1 | 1 | 1 | 0 (sn) | 0 | ° | ° | 1 | 1 | 3 | 90 | 0 | 50 | 3+ | ° | 1a | 0 | 1 | 0 | 1 | 1 | 0 | ° | 3,2 | ° | ° | ° |
| 4 | 12 | 1 | 1 | 2 | Mi(sn) | 0 | ° | ° | 1 | 0 | 6 | 0 | 0 | 70 | 0/1+ | ° | 1b | 1Mi(sn) | 1 | 0 | 0 | 0 | 0 | ° | 42,2 | ° | ° | ° |
| 4 | 12 | 2 | 1 | 1 | 0 (sn) | 0 | ° | ° | 1 | 0 | 6 | 90 | 0 | 2 | 0/1+ | ° | 1b | 0 | 0 | 0 | 0 | 1 | 0 | ° | 42,0 | ° | ° | ° |
| 4 | 12+12 | 2 | 1 | 2 | 1(sn) | 1 | 13 | 1 | 1 | 1 | 30 | 90 | 10 | 15 | 3+ | ° | 2 | 1a | 0 | 0 | 1 | 1 | 0 | ° | 2,0 | ° | ° | ° |
| 4 | 12 | 1 | 1 | 2 | 0 (sn) | 0 | ° | ° | 1 | 0 | 2 | 0 | 0 | 60 | 0/1+ | ° | 1a | 0 | 1 | 1 | 0 | 0 | 0 | ° | 0,9 | ° | ° | ° |
| 4 | 12 | 1 | 1 | 2 | 0 (sn) | 0 | ° | ° | 1 | 0 | 0 | ° | ° | ° | ° | ° | is | 0 | 1 | 0 | 0 | 1 | 0 | ° | 34,5 | ° | ° | ° |
| 4 | 4 | 1 | 1 | 2 | 0 (sn) | 0 | ° | ° | 1 | 0 | 13 | 0 | 0 | 80 | 0/1+ | ° | 1c | 0 | 1 | 1 | 0 | 0 | 0 | ° | 33,4 | ° | ° | ° |
| 4 | 12+12 | 1 | 1 | 4 | 0 (sn) | 0 | ° | ° | 1 | 0 | 0 | ° | ° | ° | ° | ° | is | 0 | 1 | 0 | 1 | 0 | 1 | 10/10/2022 | 31,5 | 2 | ° | ° |
| 4 | 6 | 1 | 1 | 1 | 0 (sn) | 0 | ° | ° | 1 | 0 | 6 | 0 | 0 | 40 | 0/1+ | ° | 1b | 0 | 1 | 0 | 0 | 0 | 1 | 06/06/2018 | 8,4 | 1 | mammella | MAST |
| 4 | 4+4 | 2 | 1 | 1 | 1(sn) | 1 | 16 | 0 | 1 | 1 | 13 | 90 | 5 | 5 | 0/1+ | ° | 1c | 1a | 1 | 0 | 1 | 1 | 0 | ° | 61,2 | ° | ° | ° |
| 4 | 4+4 | 1 | 1 | 2 | 0 (sn) | 0 | ° | ° | 1 | 0 | 0 | ° | ° | ° | ° | ° | 0 | 0 | 1 | 0 | 1 | 0 | 0 | ° | 59,5 | ° | ° | ° |
| 4 | 2 | 1 | 1 | 1 | 0 (sn) | 0 | ° | ° | 1 | 0 | 37 | 0 | 0 | 80 | 0/1+ | ° | 2 | 0 | 1 | 1 | 0 | 0 | 0 | ° | 66,3 | ° | ° | ° |
| 4 | 4+4 | 2 | 1 | 1 | 0 (sn) | 0 | ° | ° | 1 | 0 | 0 | ° | ° | ° | ° | ° | 0 | 0 | 1 | 0 | 1 | 0 | 0 | ° | 59,6 | ° | ° | ° |
| 4 | 12 | 1 | 1 | 1 | Mi(sn) | 1 | 10 | 0 | 1 | 0 | 14 | 80 | 20 | 13 | 0/1+ | ° | 1c | 1Mi(sn) | 1 | 0 | 0 | 1 | 0 | ° | 60,7 | ° | ° | ° |
| 4 | 12 | 2 | 1 | 1 | 0 (sn) | 0 | ° | ° | 1 | 0 | 0 | ° | ° | ° | ° | ° | is | 0 | 0 | 0 | 0 | 0 | 1 | 18/01/2019 | 10,6 | 1 | ascella | DA |
| 4 | 4+4 | 1 | 1 | 1 | 0 (sn) | 0 | ° | ° | 1 | 0 | 11 | 0 | 0 | 30 | 3+ | ° | 1c | 0 | 1 | 0 | 1 | 0 | 0 | ° | 56,7 | ° | ° | ° |
| 4 | 10+10 | 2 | 1 | 1 | Mi(sn) | 0 | ° | ° | 1 | 0 | 19 | 0 | 0 | 5 | 0/1+ | ° | 1c | 1Mi(sn) | 1 | 0 | 0 | 0 | 0 | ° | 54,4 | ° | ° | ° |
| 4 | 12+12 | 1 | 1 | 2 | 1(sn) | 1 | 20 | 1 | 1 | 1 | 27 | 90 | 16 | 5 | 2+ | 1 | 2 | 1a | 1 | 0 | 1 | 1 | 0 | ° | 55,0 | ° | ° | ° |
| 4 | 4+4 | 1 | 1 | 2 | 0 (sn) | 0 | ° | ° | 1 | 0 | 0 | ° | ° | ° | ° | ° | 0 | 0 | 1 | 0 | 1 | 1 | 0 | ° | 55,3 | ° | ° | ° |
| 4 | 4 | 1 | 1 | 3 | 0 (sn) | 0 | ° | ° | 1 | 0 | 0 | ° | ° | ° | ° | ° | 0 | 0 | 1 | 0 | 0 | 0 | 0 | ° | 53,2 | ° | ° | ° |
| 4 | 4+4 | 1 | 1 | 1 | 0 (sn) | 0 | ° | ° | 1 | 0 | 9 | 90 | 70 | 5 | 3+ | ° | 1b | 0 | 1 | 0 | 1 | 1 | 0 | ° | 55,3 | ° | ° | ° |
| 4 | 12 | 2 | 1 | 1 | 0 (sn) | 0 | ° | ° | 1 | 0 | 0 | ° | ° | ° | ° | ° | 0 | 0 | 0 | 0 | 0 | 0 | 1 | 15/09/2019 | 14,9 | 2 | ° | ° |
| 4 | 4 | 1 | 1 | 2 | 1(sn) | 1 | 10 | 3 | 1 | 1 | 12 | 90 | 70 | 10 | 0/1+ | ° | 1c | 2a | 1 | 0 | 0 | 1 | 0 | ° | 53,8 | ° | ° | ° |
| 4 | 4+4 | 2 | 1 | 1 | 0 (sn) | 0 | ° | ° | 1 | 0 | 9 | 50 | 20 | 10 | 3+ | ° | 1b | 0 | 1 | 0 | 1 | 1 | 0 | ° | 53,5 | ° | ° | ° |
| 4 | 4+4 | 1 | 1 | 1 | 0 (sn) | 0 | ° | ° | 1 | 0 | 0 | ° | ° | ° | ° | ° | 0 | 0 | 1 | 0 | 1 | 0 | 0 | ° | 36,3 | ° | ° | ° |
| 4 | 4+4 | 1 | 1 | 3 | 0 (sn) | 0 | ° | ° | 1 | 0 | 0 | ° | ° | ° | ° | ° | is | 0 | 1 | 0 | 1 | 0 | 0 | ° | 55,4 | ° | ° | ° |
| 4 | 3 | 2 | 1 | 1 | 0 (sn) | 0 | ° | ° | 1 | 0 | 12 | 0 | 0 | 35 | 2+ | 1 | 1c | 0 | 0 | 1 | 1 | 0 | 0 | ° | 12,4 | ° | ° | ° |
| 4 | 4 | 1 | 1 | 1 | 1(sn) | 1 | 12 | 0 | 0 | 0 | 12 | 90 | 0 | 6 | 0/1+ | ° | 1c | 1a | 1 | 0 | 0 | 1 | 0 | ° | 53,0 | ° | ° | ° |
| 4 | 12 | 2 | 1 | 1 | 0 (sn) | 0 | ° | ° | 1 | 0 | 13 | 95 | 0 | 10 | 0/1+ | ° | 1c | 0 | 1 | 0 | 0 | 1 | 0 | ° | 53,3 | ° | ° | ° |
| 4 | 12 | 2 | 1 | 1 | 0 (sn) | 0 | ° | ° | 1 | 0 | 30 | 0 | 0 | 90 | 2+ | 0 | 2 | 0 | 0 | 1 | 0 | 0 | 0 | ° | 59,9 | ° | ° | ° |
| 4 | 11 | 1 | 1 | 1 | 0 (sn) | 0 | ° | ° | 1 | 0 | 7 | 0 | 0 | 25 | 0/1+ | ° | 1b | 0 | 1 | 0 | 0 | 0 | 0 | ° | 51,7 | ° | ° | ° |
| 4 | ° | 1 | 1 | 1 | 1(sn) | 1 | 7 | 4 | 1 | 0 | 8 | 80 | 70 | 5 | 0/1+ | ° | 1b | 2a | 1 | 1 | 0 | 1 | 0 | ° | 55,1 | ° | ° | ° |
| 4 | 4+4 | 1 | 1 | 1 | 1(sn) | 1 | 13 | 1 | 1 | 1 | 22 | 90 | 90 | 18 | 2+ | 0 | 2 | 1a | 1 | 0 | 0 | 1 | 0 | ° | 51,9 | ° | ° | ° |
| 4 | 12 | 2 | 1 | 1 | 0 (sn) | 0 | ° | ° | 1 | 0 | 2 | 0 | 0 | 80 | 0/1+ | ° | 1a | 0 | 0 | 1 | 0 | 0 | 0 | ° | 51,6 | ° | ° | ° |
| 4 | 12 | 2 | 1 | 1 | 1(sn) | 1 | 15 | 13 | 1 | 1 | 70 | 95 | 30 | 15 | 0/1+ | ° | 4d | 3a | 1 | 0 | 0 | 1 | 0 | ° | 53,3 | ° | ° | ° |
| 4 | 4+4 | 2 | 1 | 2 | 0 (sn) | 0 | ° | ° | 1 | 1 | 7 | 90 | 60 | 5 | 3+ | ° | 1b | 0 | 1 | 0 | 1 | 1 | 0 | ° | 51,2 | ° | ° | ° |

| | | | | | | | | | | | | | | | | | | | | | | | | | | | | |
|---|---|---|---|---|---|---|---|---|---|---|---|---|---|---|---|---|---|---|---|---|---|---|---|---|---|---|---|---|
| 4 | 12+12 | 1 | 1 | 1 | 0 (sn) | 0 | ° | ° | 1 | 0 | 0 | ° | ° | ° | ° | ° | 0 | 0 | 1 | 0 | 1 | 0 | 0 | ° | 35,8 | ° | ° | ° |
| 4 | 4+4 | 2 | 1 | 1 | 0 (sn) | 0 | ° | ° | 1 | 1 | 6 | 0 | 0 | 10 | 3+ | ° | 1b | 0 | 1 | 0 | 1 | 1 | 0 | ° | 50,9 | ° | ° | ° |
| 4 | 12+12 | 1 | 1 | 1 | 0 (sn) | 0 | ° | ° | 1 | 1 | 4 | 30 | 0 | 15 | 3+ | ° | 1a | 0 | 1 | 1 | 1 | 1 | 0 | ° | 53,8 | ° | ° | ° |
| 4 | 4+4 | 1 | 1 | 1 | 0 (sn) | 0 | ° | ° | 1 | 0 | 0 | ° | ° | ° | ° | ° | 1mic | 0 | 1 | 0 | 1 | 0 | 0 | ° | 51,4 | ° | ° | ° |
| 4 | 12 | 1 | 1 | 1 | 0 (sn) | 0 | ° | ° | 1 | 0 | 31 | 0 | 0 | 80 | 0/1+ | ° | 2 | 0 | 1 | 1 | 0 | 0 | 0 | ° | 49,8 | ° | ° | ° |
| 4 | 12 | 1 | 1 | 2 | 1(sn) | 1 | 8 | 1 | 1 | 0 | 21 | 90 | 70 | 15 | 0/1+ | ° | 2 | 1a | 1 | 0 | 0 | 1 | 0 | ° | 32,5 | ° | ° | ° |
| 4 | 12 | 1 | 1 | 3 | 0 (sn) | 0 | ° | ° | 1 | 0 | 0 | ° | ° | ° | ° | ° | 0 | 0 | 1 | 0 | 0 | 0 | 0 | ° | 55,8 | ° | ° | ° |
| 4 | 12+12 | 1 | 1 | 3 | 0 (sn) | 0 | ° | ° | 1 | 0 | 11 | 70 | 40 | 10 | 0/1+ | ° | 1c | 0 | 1 | 0 | 0 | 1 | 0 | ° | 49,0 | ° | ° | ° |
| 4 | 12 | 1 | 1 | 1 | 0 (sn) | 0 | ° | ° | 1 | 0 | 18 | 0 | 0 | 70 | 0/1+ | ° | 1c | 0 | 1 | 1 | 0 | 0 | 0 | ° | 48,6 | ° | ° | ° |
| 4 | 12 | 2 | 1 | 1 | 0 (sn) | 0 | ° | ° | 1 | 0 | 0 | ° | ° | ° | ° | ° | is | 0 | 0 | 0 | 0 | 0 | 0 | ° | 45,1 | ° | ° | ° |
| 4 | 12+12 | 2 | 1 | 3 | 0 (sn) | 0 | ° | ° | 1 | 0 | 0 | ° | ° | ° | ° | ° | is | 0 | 0 | 0 | 1 | 0 | 0 | ° | 46,5 | ° | ° | ° |
| 4 | 3+3 | 1 | 1 | 1 | 0 (sn) | 0 | ° | ° | 1 | 0 | 9 | 90 | 0 | 15 | 3+ | ° | 1b | 0 | 1 | 0 | 1 | 1 | 0 | ° | 46,5 | ° | ° | ° |
| 4 | 4+4 | 2 | 1 | 1 | 0 (sn) | 0 | ° | ° | 1 | 0 | 12 | 90 | 35 | 5 | 2+ | 1 | 1c | 0 | 0 | 0 | 1 | 1 | 0 | ° | 44,3 | ° | ° | ° |
| 4 | 12 | 1 | 1 | 1 | 0 (sn) | 0 | ° | ° | 1 | 0 | 3 | 0 | 0 | 25 | 0/1+ | ° | 1a | 0 | 1 | 0 | 0 | 0 | 0 | ° | 0,5 | ° | ° | ° |
| 4 | 4+4 | 1 | 1 | 1 | 0 (sn) | 0 | ° | ° | 1 | 0 | 8 | 90 | 85 | 12 | 2+ | 1 | 1b | 0 | 1 | 0 | 1 | 1 | 0 | ° | 43,7 | ° | ° | ° |
| 4 | 12 | 1 | 1 | 4 | 0 (sn) | 0 | ° | ° | 1 | 0 | 13 | 0 | 0 | 15 | 0/1+ | ° | 1c | 0 | 1 | 1 | 0 | 0 | 0 | ° | 44,4 | ° | ° | ° |
| 4 | 12+12 | 1 | 1 | 1 | 0 (sn) | 0 | ° | ° | 1 | 0 | 0 | ° | ° | ° | ° | ° | 0 | 0 | 1 | 0 | 1 | 1 | 0 | ° | 45,8 | ° | ° | ° |
| 4 | 4 | 1 | 1 | 2 | 1(sn) | 1 | 27 | 2 | 1 | 0 | 6 | 80 | 0 | 5 | 0/1+ | ° | 1b | 1a | 1 | 0 | 0 | 1 | 0 | ° | 44,6 | ° | ° | ° |
| 4 | 12 | 2 | 1 | 1 | 0 (sn) | 0 | ° | ° | 1 | 0 | 0 | ° | ° | ° | ° | ° | is | 0 | 1 | 0 | 0 | 1 | 0 | ° | 48,5 | ° | ° | ° |
| 4 | 4 | 1 | 1 | 2 | 1(sn) | 1 | 9 | 5 | 1 | 1 | 32 | 90 | 55 | 20 | 2+ | 0 | 2 | 2a | 1 | 0 | 0 | 1 | 1 | 15/01/2021 | 22,7 | 2 | ° | ° |
| 4 | 2 | 1 | 1 | 1 | 0 (sn) | 0 | ° | ° | 1 | 0 | 0 | ° | ° | ° | ° | ° | 0 | 0 | 1 | 0 | 0 | 0 | 0 | ° | 49,6 | ° | ° | ° |
| 3 | 11 | 2 | 1 | 3 | 1(sn) | 1 | 13 | 0 | 1 | 0 | 0 | ° | ° | ° | ° | ° | 0 | 1a | 1 | 1 | 0 | 0 | 0 | ° | 40,0 | ° | ° | ° |
| 3 | ° | 2 | 1 | 1 | 0 (sn) | 0 | ° | ° | 1 | 0 | 17 | 0 | 0 | 75 | 2+ | 0 | 1c | 0 | 1 | 1 | 0 | 0 | 0 | ° | 29,4 | ° | ° | ° |
| 4 | 4 | 2 | 1 | 1 | 0 (sn) | 0 | ° | ° | 1 | 1 | 35 | 80 | 5 | 60 | 0/1+ | ° | 2 | 0 | 1 | 0 | 0 | 1 | 1 | 15/07/2022 | 34,4 | 2 | ° | ° |
| 4 | 12 | 1 | 1 | 3 | 1(sn) | 1 | 25 | 6 | 1 | 0 | 40 | 60 | 0 | 3 | 0/1+ | ° | 2 | 2a | 1 | 0 | 0 | 1 | 0 | ° | 46,9 | ° | ° | ° |
| 4 | 12 | 1 | 1 | 2 | 0 (sn) | 0 | ° | ° | 1 | 0 | 22 | 0 | 0 | 85 | 0/1+ | ° | 2 | 0 | 1 | 0 | 0 | 0 | 0 | ° | 46,2 | ° | ° | ° |
| 4 | 4+4 | 2 | 1 | 2 | 0 (sn) | 0 | ° | ° | 1 | 0 | 12 | 70 | 0 | 5 | 0/1+ | ° | 1c | 0 | 0 | 0 | 1 | 1 | 1 | 01/06/2021 | 21,4 | 2 | ° | ° |
| 4 | 4+4 | 2 | 1 | 1 | 0 (sn) | 0 | ° | ° | 1 | 0 | 0 | ° | ° | ° | ° | ° | 0 | 0 | 0 | 0 | 1 | 1 | 0 | ° | 40,8 | ° | ° | ° |
| 4 | 12 | 1 | 1 | 1 | 0 (sn) | 0 | ° | ° | 1 | 0 | 1 | 90 | 90 | 10 | 0/1+ | ° | 1a | 0 | 1 | 0 | 0 | 1 | 0 | ° | 39,5 | ° | ° | ° |
| 4 | 4+4 | 2 | 1 | 2 | 1(sn) | 1 | 11 | 0 | 1 | 0 | 0 | ° | ° | ° | ° | ° | 0 | 1a | 1 | 0 | 1 | 0 | 1 | 09/12/2021 | 24,6 | 2 | ° | ° |
| 4 | 4+4 | 1 | 1 | 1 | 0 (sn) | 0 | ° | ° | 1 | 0 | 0 | ° | ° | ° | ° | ° | 0 | 0 | 1 | 0 | 1 | 1 | 0 | ° | 37,9 | ° | ° | ° |
| 4 | ° | 1 | 1 | 1 | 0 (sn) | 0 | ° | ° | 1 | 0 | 27 | 90 | 5 | 30 | 0/1+ | ° | 2 | 0 | 1 | 0 | 0 | 1 | 0 | ° | 44,8 | ° | ° | ° |
| 4 | 4 | 1 | 1 | 1 | 1(sn) | 1 | 12 | 7 | 1 | 0 | 14 | 80 | 50 | 80 | 0/1+ | ° | 1c | 2a | 1 | 0 | 0 | 1 | 0 | ° | 37,3 | ° | ° | ° |
| 4 | 12 | 2 | 1 | 1 | 0 (sn) | 0 | ° | ° | 1 | 0 | 0 | ° | ° | ° | ° | ° | is | 0 | 0 | 0 | 0 | 0 | 0 | ° | 35,1 | ° | ° | ° |
| 4 | ° | 2 | 1 | 1 | 1(sn) | 1 | 6 | 0 | 1 | 1 | 15 | 95 | 0 | 12 | 2+ | 1 | 1c | 1a | 1 | 1 | 1 | 1 | 0 | ° | 38,8 | ° | ° | ° |
| 4 | 4+4 | 1 | 1 | 3 | 0 (sn) | 0 | ° | ° | 1 | 0 | 0 | ° | ° | ° | ° | ° | is | 0 | 1 | 0 | 1 | 0 | 0 | ° | 35,3 | ° | ° | ° |
| 4 | 4+4 | 1 | 1 | 1 | 0 (sn) | 0 | ° | ° | 1 | 0 | 6 | 85 | 0 | 20 | 3+ | ° | 1b | 0 | 1 | 0 | 1 | 1 | 0 | ° | 35,5 | ° | ° | ° |
| 4 | 12+12 | 1 | 1 | 1 | 0 (sn) | 0 | ° | ° | 1 | 0 | 0 | ° | ° | ° | ° | ° | is | 0 | 1 | 0 | 1 | 1 | 0 | ° | 33,6 | ° | ° | ° |
| 4 | ° | 2 | 1 | 1 | 1(sn) | 1 | 5 | 0 | 1 | 0 | 25 | 30 | 15 | 8 | 0/1+ | ° | 2 | 1a | 1 | 1 | 0 | 1 | 1 | 02/10/2020 | 10,3 | 2 | ° | ° |
| 4 | 12+12 | 1 | 1 | 1 | Mi(sn) | 0 | ° | ° | 1 | 0 | 0 | ° | ° | ° | ° | ° | 0 | 1Mi(sn) | 1 | 0 | 1 | 0 | 0 | ° | 32,1 | ° | ° | ° |
| 4 | 12+12 | 1 | 1 | 4 | 0 (sn) | 0 | ° | ° | 1 | 0 | 28 | 95 | 75 | 30 | 3+ | ° | 2 | 0 | 1 | 0 | 1 | 1 | 0 | ° | 35,3 | ° | ° | ° |
| 4 | 12 | 2 | 1 | 2 | 1(sn) | 1 | 7 | 2 | 1 | 1 | 18 | 0 | 0 | 12 | 0/1+ | ° | 1c | 1a | 0 | 1 | 0 | 0 | 1 | 15/04/2021 | 14,2 | 1 | ascella + cute | DA + exeresi cutanea |

| | | | | | | | | | | | | | | | | | | | | | | | | | | | | |
|---|---|---|---|---|---|---|---|---|---|---|---|---|---|---|---|---|---|---|---|---|---|---|---|---|---|---|---|---|
| 4 | 4+4 | 1 | 1 | 1 | 0 (sn) | 0 | ° | ° | 1 | 0 | 9 | 2 | 0 | 65 | 3+ | ° | 1b | 0 | 1 | 0 | 1 | 0 | 0 | ° | 31,5 | ° | ° | ° |
| 4 | 11 | 1 | 1 | 1 | 1(sn) | 1 | 8 | 0 | 1 | 0 | 0 | ° | ° | ° | ° | ° | is | 1a | 1 | 1 | 0 | 0 | 0 | ° | 31,5 | ° | ° | ° |
| 4 | 12+12 | 1 | 1 | 1 | 0 (sn) | 0 | ° | ° | 1 | 0 | 6 | 90 | 60 | 5 | 3+ | ° | 1b | 0 | 1 | 0 | 1 | 1 | 0 | ° | 31,4 | ° | ° | ° |
| 4 | 4+4 | 1 | 1 | 1 | 0 (sn) | 0 | ° | ° | 1 | 0 | 9 | 95 | 2 | 2 | 3+ | ° | 1b | 0 | 1 | 0 | 1 | 1 | 0 | ° | 31,3 | ° | ° | ° |
| 4 | 4 | 1 | 1 | 1 | 0 (sn) | 0 | ° | ° | 1 | 0 | 4 | 0 | 2 | 80 | 0/1+ | ° | 1a | 0 | 1 | 1 | 0 | 0 | 0 | ° | 31,1 | ° | ° | ° |
| 4 | 4+4 | 1 | 1 | 3 | 0 (sn) | 0 | ° | ° | 1 | 0 | 15 | 0 | 0 | 6 | 2+ | 1 | 1c | 0 | 1 | 0 | 1 | 0 | 0 | ° | 31,1 | ° | ° | ° |
| 4 | 12+12 | 1 | 1 | 1 | 0 (sn) | 0 | ° | ° | 1 | 0 | 0 | ° | ° | ° | ° | ° | 0 | 0 | 1 | 0 | 1 | 0 | 1 | 17/06/2022 | 25,2 | 1 | mammella | QUAD |
| 12 | 12 | 1 | 1 | 1 | 0 (sn) | 0 | ° | ° | 1 | 0 | 0 | ° | ° | ° | ° | ° | 0 | 0 | 1 | 0 | 1 | 0 | 0 | ° | 30,8 | ° | ° | ° |
| 4 | 12+12 | 1 | 1 | 2 | 0 (sn) | 0 | ° | ° | 1 | 0 | 0 | ° | ° | ° | ° | ° | 0 | 0 | 1 | 0 | 1 | 0 | 0 | ° | 30,8 | ° | ° | ° |
| 4 | 4 | 1 | 1 | 1 | Mi(sn) | 1 | 7 | 0 | 1 | 0 | 0 | ° | ° | ° | ° | ° | is | 1Mi(sn) | 1 | 0 | 1 | 0 | 0 | ° | 30,6 | ° | ° | ° |
| 4 | 10 | 1 | 1 | 2 | 0 (sn) | 0 | ° | ° | 1 | 0 | 0 | ° | ° | ° | ° | ° | 0 | 0 | 1 | 0 | 0 | 0 | 0 | ° | 30,6 | ° | ° | ° |
| 4 | 3 | 1 | 1 | 1 | 0 (sn) | 0 | ° | ° | 1 | 0 | 0 | ° | ° | ° | ° | ° | 0 | 0 | 1 | 0 | 0 | 0 | 0 | ° | 30,4 | ° | ° | ° |
| 3 | 4 | 1 | 1 | 2 | 1(sn) | 1 | 5 | 0 | 1 | 0 | 16 | 50 | 0 | 8 | 2+ | 1 | 1c | 1a | 1 | 0 | 1 | 1 | 0 | ° | 30,4 | ° | ° | ° |
| 4 | 4+4 | 1 | 1 | 1 | 0 (sn) | 0 | ° | ° | 1 | 0 | 12 | 0 | 0 | 40 | 0/1+ | ° | 1c | 0 | 1 | 0 | 1 | 0 | 0 | ° | 30,3 | ° | ° | ° |
| 4 | 4+4 | 2 | 1 | 1 | 0 (sn) | 0 | ° | ° | 1 | 0 | 0 | ° | ° | ° | ° | ° | is | 0 | 0 | 0 | 1 | 1 | 0 | ° | 30,2 | ° | ° | ° |
| 4 | 12 | 1 | 1 | 1 | 0 (sn) | 0 | ° | ° | 1 | 0 | 0 | ° | ° | ° | ° | ° | 0 | 0 | 1 | 0 | 0 | 0 | 0 | ° | 30,2 | ° | ° | ° |
| 4 | 12 | 2 | 1 | 2 | 0 (sn) | 0 | ° | ° | 1 | 0 | 0 | ° | ° | ° | ° | ° | 0 | 0 | 0 | 0 | 0 | 0 | 0 | ° | 30,1 | ° | ° | ° |
| 4 | 12+12 | 2 | 1 | 3 | 0 (sn) | 0 | ° | ° | 0 | 0 | 12 | 90 | 0 | 12 | 2+ | 1 | 1c | 0 | 0 | 0 | 1 | 1 | 0 | ° | 29,9 | ° | ° | ° |
| 4 | 12 | 1 | 1 | 1 | Mi(sn) | 1 | 20 | 0 | 1 | 0 | 12 | 0 | 0 | 70 | 0/1+ | ° | 1c | 1Mi(sn) | 1 | 1 | 0 | 0 | 0 | ° | 29,7 | ° | ° | ° |
| 4 | 12+12 | 1 | 1 | 1 | 0 (sn) | 0 | ° | ° | 1 | 1 | 8 | 80 | 5 | 5 | 2+ | 1 | 1b | 0 | 1 | 0 | 1 | 1 | 0 | ° | 29,7 | ° | ° | ° |
| 4 | 12+12 | 1 | 1 | 1 | 0 (sn) | 0 | ° | ° | 1 | 0 | 8 | 80 | 0 | 3 | 3+ | ° | 1 | 0 | 1 | 0 | 1 | 0 | 0 | ° | 29,6 | ° | ° | ° |
| 4 | 2+4 | 1 | 1 | 3 | Mi(sn) | 0 | ° | ° | 1 | 0 | 0 | ° | ° | ° | ° | ° | 1mic | 1Mi(sn) | 1 | 0 | 1 | 1 | 0 | ° | 29,3 | ° | ° | ° |
| 4 | 12 | 1 | 1 | 3 | 0 (sn) | 0 | ° | ° | 1 | 0 | 8 | 0 | 0 | 80 | 0/1+ | ° | 1b | 0 | 1 | 1 | 0 | 0 | 0 | ° | 28,9 | ° | ° | ° |
| 4 | 12 | 2 | 1 | 1 | 0 (sn) | 0 | ° | ° | 1 | 0 | 0 | ° | ° | ° | ° | ° | 0 | 0 | 0 | 0 | 0 | 0 | 0 | ° | 28,8 | ° | ° | ° |
| 4 | 12 | 1 | 1 | 1 | 0 (sn) | 0 | ° | ° | 1 | 0 | 0 | ° | ° | ° | ° | ° | 0 | 0 | 1 | 0 | 0 | 1 | 0 | ° | 28,6 | ° | ° | ° |
| 4 | 10 | 2 | 1 | 2 | 0 (sn) | 0 | ° | ° | 1 | 0 | 0 | ° | ° | ° | ° | ° | 0 | 0 | 0 | 0 | 0 | 0 | 0 | ° | 28,6 | ° | ° | ° |
| 4 | 4+4 | 2 | 1 | 2 | 0 (sn) | 0 | ° | ° | 1 | 0 | 0 | ° | ° | ° | ° | ° | is | 0 | 0 | 0 | 1 | 0 | 1 | 02/10/2020 | 1,9 | 2 | ° | ° |
| 4 | 12 | 1 | 1 | 1 | 1(sn) | 1 | 10 | 0 | 1 | 0 | 21 | 0 | 0 | 90 | 0/1+ | ° | 2 | 1a | 1 | 1 | 1 | 0 | 1 | 16/05/2022 | 21,4 | 2 | ° | ° |
| 4 | 12+12 | 2 | 1 | 2 | 0 (sn) | 0 | ° | ° | 1 | 0 | 0 | ° | ° | ° | ° | ° | is | 0 | 0 | 0 | 1 | 1 | 0 | ° | 28,3 | ° | ° | ° |
| 4 | 12 | 1 | 1 | 2 | 0 (sn) | 0 | ° | ° | 1 | 0 | 0 | ° | ° | ° | ° | ° | 0 | 0 | 1 | 0 | 0 | 1 | 0 | ° | 28,1 | ° | ° | ° |
| 4 | 12+12 | 1 | 1 | 2 | 0 (sn) | 0 | ° | ° | 1 | 0 | 9 | 0 | 0 | 10 | 3+ | ° | 1b | 0 | 1 | 0 | 1 | 0 | 0 | ° | 28,0 | ° | ° | ° |
| 4 | 12+12 | 1 | 1 | 3 | 0 (sn) | 0 | ° | ° | 1 | 0 | 0 | ° | ° | ° | ° | ° | is | 0 | 1 | 0 | 1 | 0 | 0 | ° | 27,6 | ° | ° | ° |
| 4 | 12+12 | 2 | 1 | 1 | 1(sn) | 1 | 11 | 0 | 1 | 0 | 15 | 90 | 70 | 6 | 2+ | 1 | 1c | 1a | 0 | 0 | 1 | 1 | 0 | ° | 27,5 | ° | ° | ° |
| 4 | 12+12 | 1 | 1 | 2 | 0 (sn) | 0 | ° | ° | 1 | 0 | 0 | ° | ° | ° | ° | ° | is | 0 | 1 | 0 | 1 | 1 | 0 | ° | 27,3 | ° | ° | ° |
| 4 | 12 | 2 | 1 | 1 | 1(sn) | 1 | 11 | 0 | 1 | 0 | 4 | 0 | 0 | 5 | 0/1+ | ° | 1a | 1a | 1 | 1 | 0 | 0 | 0 | ° | 27,1 | ° | ° | ° |
| 4 | 12 | 2 | 1 | 1 | 0 (sn) | 0 | ° | ° | 2 | 0 | 10 | 75 | 70 | 5 | 0/1+ | ° | 1b | 0 | 0 | 0 | 0 | 1 | 0 | ° | 26,9 | ° | ° | ° |
| 4 | 12+12 | 1 | 1 | 2 | 0 (sn) | 0 | ° | ° | 1 | 0 | 0 | ° | ° | ° | ° | ° | 0 | 0 | 1 | 0 | 1 | 0 | 0 | ° | 26,2 | ° | ° | ° |
| 4 | 4+4 | 1 | 1 | 1 | 1(sn) | 1 | 11 | 1 | 1 | 1 | 25 | 90 | 10 | 5 | 3+ | ° | 2 | 1a | 1 | 0 | 1 | 1 | 0 | ° | 26,2 | ° | ° | ° |
| 4 | 12 | 2 | 1 | 3 | 1(sn) | 1 | 12 | 0 | 1 | 0 | 25 | 90 | 30 | 10 | 0/1+ | ° | 2 | 1a | 1 | 0 | 0 | 1 | 0 | ° | 25,9 | ° | ° | ° |
| 4 | 12 | 1 | 1 | 3 | 0 (sn) | 0 | ° | ° | 1 | 0 | 0 | ° | ° | ° | ° | ° | 0 | 0 | 1 | 0 | 0 | 0 | 0 | ° | 25,8 | ° | ° | ° |
| 4 | 12 | 1 | 1 | 2 | 0 (sn) | 0 | ° | ° | 1 | 0 | 2 | 0 | 0 | 2 | 0/1+ | ° | 1a | 0 | 1 | 0 | 0 | 0 | 0 | ° | 25,3 | ° | ° | ° |
| 4 | 12 | 2 | 1 | 4 | 0 (sn) | 0 | ° | ° | 1 | 0 | 0 | ° | ° | ° | ° | ° | 0 | 0 | 0 | 0 | 0 | 0 | 0 | ° | 24,5 | ° | ° | ° |

| | | | | | | | | | | | | | | | | | | | | | | | | | | | | |
|---|---|---|---|---|---|---|---|---|---|---|---|---|---|---|---|---|---|---|---|---|---|---|---|---|---|---|---|---|
| 4 | 12+12 | 2 | 1 | 2 | 0 (sn) | 0 | ° | ° | 1 | 0 | 0 | ° | ° | ° | ° | ° | is | 0 | 0 | 0 | 1 | 1 | 0 | ° | 24,3 | ° | ° | ° |
| 4 | 12+12 | 2 | 1 | 2 | 0 (sn) | 0 | ° | ° | 1 | 0 | 0 | ° | ° | ° | ° | ° | 0 | 0 | 0 | 0 | 1 | 0 | 0 | ° | 24,3 | ° | ° | ° |
| 4 | 4+4 | 2 | 1 | 1 | 0 (sn) | 0 | ° | ° | 1 | 0 | 0 | ° | ° | ° | ° | ° | 0 | 0 | 0 | 0 | 1 | 0 | 0 | ° | 24,1 | ° | ° | ° |
| 4 | 4 | 2 | 1 | 1 | 1(sn) | 1 | 11 | 2 | 1 | 0 | 15 | 95 | 80 | 5 | 0/1+ | ° | 1c | 1a | 1 | 0 | 0 | 1 | 0 | ° | 23,9 | ° | ° | ° |
| 4 | 12 | 2 | 1 | 1 | 1(sn) | 1 | 11 | 2 | 1 | 1 | 26 | 90 | 25 | 10 | 0/1+ | ° | 2 | 2a | 1 | 0 | 0 | 1 | 0 | ° | 23,7 | ° | ° | ° |
| 4 | 4 | 2 | 1 | 1 | 1(sn) | 1 | 18 | 6 | 1 | 1 | 45 | 90 | 90 | 15 | 2+ | 1 | 2 | 2a | 1 | 1 | 1 | 1 | 0 | ° | 23,4 | ° | ° | ° |
| 4 | 12 | 1 | 1 | 3 | 1(sn) | 0 | ° | ° | 1 | 0 | 14 | 0 | 0 | 75 | 0/1+ | ° | 1c | 1a | 1 | 1 | 0 | 0 | 0 | ° | 22,5 | ° | ° | ° |
| 4 | 12 | 1 | 1 | 1 | 0 (sn) | 0 | ° | ° | 1 | 0 | 11 | 0 | 0 | 35 | 0/1+ | ° | 1c | 0 | 1 | 1 | 0 | 0 | 0 | ° | 22,4 | ° | ° | ° |
| 4 | 4+4 | 1 | 1 | 2 | 0 (sn) | 0 | ° | ° | 1 | 0 | 11 | 60 | 20 | 10 | 3+ | ° | 1c | 0 | 1 | 0 | 1 | 1 | 0 | ° | 22,3 | ° | ° | ° |
| 4 | 4+4 | 1 | 1 | 3 | 0 (sn) | 0 | ° | ° | 1 | 0 | 0 | ° | ° | ° | ° | ° | 0 | 0 | 1 | 0 | 1 | 0 | 0 | ° | 22,0 | ° | ° | ° |
| 4 | 12 | 2 | 1 | 3 | 0 (sn) | 0 | ° | ° | 1 | 0 | 40 | 0 | 0 | 70 | 0/1+ | ° | 2 | 0 | 0 | 1 | 0 | 0 | 1 | 15/11/2021 | 9,0 | 2 | ° | ° |
| 4 | 12 | 2 | 1 | 1 | 1(sn) | 1 | 10 | 0 | 1 | 1 | 24 | 90 | 80 | 5 | 0/1+ | ° | 2 | 1a | 1 | 0 | 0 | 1 | 0 | ° | 22,0 | ° | ° | ° |
| 4 | 12 | 1 | 1 | 1 | 1(sn) | 1 | 16 | 0 | 1 | 0 | 14 | 0 | 0 | 80 | 0/1+ | ° | 1c | 1a | 1 | 1 | 0 | 0 | 0 | ° | 21,6 | ° | ° | ° |
| 4 | 4+4 | 1 | 1 | 3 | 0 (sn) | 0 | ° | ° | 1 | 0 | 0 | ° | ° | ° | ° | ° | 0 | 0 | 1 | 0 | 1 | 0 | 0 | ° | 21,6 | ° | ° | ° |
| 4 | 12+12 | 1 | 1 | 1 | 0 (sn) | 0 | ° | ° | 1 | 0 | 4 | 0 | 0 | 25 | 3+ | ° | 1a | 0 | 1 | 0 | 1 | 0 | 0 | ° | 21,5 | ° | ° | ° |
| 4 | 12+12 | 1 | 1 | 2 | 0 (sn) | 0 | ° | ° | 1 | 0 | 0 | ° | ° | ° | ° | ° | 1mic | 0 | 1 | 0 | 1 | 1 | 0 | ° | 21,3 | ° | ° | ° |
| 4 | 4+4 | 2 | 1 | 1 | 0 (sn) | 0 | ° | ° | 1 | 0 | 36 | 0 | 0 | 65 | 3+ | ° | 2 | 0 | 0 | 0 | 1 | 0 | 1 | 09/11/2021 | 7,9 | 2 | ° | ° |
| 4 | 12+12 | 1 | 1 | 3 | 0 (sn) | 0 | ° | ° | 1 | 1 | 12 | 0 | 0 | 10 | 3+ | ° | 1c | 0 | 1 | 0 | 1 | 0 | 0 | ° | 20,9 | ° | ° | ° |
| ° | ° | 1 | 1 | 2 | Mi(sn) | 0 | ° | ° | 1 | 0 | 0 | ° | ° | ° | ° | ° | 1mic | 1Mi(sn) | 0 | 1 | 0 | 1 | 0 | ° | 20,3 | ° | ° | ° |
| 4 | 12+12 | 2 | 1 | 1 | 0 (sn) | 0 | ° | ° | 1 | 0 | 8 | 90 | 0 | 5 | 3+ | ° | 1b | 0 | 0 | 0 | 1 | 1 | 0 | ° | 20,1 | ° | ° | ° |
| 4 | 4 | 1 | 1 | 3 | 0 (sn) | 0 | ° | ° | 1 | 0 | 0 | ° | ° | ° | ° | ° | 0 | 0 | 1 | 0 | 0 | 1 | 0 | ° | 19,9 | ° | ° | ° |
| 4 | 12+12 | 1 | 1 | 1 | 0 (sn) | 0 | ° | ° | 1 | 0 | 7 | 90 | 1 | 5 | 2+ | 1 | 1b | 0 | 1 | 0 | 1 | 1 | 0 | ° | 19,6 | ° | ° | ° |
| 4 | 4+4 | 2 | 1 | 4 | 0 (sn) | 0 | ° | ° | 1 | 0 | 13 | 90 | 25 | 5 | 3+ | ° | 1c | 0 | 0 | 0 | 1 | 1 | 0 | ° | 19,2 | ° | ° | ° |
| 4 | 12+12 | 1 | 1 | 1 | Mi(sn) | 1 | 20 | 0 | 1 | 1 | 12 | 90 | 10 | 15 | 3+ | ° | 1c | 1Mi(sn) | 1 | 0 | 1 | 1 | 0 | ° | 19,1 | ° | ° | ° |
| 4 | 12+12 | 1 | 1 | 1 | 1(sn) | 1 | 11 | 0 | 1 | 0 | 23 | 90 | 90 | 10 | 0/1+ | ° | 2 | 1a | 1 | 0 | 1 | 1 | 0 | ° | 18,7 | ° | ° | ° |
| 4 | 4+4 | 2 | 1 | 4 | 0 (sn) | 0 | ° | ° | 1 | 0 | 0 | ° | ° | ° | ° | ° | is | 0 | 0 | 0 | 1 | 1 | 0 | ° | 18,4 | ° | ° | ° |
| 4 | 4+4 | 2 | 1 | 2 | 0 (sn) | 0 | ° | ° | 1 | 0 | 4 | 90 | 0 | 10 | 3+ | ° | 1a | 0 | 0 | 0 | 1 | 0 | 0 | ° | 18,4 | ° | ° | ° |
| 4 | 12 | 2 | 1 | 1 | 1(sn) | 1 | 12 | 0 | 1 | 0 | 12 | 90 | 70 | 10 | 2+ | 0 | 1c | 1a | 0 | 0 | 0 | 1 | 0 | ° | 18,2 | ° | ° | ° |
| 4 | 4+4 | 2 | 1 | 4 | 0 (sn) | 0 | ° | ° | 1 | 0 | 0 | ° | ° | ° | ° | ° | is | 0 | 0 | 0 | 1 | 1 | 0 | ° | 17,8 | ° | ° | ° |
| 12 | 12 | 2 | 1 | 2 | 0 (sn) | 0 | ° | ° | 1 | 1 | 33 | 90 | 90 | 10 | 2+ | 1 | 2 | 0 | 0 | 0 | 1 | 1 | 0 | ° | 17,3 | ° | ° | ° |
| 4 | 12 | 1 | 1 | 3 | 1(sn) | 1 | 15 | 10 | 1 | 0 | 15 | 70 | 50 | 7 | 0/1+ | ° | 1c | 3a | 1 | 0 | 0 | 1 | 0 | ° | 16,6 | ° | ° | ° |
| 4 | 12 | 1 | 1 | 1 | 0 (sn) | 0 | ° | ° | 1 | 1 | 22 | 0 | 0 | 80 | 0/1+ | ° | 2 | 0 | 1 | 1 | 0 | 0 | 0 | ° | 15,9 | ° | ° | ° |
| 4 | 4+4 | 1 | 1 | 2 | 0 (sn) | 0 | ° | ° | 1 | 1 | 3 | 0 | 0 | 20 | 3+ | ° | 1a | 0 | 1 | 0 | 1 | 0 | 0 | ° | 15,8 | ° | ° | ° |
| 4 | 12 | 2 | 1 | 3 | 0 (sn) | 0 | ° | ° | 1 | 0 | ° | ° | ° | ° | ° | 0 | 0 | 0 | 0 | 0 | 0 | 0 | 0 | ° | 15,6 | ° | ° | ° |
| 4 | 4+4 | 1 | 1 | 4 | 0 (sn) | 0 | ° | ° | 1 | 0 | ° | ° | ° | ° | ° | 0 | 0 | 0 | 1 | 0 | 1 | 0 | 0 | ° | 15,5 | ° | ° | ° |
| 4 | 4+4 | 1 | 1 | 3 | 0 (sn) | 0 | ° | ° | 1 | 0 | 8 | 80 | 10 | 20 | 3+ | ° | 1b | 0 | 1 | 0 | 1 | 1 | 0 | ° | 15,5 | ° | ° | ° |
| 4 | 12 | 2 | 1 | 3 | 0 (sn) | 0 | ° | ° | 1 | 0 | ° | ° | ° | ° | ° | 0 | 0 | 0 | 0 | 0 | 0 | 0 | 0 | ° | 15,3 | ° | ° | ° |
| 4 | 4+4 | 1 | 1 | 1 | 0 (sn) | 0 | ° | ° | 1 | 0 | 0 | ° | ° | ° | ° | ° | is | 0 | 1 | 0 | 1 | 0 | 0 | ° | 15,2 | ° | ° | ° |
| 4 | 12+12 | 1 | 1 | 1 | 0 (sn) | 0 | ° | ° | 1 | 0 | 0 | ° | ° | ° | ° | ° | is | 0 | 1 | 0 | 1 | 0 | 0 | ° | 15,2 | ° | ° | ° |
| 4 | 12 | 2 | 1 | 2 | 0 (sn) | 0 | ° | ° | 1 | 0 | ° | ° | ° | ° | ° | 0 | 0 | 0 | 0 | 0 | 0 | 0 | 1 | 15/05/2022 | 8,2 | 2 | ° | ° |
| 4 | 12 | 1 | 1 | 4 | 1(sn) | 1 | 15 | 5 | 0 | 0 | 6 | 80 | 90 | 20 | 0/1+ | ° | 1b | 2a | 1 | 0 | 0 | 1 | 0 | ° | 10,3 | ° | ° | ° |
| 4 | 12+12 | 1 | 1 | 3 | 0 (sn) | 0 | ° | ° | 1 | 0 | 15 | 0 | 0 | 10 | 3+ | ° | 1c | 0 | 1 | 0 | 1 | 0 | 0 | ° | 14,6 | ° | ° | ° |

| | | | | | | | | | | | | | | | | | | | | | | | | | | | | |
|---|---|---|---|---|---|---|---|---|---|---|---|---|---|---|---|---|---|---|---|---|---|---|---|---|---|---|---|---|
| 4 | 4+4 | 2 | 1 | 6 | 0 (sn) | 0 | ° | ° | 1 | 0 | 10 | 90 | 10 | 35 | 3+ | ° | 1b | 0 | 0 | 0 | 1 | 1 | 0 | ° | 17,1 | ° | ° | ° |
| 4 | 4 | 2 | 1 | 3 | 0 (sn) | 0 | ° | ° | 1 | 0 | 24 | 15 | 1 | 70 | 0/1+ | ° | 2 | 0 | 0 | 0 | 0 | 1 | 0 | ° | 17,1 | ° | ° | ° |
| 4 | 4 | 2 | 1 | 2 | 0 (sn) | 0 | ° | ° | 1 | 0 | 16 | 0 | 0 | 25 | 2+ | 1 | 1c | 0 | 0 | 0 | 1 | 0 | 0 | ° | 16,9 | ° | ° | ° |
| 4 | 12+12 | 1 | 1 | 1 | 1(sn) | 1 | 11 | 0 | 1 | 1 | 14 | 90 | 90 | 10 | 3+ | ° | 1c | 0 | 1 | 0 | 1 | 1 | 0 | ° | 16,8 | ° | ° | ° |
| 4 | 4+4 | 2 | 1 | 3 | 0 (sn) | 0 | ° | ° | 1 | 0 | ° | ° | ° | ° | ° | 0 | 0 | 0 | 0 | 0 | 1 | 0 | 0 | ° | 13,8 | ° | ° | ° |
| 4 | 12+12 | 2 | 1 | 2 | 1(sn) | 0 | ° | ° | 1 | 0 | 6 | 60 | 0 | 25 | 3+ | ° | 1b | 1a | 1 | 0 | 1 | 1 | 0 | ° | 13,8 | ° | ° | ° |
| 4 | 6 | 1 | 1 | 5 | 0 (sn) | 0 | ° | ° | 1 | 0 | ° | ° | ° | ° | ° | 0 | 0 | 0 | 1 | 0 | 0 | 0 | 0 | ° | 13,1 | ° | ° | ° |
| 4 | 12+12 | 2 | 1 | 3 | 0 (sn) | 0 | ° | ° | 1 | 0 | 8 | 80 | 1 | 10 | 3+ | ° | 1b | 0 | 0 | 0 | 1 | 1 | 0 | ° | 12,9 | ° | ° | ° |
| 4 | 12+12 | 1 | 1 | 1 | 0 (sn) | 0 | ° | ° | 1 | 0 | ° | ° | ° | ° | ° | 0 | 0 | 0 | 1 | 0 | 1 | 0 | 0 | ° | 12,2 | ° | ° | ° |
| | | | | | | | | | | | | | | | | | | | | | | | | | | | | |

| Data metastasi | DDFS | Tipo metastasi | Trattamento metastasi | Morte | Data morte | OS | Causa morte | Ultimo follow-up | Perso follow-up | note | |
|---|---|---|---|---|---|---|---|---|---|---|---|
| ° | 43,7 | ° | ° | 0 | ° | 43,7 | ° | 09/12/2022 | 0 | | Criteri di esclusione: progressione di malattia, pregresso intervento per K |
| ° | 159,1 | ° | ° | 0 | ° | 159,1 | ° | 09/12/2022 | 0 | | |
| 06/10/2017 | 23,8 | 1, 3, 5 | CT, BT | 0 | ° | 69,3 | ° | 02/07/2021 | 0 | da chiamare. 3333922972, non risponde | Balocco |
| ° | 65,9 | ° | ° | 0 | ° | 65,9 | ° | 09/12/2022 | 0 | | |
| ° | 41,9 | ° | ° | 0 | ° | 41,9 | ° | 09/12/2022 | 0 | | |
| 18/04/2019 | 13,1 | 1, 3, 5 | nessuno | 1 | 20/05/2019 | 14,2 | 1 | 20/05/2019 | 0 | | |
| ° | 85,0 | ° | ° | 0 | ° | 85,0 | ° | 09/12/2022 | 0 | | |
| ° | 97,9 | ° | ° | 0 | ° | 97,9 | ° | 09/12/2022 | 0 | | |
| ° | 128,6 | ° | ° | 0 | ° | 128,6 | ° | 09/12/2022 | 0 | | |
| 03/03/2021 | 12,3 | 4 | nessuno | 0 | ° | 21,7 | ° | 09/12/2021 | 0 | siss | |
| 15/12/2015 | 82,6 | 2, 3 | CT, HT | 1 | 06/02/2019 | 120,9 | 1 | 06/02/2019 | 0 | | |
| 14/06/2019 | 15,0 | 1, 2, 3, 4 | nessuno | 1 | 17/07/2019 | 16,1 | 1 | 17/07/2019 | 0 | | |
| ° | 49,0 | ° | ° | 0 | ° | 49,0 | ° | 09/12/2022 | 0 | | |
| ° | 26,8 | ° | ° | 0 | ° | 26,8 | ° | 09/12/2021 | 0 | siss | |
| ° | 20,0 | ° | ° | 1 | 27/07/2020 | 20,0 | 0 | 27/07/2020 | 0 | | |
| ° | 35,7 | ° | ° | 0 | ° | 35,7 | ° | 01/04/2019 | 1 | da chiamare? . 3409625579 -3275981260, non risponde | |
| ° | 102,9 | ° | ° | 0 | ° | 102,9 | ° | 09/12/2022 | 0 | | |
| ° | 43,4 | ° | ° | 0 | ° | 43,4 | ° | 09/12/2022 | 0 | | |
| 08/08/2022 | 161,1 | 1,2 | nessuno | 0 | ° | 165,2 | ° | 09/12/2022 | 0 | | |
| 30/09/2022 | 46,2 | 5 | nessuno | 0 | ° | 48,533 | ° | 09/12/2022 | 0 | | |
| ° | 132,9 | ° | ° | 1 | 20/10/2019 | 132,9 | 0 | 20/10/2019 | 0 | | |
| ° | 157,2 | ° | ° | 0 | ° | 157,2 | ° | 09/12/2021 | 0 | siss | |
| ° | 169,4 | ° | ° | 0 | ° | 169,4 | ° | 09/12/2022 | 0 | | |
| ° | 156,6 | ° | ° | 0 | ° | 156,6 | ° | 09/12/2021 | 0 | siss | |
| ° | 167,8 | ° | ° | 0 | ° | 167,8 | ° | 09/12/2022 | 0 | | |
| 15/09/2016 | 85,5 | 1, 2, 3, 6 | CT, RT, HT | 1 | 29/09/2017 | 98,1 | 1 | 29/09/2017 | 0 | | |
| ° | 46,0 | ° | ° | 0 | ° | 46,0 | ° | 09/12/2022 | 0 | | |
| ° | 159,0 | ° | ° | 0 | ° | 159,0 | ° | 09/12/2022 | 0 | | |
| ° | 61,5 | ° | ° | 0 | ° | 61,5 | ° | 20/02/2015 | 1 | Da chiamare? 3355964368, non risponde | |
| ° | 158,5 | ° | ° | 0 | ° | 158,5 | ° | 09/12/2022 | 0 | | |
| ° | 137,4 | ° | ° | 0 | ° | 137,4 | ° | 02/07/2021 | 0 | da chiamare 3293888140, chiamata ultimo f.u. 2 aa fa | |
| ° | 46,9 | ° | ° | 0 | ° | 46,9 | ° | 09/12/2022 | 0 | | |
| ° | 51,9 | ° | ° | 0 | ° | 51,9 | ° | 09/12/2022 | 0 | | |
| ° | 143,8 | ° | ° | 0 | ° | 143,8 | ° | 09/12/2022 | 0 | | |
| ° | 39,5 | ° | ° | 0 | ° | 39,5 | ° | 09/12/2022 | 0 | | |
| ° | 121,4 | ° | ° | 0 | ° | 121,4 | ° | 09/12/2021 | 0 | siss | |
| ° | 34,6 | ° | ° | 0 | ° | 34,6 | ° | 09/12/2021 | 0 | siss | |
| 07/08/2018 | 30,1 | 1, 2, 3, 4, 5 | CT, RT, CHIR | 1 | 11/05/2021 | 63,7 | 1 | 11/05/2021 | 0 | | |
| ° | 75,4 | ° | ° | 0 | ° | 75,4 | ° | 09/12/2022 | 0 | | |

| | | | | | | | | | | | |
|---|---|---|---|---|---|---|---|---|---|---|---|
| ° | 111,6 | ° | ° | 0 | ° | 111,6 | ° | 09/12/2022 | 0 | | |
| ° | 106,5 | ° | ° | 0 | ° | 106,5 | ° | 09/12/2021 | 0 | siss | |
| ° | 109,2 | ° | ° | 0 | ° | 109,2 | ° | 09/12/2022 | 0 | | |
| ° | 10,1 | ° | ° | 1 | 22/03/2014 | 10,1 | 0 | 22/03/2014 | 0 | | |
| ° | 96,4 | ° | ° | 0 | ° | 96,4 | ° | 09/12/2021 | 0 | siss | |
| ° | 57,9 | ° | ° | 0 | ° | 57,9 | ° | 09/12/2022 | 0 | | |
| ° | 103,6 | ° | ° | 0 | ° | 103,6 | ° | 09/12/2022 | 0 | | |
| ° | 104,9 | ° | ° | 0 | ° | 104,9 | ° | 09/12/2022 | 0 | | |
| 16/09/2021 | 50,6 | 1,2,3,5 | CT | 1 | 24/10/2021 | 51,9 | 1 | 24/10/2021 | 0 | morta | |
| ° | 39,8 | ° | ° | 0 | ° | 39,8 | ° | 12/01/2018 | 1 | non ce recapito, | |
| ° | 86,8 | ° | ° | 0 | ° | 86,8 | ° | 02/07/2021 | 0 | da chiamare, 3342135637, non risponde | |
| ° | 83,4 | ° | ° | 0 | ° | 83,4 | ° | 02/07/2021 | 0 | da chiamare, 3333463373,non risponde | |
| ° | 81,3 | ° | CHIR, TB | 0 | ° | 81,3 | ° | 02/07/2021 | 0 | da chiamare 3470084574, non risponde | |
| ° | 86,8 | ° | ° | 0 | ° | 86,8 | ° | 02/07/2021 | 0 | da chiamare, 3473857437, non risponde | |
| ° | 1,4 | ° | ° | 0 | ° | 1,4 | ° | 10/06/2014 | 1 | niente recapito | |
| ° | 97,9 | ° | ° | 0 | ° | 97,9 | ° | 09/12/2022 | 0 | | |
| ° | 103,1 | ° | ° | 0 | ° | 103,1 | ° | 09/12/2022 | 0 | | |
| ° | 85,4 | ° | ° | 0 | ° | 85,4 | ° | 02/07/2021 | 0 | da chiamare 3391523752, non risponde | |
| ° | 95,3 | ° | ° | 0 | ° | 95,3 | ° | 09/12/2022 | 0 | | |
| 26/11/2019 | 2,8 | 2, 3, 5 | CT, RT, CHIR | 1 | 15/07/2020 | 10,5 | 1 | 15/07/2020 | 0 | | |
| ° | 97,4 | ° | ° | 0 | ° | 97,4 | ° | 09/12/2022 | 0 | | |
| ° | 93,4 | ° | ° | 0 | ° | 93,4 | ° | 09/12/2022 | 0 | | |
| ° | 93,1 | ° | ° | 0 | ° | 93,1 | ° | 09/12/2022 | 0 | | |
| ° | 79,1 | ° | ° | 0 | ° | 79,1 | ° | 09/12/2021 | 0 | siss | |
| ° | 93,1 | ° | ° | 0 | ° | 93,1 | ° | 09/12/2022 | 0 | | |
| ° | 39,9 | ° | ° | 1 | 16/08/2018 | 39,9 | 0 | 16/08/2018 | 0 | | |
| 09/02/2018 | 32,5 | 1, 4 | CHIR, CT | 1 | 27/07/2019 | 50,2 | 1 | 27/07/2019 | 0 | | |
| ° | 70,8 | ° | ° | 0 | ° | 70,8 | ° | 02/07/2021 | 0 | | |
| ° | 8,3 | ° | ° | 1 | 22/05/2016 | 8,3 | 0 | 22/05/2016 | 0 | | |
| ° | 20,6 | ° | ° | 0 | ° | 20,6 | ° | 02/07/2021 | 0 | da chiamare 3487291987, non risponde | |
| 18/01/2017 | 18,2 | 1 | CT | 1 | 01/07/2017 | 23,7 | 1 | 01/07/2017 | 0 | | |
| ° | 52,7 | ° | ° | 1 | 15/12/2019 | 52,7 | 0 | 15/12/2019 | 0 | | |
| ° | 0,5 | ° | ° | 0 | ° | 0,5 | ° | 19/08/2015 | 1 | da chiamare 33319250015-3425478741, non risponde | |
| ° | 34,6 | ° | ° | 0 | ° | 34,6 | ° | 09/12/2022 | 0 | | |
| ° | 94,3 | ° | ° | 0 | ° | 94,3 | ° | 09/12/2022 | 0 | | |
| ° | 86,4 | ° | ° | 0 | ° | 86,4 | ° | 09/12/2022 | 0 | | |
| ° | 30,0 | ° | ° | 0 | ° | 30,0 | ° | 28/05/2018 | 1 | | |
| ° | 83,6 | ° | ° | 0 | ° | 83,6 | ° | 09/12/2022 | 0 | | |
| 25/05/2020 | 51,3 | 1, 2, 3, 5 | CT, CHIR | 1 | 25/04/2021 | 62,5 | 1 | 25/04/2021 | 0 | | |
| ° | 83,1 | ° | ° | 0 | ° | 83,1 | ° | 09/12/2022 | 0 | | |
| ° | 82,9 | ° | ° | 0 | ° | 82,9 | ° | 09/12/2022 | 0 | | |
| ° | 83,3 | ° | ° | 0 | ° | 83,3 | ° | 09/12/2022 | 0 | | |

| | | | | | | | | | | | |
|---|---|---|---|---|---|---|---|---|---|---|---|
| ° | 82,0 | ° | ° | 0 | ° | 82,0 | ° | 09/12/2022 | 0 | | |
| ° | 62,8 | ° | ° | 0 | ° | 62,8 | ° | 02/07/2021 | 0 | da chiamare 3389800855, non risponde | |
| ° | 69,4 | ° | ° | 0 | ° | 69,4 | ° | 02/07/2021 | 0 | da chiamare, 0124519381-3805128242, non risponde | |
| ° | 86,7 | ° | ° | 0 | ° | 86,7 | ° | 09/12/2022 | 0 | | |
| ° | 76,2 | ° | ° | 0 | ° | 76,2 | ° | 09/12/2022 | 0 | | |
| ° | 76,1 | ° | ° | 0 | ° | 76,1 | ° | 09/12/2022 | 0 | | |
| ° | 15,0 | ° | ° | 0 | ° | 15,0 | ° | 06/10/2017 | 1 | da chiamare? 0185295871, non risponde | |
| ° | 83,8 | ° | ° | 0 | ° | 83,8 | ° | 09/12/2022 | 0 | | |
| ° | 73,4 | ° | ° | 0 | ° | 73,4 | ° | 09/12/2022 | 0 | | |
| ° | 54,5 | ° | ° | 0 | ° | 54,5 | ° | 02/07/2021 | 0 | da chiamare 3889747136, non risponde | |
| 01/06/2020 | 48,4 | 1,2,4 | CT | 0 | ° | 79,1 | ° | 09/12/2022 | 0 | | |
| ° | 71,8 | ° | ° | 0 | ° | 71,8 | ° | 09/12/2022 | 0 | | |
| ° | 71,4 | ° | ° | 0 | ° | 71,4 | ° | 09/12/2022 | 0 | | |
| ° | 7,8 | ° | ° | 0 | ° | 7,8 | ° | 25/09/2017 | 1 | da chiamare 3402223962, non risponde | |
| ° | 51,7 | ° | ° | 1 | 15/04/2021 | 51,7 | 0 | 15/04/2021 | 0 | | |
| ° | 55,6 | ° | ° | 0 | ° | 55,6 | ° | 02/07/2021 | 0 | da chiamare, 3392228648, non risponde | |
| ° | 35,6 | ° | ° | 0 | ° | 35,6 | ° | 17/06/2019 | 1 | da chiamare? 3475027887, non risponde | |
| ° | 77,5 | ° | ° | 0 | ° | 77,5 | ° | 09/12/2022 | 0 | | |
| ° | 58,1 | ° | ° | 0 | ° | 58,1 | ° | 09/12/2021 | 0 | siss | |
| ° | 75,7 | ° | ° | 0 | ° | 75,7 | ° | 09/12/2022 | 0 | | |
| ° | 69,5 | ° | ° | 0 | ° | 69,5 | ° | 09/12/2022 | 0 | | |
| ° | 69,6 | ° | ° | 0 | ° | 69,6 | ° | 09/12/2022 | 0 | | |
| ° | 68,7 | ° | ° | 0 | ° | 68,7 | ° | 09/12/2022 | 0 | | |
| ° | 53,9 | ° | ° | 0 | ° | 53,9 | ° | 02/07/2021 | 0 | da chiamare 3473306813, non risponde | |
| ° | 65,0 | ° | ° | 0 | ° | 65,0 | ° | 09/12/2022 | 0 | | |
| ° | 73,5 | ° | ° | 0 | ° | 73,5 | ° | 09/12/2022 | 0 | | |
| 30/05/2019 | 22,6 | 3, 4 | CT | 1 | 03/06/2020 | 34,9 | 1 | 03/06/2020 | 0 | | |
| ° | 57,0 | ° | ° | 0 | ° | 57,0 | ° | 09/12/2022 | 0 | | |
| ° | 72,8 | ° | ° | 0 | ° | 72,8 | ° | 09/12/2022 | 0 | | |
| ° | 65,0 | ° | ° | 0 | ° | 65,0 | ° | 09/12/2022 | 0 | | |
| ° | 63,1 | ° | ° | 0 | ° | 63,1 | ° | 09/12/2022 | 0 | | |
| ° | 65,2 | ° | ° | 0 | ° | 65,2 | ° | 09/12/2022 | 0 | | |
| ° | 63,3 | ° | ° | 0 | ° | 63,3 | ° | 09/12/2022 | 0 | | |
| ° | 57,9 | ° | ° | 0 | ° | 57,9 | ° | 09/12/2021 | 0 | siss | |
| ° | 66,8 | ° | ° | 0 | ° | 66,8 | ° | 09/12/2022 | 0 | | |
| ° | 66,0 | ° | ° | 0 | ° | 66,0 | ° | 09/12/2022 | 0 | | |
| ° | 53,3 | ° | ° | 0 | ° | 53,3 | ° | 09/12/2021 | 0 | siss | |
| ° | 53,4 | ° | ° | 0 | ° | 53,4 | ° | 09/12/2021 | 0 | siss | |
| ° | 51,4 | ° | ° | 0 | ° | 51,4 | ° | 09/12/2021 | 0 | siss | |
| ° | 43,9 | ° | ° | 0 | ° | 43,9 | ° | 02/07/2021 | 0 | da chiamare, 3473324008, non risponde | |
| ° | 60,2 | ° | ° | 0 | ° | 60,2 | ° | 09/12/2022 | 0 | | |
| ° | 56,0 | ° | ° | 0 | ° | 56,0 | ° | 09/12/2022 | 0 | | |

| | | | | | | | | | | | |
|---|---|---|---|---|---|---|---|---|---|---|---|
| ° | 45,6 | ° | ° | 0 | ° | 45,6 | ° | 09/12/2022 | 0 | | |
| ° | 34,6 | ° | ° | 0 | ° | 34,6 | ° | 02/07/2021 | 0 | da chiamare, 3475043239, non risponde | |
| ° | 49,5 | ° | ° | 0 | ° | 49,5 | ° | 09/12/2022 | 0 | | |
| ° | 4,8 | ° | ° | 0 | ° | 4,8 | ° | 22/07/2019 | 1 | | |
| ° | 37,7 | ° | ° | 0 | ° | 37,7 | ° | 09/12/2022 | 0 | | |
| ° | 45,4 | ° | ° | 0 | ° | 45,4 | ° | 09/12/2022 | 0 | | |
| ° | 3,2 | ° | ° | 0 | ° | 3,2 | ° | 25/07/2019 | 1 | | |
| ° | 42,2 | ° | ° | 0 | ° | 42,2 | ° | 09/12/2022 | 0 | | |
| ° | 42,0 | ° | ° | 0 | ° | 42,0 | ° | 09/12/2022 | 0 | | |
| ° | 2,0 | ° | ° | 0 | ° | 2,0 | ° | 18/09/2019 | 1 | | |
| ° | 0,9 | ° | ° | 0 | ° | 0,9 | ° | 11/10/2019 | 1 | | |
| ° | 34,5 | ° | ° | 0 | ° | 34,5 | ° | 09/12/2022 | 0 | | |
| ° | 33,4 | ° | ° | 0 | ° | 33,4 | ° | 09/12/2022 | 0 | | |
| 10/10/2022 | 31,5 | 5 | CHIR | 0 | ° | 33,5 | ° | 09/12/2022 | 0 | | |
| ° | 63,3 | ° | ° | 0 | ° | 63,3 | ° | 09/12/2022 | 0 | | |
| ° | 61,2 | ° | ° | 0 | ° | 61,2 | ° | 09/12/2022 | 0 | chiamata | |
| ° | 59,5 | ° | ° | 0 | ° | 59,5 | ° | 09/12/2022 | 0 | chiamata | |
| ° | 66,3 | ° | ° | 0 | ° | 66,3 | ° | 09/12/2022 | 0 | chiamata | |
| ° | 59,6 | ° | ° | 0 | ° | 59,6 | ° | 09/12/2022 | 0 | | |
| ° | 60,7 | ° | ° | 0 | ° | 60,7 | ° | 09/12/2022 | 0 | | |
| ° | 40,4 | ° | ° | 0 | ° | 40,4 | ° | 02/07/2021 | 0 | | |
| ° | 56,7 | ° | ° | 0 | ° | 56,7 | ° | 09/12/2022 | 0 | | |
| ° | 54,4 | ° | ° | 0 | ° | 54,4 | ° | 09/12/2022 | 0 | | |
| ° | 55,0 | ° | ° | 0 | ° | 55,0 | ° | 09/12/2022 | 0 | | |
| ° | 55,3 | ° | ° | 0 | ° | 55,3 | ° | 09/12/2022 | 0 | | |
| ° | 53,2 | ° | ° | 0 | ° | 53,2 | ° | 09/12/2022 | 0 | | |
| ° | 55,3 | ° | ° | 0 | ° | 55,3 | ° | 09/12/2022 | 0 | | |
| 15/09/2019 | 14,9 | 2, 5 | CT, RT | 1 | 21/02/2020 | 20,2 | 1 | 21/02/2020 | 0 | | |
| ° | 53,8 | ° | ° | 0 | ° | 53,8 | ° | 09/12/2022 | 0 | | |
| ° | 53,5 | ° | ° | 0 | ° | 53,5 | ° | 09/12/2022 | 0 | | |
| ° | 36,3 | ° | ° | 0 | ° | 36,3 | ° | 09/12/2022 | 0 | | |
| ° | 55,4 | ° | ° | 0 | ° | 55,4 | ° | 09/12/2022 | 0 | | |
| ° | 12,4 | ° | ° | 0 | ° | 12,4 | ° | 18/06/2019 | 1 | | |
| ° | 53,0 | ° | ° | 0 | ° | 53,0 | ° | 09/12/2022 | 0 | | |
| ° | 53,3 | ° | ° | 0 | ° | 53,3 | ° | 09/12/2022 | 0 | | |
| ° | 59,9 | ° | ° | 0 | ° | 59,9 | ° | 09/12/2022 | 0 | | |
| ° | 51,7 | ° | ° | 0 | ° | 51,7 | ° | 09/12/2022 | 0 | | |
| ° | 55,1 | ° | ° | 0 | ° | 55,1 | ° | 09/12/2022 | 0 | | |
| ° | 51,9 | ° | ° | 0 | ° | 51,9 | ° | 09/12/2022 | 0 | chiamata | |
| ° | 51,6 | ° | ° | 0 | ° | 51,6 | ° | 09/12/2022 | 0 | chiamata | |
| ° | 53,3 | ° | ° | 0 | ° | 53,3 | ° | 09/12/2022 | 0 | | |
| ° | 51,2 | ° | ° | 0 | ° | 51,2 | ° | 09/12/2022 | 0 | | |

| | | | | | | | | | | | |
|---|---|---|---|---|---|---|---|---|---|---|---|
| ° | 35,8 | ° | ° | 0 | ° | 35,8 | ° | 02/07/2021 | 0 | da chiamare, 3807922072, non risponde (neanche al 2 tentativo) | |
| ° | 50,9 | ° | ° | 0 | ° | 50,9 | ° | 09/12/2022 | 0 | | |
| ° | 53,8 | ° | ° | 0 | ° | 53,8 | ° | 09/12/2022 | 0 | | |
| ° | 51,4 | ° | ° | 0 | ° | 51,4 | ° | 09/12/2022 | 0 | | |
| ° | 49,8 | ° | ° | 0 | ° | 49,8 | ° | 09/12/2022 | 0 | | |
| ° | 32,5 | ° | ° | 0 | ° | 32,5 | ° | 02/07/2021 | 0 | da chiamare, 3497627914, non risponde (neanche a al 2 tentativo) | |
| ° | 55,8 | ° | ° | 0 | ° | 55,8 | ° | 09/12/2022 | 0 | | |
| ° | 49,0 | ° | ° | 0 | ° | 49,0 | ° | 09/12/2022 | 0 | | |
| ° | 48,6 | ° | ° | 0 | ° | 48,6 | ° | 09/12/2022 | 0 | | |
| ° | 45,1 | ° | ° | 0 | ° | 45,1 | ° | 09/12/2022 | 0 | | |
| ° | 46,5 | ° | ° | 0 | ° | 46,5 | ° | 09/12/2022 | 0 | | |
| ° | 46,5 | ° | ° | 0 | ° | 46,5 | ° | 09/12/2022 | 0 | | |
| ° | 44,3 | ° | ° | 0 | ° | 44,3 | ° | 09/12/2022 | 0 | | |
| ° | 0,5 | ° | ° | 0 | ° | 0,5 | ° | 13/06/2019 | 1 | | |
| ° | 43,7 | ° | ° | 0 | ° | 43,7 | ° | 09/12/2022 | 0 | | |
| ° | 44,4 | ° | ° | 0 | ° | 44,4 | ° | 09/12/2022 | 0 | | |
| ° | 45,8 | ° | ° | 0 | ° | 45,8 | ° | 09/12/2022 | 0 | | |
| ° | 44,6 | ° | ° | 0 | ° | 44,6 | ° | 09/12/2022 | 0 | | |
| ° | 48,5 | ° | ° | 0 | ° | 48,5 | ° | 09/12/2022 | 0 | chiamata | |
| 15/01/2021 | 22,7 | 2 | CT | 0 | ° | 28,3 | ° | 02/07/2021 | 0 | da chiamare, 3349456566, non risponde (non squilla) | |
| ° | 49,6 | ° | ° | 0 | ° | 49,6 | ° | 09/12/2022 | 0 | | |
| ° | 40,0 | ° | ° | 0 | ° | 40,0 | ° | 09/12/2022 | 0 | | |
| ° | 29,4 | ° | ° | 0 | ° | 29,4 | ° | 02/07/2021 | 0 | da chiamare 3494290658, non risponde (non ragguingibile) | |
| 15/07/2022 | 34,4 | 2, 4, 5 | CHT, RT | 0 | ° | 39,3 | ° | 09/12/2022 | 0 | chiamata | |
| ° | 46,9 | ° | ° | 0 | ° | 46,9 | ° | 09/12/2022 | 0 | | |
| ° | 46,2 | ° | ° | 0 | ° | 46,2 | ° | 09/12/2022 | 0 | | |
| 01/06/2021 | 21,4 | 2, 3 | CHT, RT | 0 | ° | 39,9 | ° | 09/12/2022 | 0 | chiamata | |
| ° | 40,8 | ° | ° | 0 | ° | 40,8 | ° | 09/12/2022 | 0 | chiamata | |
| ° | 39,5 | ° | ° | 0 | ° | 39,5 | ° | 09/12/2022 | 0 | | |
| 09/12/2021 | 24,6 | 1, 2, 3, 4, 5 | CT, RT | 0 | ° | 36,733 | ° | 09/12/2022 | 0 | | |
| ° | 37,9 | ° | ° | 0 | ° | 37,9 | ° | 09/12/2022 | 0 | | |
| ° | 44,8 | ° | ° | 0 | ° | 44,8 | ° | 09/12/2022 | 0 | | |
| ° | 37,3 | ° | ° | 0 | ° | 37,3 | ° | 09/12/2022 | 0 | | |
| ° | 35,1 | ° | ° | 0 | ° | 35,1 | ° | 09/12/2022 | 0 | chiamata | Aiello 1978 |
| ° | 38,8 | ° | ° | 0 | ° | 38,8 | ° | 09/12/2022 | 0 | | |
| ° | 35,3 | ° | ° | 0 | ° | 35,3 | ° | 09/12/2022 | 0 | | |
| ° | 35,5 | ° | ° | 0 | ° | 35,5 | ° | 09/12/2022 | 0 | | |
| ° | 33,6 | ° | ° | 0 | ° | 33,6 | ° | 09/12/2022 | 0 | chiamata | Gilardelli 1951 |
| 02/10/2020 | 10,3 | 4 | HT | 1 | 31/07/2021 | 19,4 | 1 | 02/07/2021 | 0 | morta | Zamfirescu 1986 |
| ° | 32,1 | ° | ° | 0 | ° | 32,1 | ° | 09/12/2022 | 0 | | |
| ° | 35,3 | ° | ° | 0 | ° | 35,3 | ° | 09/12/2022 | 0 | chiamata | Gagliardelli 1960 |
| ° | 34,3 | ° | ° | 0 | ° | 34,3 | ° | 09/12/2022 | 0 | chiamata | Alberti 1972 |

| | | | | | | | | | | | |
|---|---|---|---|---|---|---|---|---|---|---|---|
| ° | 31,5 | ° | ° | 0 | ° | 31,5 | ° | 09/12/2022 | 0 | | |
| ° | 31,5 | ° | ° | 0 | ° | 31,5 | ° | 09/12/2022 | 0 | | |
| ° | 31,4 | ° | ° | 0 | ° | 31,4 | ° | 09/12/2022 | 0 | | |
| ° | 31,3 | ° | ° | 0 | ° | 31,3 | ° | 09/12/2022 | 0 | | |
| ° | 31,1 | ° | ° | 0 | ° | 31,1 | ° | 09/12/2022 | 0 | chiamata | |
| ° | 31,1 | ° | ° | 0 | ° | 31,1 | ° | 09/12/2022 | 0 | | |
| ° | 31,0 | ° | ° | 0 | ° | 31,0 | ° | 09/12/2022 | 0 | | |
| ° | 30,8 | ° | ° | 0 | ° | 30,8 | ° | 09/12/2022 | 0 | chiamata | |
| ° | 30,8 | ° | ° | 0 | ° | 30,8 | ° | 09/12/2022 | 0 | chiamata | |
| ° | 30,6 | ° | ° | 0 | ° | 30,6 | ° | 09/12/2022 | 0 | | |
| ° | 30,6 | ° | ° | 0 | ° | 30,6 | ° | 09/12/2022 | 0 | | |
| ° | 30,4 | ° | ° | 0 | ° | 30,4 | ° | 09/12/2022 | 0 | chiamata | |
| ° | 30,4 | ° | ° | 0 | ° | 30,4 | ° | 09/12/2022 | 0 | chiamata | |
| ° | 30,3 | ° | ° | 0 | ° | 30,3 | ° | 09/12/2022 | 0 | | |
| ° | 30,2 | ° | ° | 0 | ° | 30,2 | ° | 09/12/2022 | 0 | | |
| ° | 30,2 | ° | ° | 0 | ° | 30,2 | ° | 09/12/2022 | 0 | | |
| ° | 30,1 | ° | ° | 0 | ° | 30,1 | ° | 09/12/2022 | 0 | | |
| ° | 29,9 | ° | ° | 0 | ° | 29,9 | ° | 09/12/2022 | 0 | | |
| ° | 29,7 | ° | ° | 0 | ° | 29,7 | ° | 09/12/2022 | 0 | | |
| ° | 29,7 | ° | ° | 0 | ° | 29,7 | ° | 09/12/2022 | 0 | | |
| ° | 29,6 | ° | ° | 0 | ° | 29,6 | ° | 09/12/2022 | 0 | | |
| ° | 29,3 | ° | ° | 0 | ° | 29,3 | ° | 09/12/2022 | 0 | | |
| ° | 28,9 | ° | ° | 0 | ° | 28,9 | ° | 09/12/2022 | 0 | | |
| ° | 28,8 | ° | ° | 0 | ° | 28,8 | ° | 09/12/2022 | 0 | | |
| ° | 28,6 | ° | ° | 0 | ° | 28,6 | ° | 09/12/2022 | 0 | | |
| ° | 28,6 | ° | ° | 0 | ° | 28,6 | ° | 09/12/2022 | 0 | | |
| 02/10/2020 | 1,9 | 5 | RT, CHT | 0 | ° | 28,5 | ° | 09/12/2022 | 0 | | |
| 16/05/2022 | 21,4 | 1, 5 | RT, CHT | 0 | ° | 28,3 | ° | 09/12/2022 | 0 | chiamata | Kuka 1983 |
| ° | 28,3 | ° | ° | 0 | ° | 28,3 | ° | 09/12/2022 | 0 | | |
| ° | 28,1 | ° | ° | 0 | ° | 28,1 | ° | 09/12/2022 | 0 | | |
| ° | 28,0 | ° | ° | 0 | ° | 28,0 | ° | 09/12/2022 | 0 | | |
| ° | 27,6 | ° | ° | 0 | ° | 27,6 | ° | 09/12/2022 | 0 | | |
| ° | 27,5 | ° | ° | 0 | ° | 27,5 | ° | 09/12/2022 | 0 | | |
| ° | 27,3 | ° | ° | 0 | ° | 27,3 | ° | 09/12/2022 | 0 | | |
| ° | 27,1 | ° | ° | 0 | ° | 27,1 | ° | 09/12/2022 | 0 | chiamata | Guerra 1960 |
| ° | 26,9 | ° | ° | 0 | ° | 26,9 | ° | 09/12/2022 | 0 | chiamata | Romeo 1977 |
| ° | 26,2 | ° | ° | 0 | ° | 26,2 | ° | 09/12/2022 | 0 | chiamata | Larocca 1970 |
| ° | 26,2 | ° | ° | 0 | ° | 26,2 | ° | 09/12/2022 | 0 | | |
| ° | 25,9 | ° | ° | 0 | ° | 25,9 | ° | 09/12/2022 | 0 | | |
| ° | 25,8 | ° | ° | 0 | ° | 25,8 | ° | 09/12/2022 | 0 | chiamata | Nacci 1963 |
| ° | 25,3 | ° | ° | 0 | ° | 25,3 | ° | 09/12/2022 | 0 | | |
| ° | 24,5 | ° | ° | 0 | ° | 24,5 | ° | 09/12/2022 | 0 | | |

| | | | | | | | | | | | |
|---|---|---|---|---|---|---|---|---|---|---|---|
| ° | 24,3 | ° | ° | 0 | ° | 24,3 | ° | 09/12/2022 | 0 | | |
| ° | 24,3 | ° | ° | 0 | ° | 24,3 | ° | 09/12/2022 | 0 | chiamata | Cammarata 1957 |
| ° | 24,1 | ° | ° | 0 | ° | 24,1 | ° | 09/12/2022 | 0 | | |
| ° | 23,9 | ° | ° | 0 | ° | 23,9 | ° | 09/12/2022 | 0 | | |
| ° | 23,7 | ° | ° | 0 | ° | 23,7 | ° | 09/12/2022 | 0 | | |
| ° | 23,4 | ° | ° | 0 | ° | 23,4 | ° | 09/12/2022 | 0 | | |
| ° | 22,5 | ° | ° | 0 | ° | 22,5 | ° | 09/12/2022 | 0 | chiamata | |
| ° | 22,4 | ° | ° | 0 | ° | 22,4 | ° | 09/12/2022 | 0 | | |
| ° | 22,3 | ° | ° | 0 | ° | 22,3 | ° | 09/12/2022 | 0 | chiamata | |
| ° | 22,0 | ° | ° | 0 | ° | 22,0 | ° | 09/12/2022 | 0 | | |
| 15/11/2021 | 9,0 | 3 | CHT | 0 | ° | 22,0 | ° | 09/12/2022 | 0 | chiamata | Sireni 1963 |
| ° | 22,0 | ° | ° | 0 | ° | 22,0 | ° | 09/12/2022 | 0 | chiamata | |
| ° | 21,6 | ° | ° | 0 | ° | 21,6 | ° | 09/12/2022 | 0 | | |
| ° | 21,6 | ° | ° | 0 | ° | 21,6 | ° | 09/12/2022 | 0 | | |
| ° | 21,5 | ° | ° | 0 | ° | 21,5 | ° | 09/12/2022 | 0 | chiamata | |
| ° | 21,3 | ° | ° | 0 | ° | 21,3 | ° | 09/12/2022 | 0 | | |
| 09/11/2021 | 7,9 | 2,3 | BT | 1 | 16/04/2022 | 13,2 | 1 | 16/04/2022 | 0 | morta | Sablone 1964 |
| ° | 20,9 | ° | ° | 0 | ° | 20,9 | ° | 09/12/2022 | 0 | | |
| ° | 20,3 | ° | ° | 0 | ° | 20,3 | ° | 09/12/2022 | 0 | | |
| ° | 20,1 | ° | ° | 0 | ° | 20,1 | ° | 09/12/2022 | 0 | | |
| ° | 19,9 | ° | ° | 0 | ° | 19,9 | ° | 09/12/2022 | 0 | | |
| ° | 19,6 | ° | ° | 0 | ° | 19,6 | ° | 09/12/2022 | 0 | chiamata | |
| ° | 19,2 | ° | ° | 0 | ° | 19,2 | ° | 09/12/2022 | 0 | | |
| ° | 19,1 | ° | ° | 0 | ° | 19,1 | ° | 09/12/2022 | 0 | | |
| ° | 18,7 | ° | ° | 0 | ° | 18,7 | ° | 09/12/2022 | 0 | | |
| ° | 18,4 | ° | ° | 0 | ° | 18,4 | ° | 09/12/2022 | 0 | | |
| ° | 18,4 | ° | ° | 0 | ° | 18,4 | ° | 09/12/2022 | 0 | | |
| ° | 18,2 | ° | ° | 0 | ° | 18,2 | ° | 09/12/2022 | 0 | | |
| ° | 17,8 | ° | ° | 0 | ° | 17,8 | ° | 09/12/2022 | 0 | | |
| ° | 17,3 | ° | ° | 0 | ° | 17,3 | ° | 09/12/2022 | 0 | | |
| ° | 16,6 | ° | ° | 0 | ° | 16,6 | ° | 09/12/2022 | 0 | chiamata | |
| ° | 15,9 | ° | ° | 0 | ° | 15,9 | ° | 09/12/2022 | 0 | | |
| ° | 15,8 | ° | ° | 0 | ° | 15,8 | ° | 09/12/2022 | 0 | chiamata | di gaetano, 1981 |
| ° | 15,6 | ° | ° | 0 | ° | 15,6 | ° | 09/12/2022 | 0 | | |
| ° | 15,5 | ° | ° | 0 | ° | 15,5 | ° | 09/12/2022 | 0 | | |
| ° | 15,5 | ° | ° | 0 | ° | 15,5 | ° | 09/12/2022 | 0 | | |
| ° | 15,3 | ° | ° | 0 | ° | 15,3 | ° | 09/12/2022 | 0 | Chiamata | |
| ° | 15,2 | ° | ° | 0 | ° | 15,2 | ° | 09/12/2022 | 0 | | |
| ° | 15,2 | ° | ° | 0 | ° | 15,2 | ° | 09/12/2022 | 0 | | |
| 15/05/2022 | 8,2 | 1 | CHT e RT | 0 | ° | 15,2 | ° | 09/12/2022 | 0 | | |
| ° | 10,3 | ° | ° | 1 | 22/07/2022 | 10,3 | 0 | 22/07/2022 | 0 | morta | |
| ° | 14,6 | ° | ° | 0 | ° | 14,6 | ° | 09/12/2022 | 0 | Chiamata | |

| | | | | | | | | | | | |
|---|---|---|---|---|---|---|---|---|---|---|---|
| ° | 17,1 | ° | ° | 0 | ° | 17,1 | ° | 09/12/2022 | 0 | | |
| ° | 17,1 | ° | ° | 0 | ° | 17,1 | ° | 09/12/2022 | 0 | | |
| ° | 16,9 | ° | ° | 0 | ° | 16,9 | ° | 09/12/2022 | 0 | | |
| ° | 16,8 | ° | ° | 0 | ° | 16,8 | ° | 09/12/2022 | 0 | | |
| ° | 13,8 | ° | ° | 0 | ° | 13,8 | ° | 09/12/2022 | 0 | | |
| ° | 13,8 | ° | ° | 0 | ° | 13,8 | ° | 09/12/2022 | 0 | | |
| ° | 13,1 | ° | ° | 0 | ° | 13,1 | ° | 09/12/2022 | 0 | | |
| ° | 12,9 | ° | ° | 0 | ° | 12,9 | ° | 09/12/2022 | 0 | | |
| ° | 12,2 | ° | ° | 0 | ° | 12,2 | ° | 09/12/2022 | 0 | | |
| | | | | | | | | | | | |
